# Metabolomic and Proteomic Analysis of ApoE4-Carrying H4 Neuroglioma Cells in Alzheimer’s Disease Using OrbiSIMS and LC-MS/MS

**DOI:** 10.1021/acs.analchem.4c01201

**Published:** 2024-07-11

**Authors:** Li Lu, Anna M. Kotowska, Stefanie Kern, Min Fang, Timothy R. Rudd, Morgan R. Alexander, David J. Scurr, Zheying Zhu

**Affiliations:** † School of Pharmacy, The University of Nottingham University Park Campus, Nottingham NG7 2RD, U.K.; ‡ Medicines and Healthcare products Regulatory Agency (MHRA), South Mimms, Blanche Lane EN6 3QG, U.K.

## Abstract

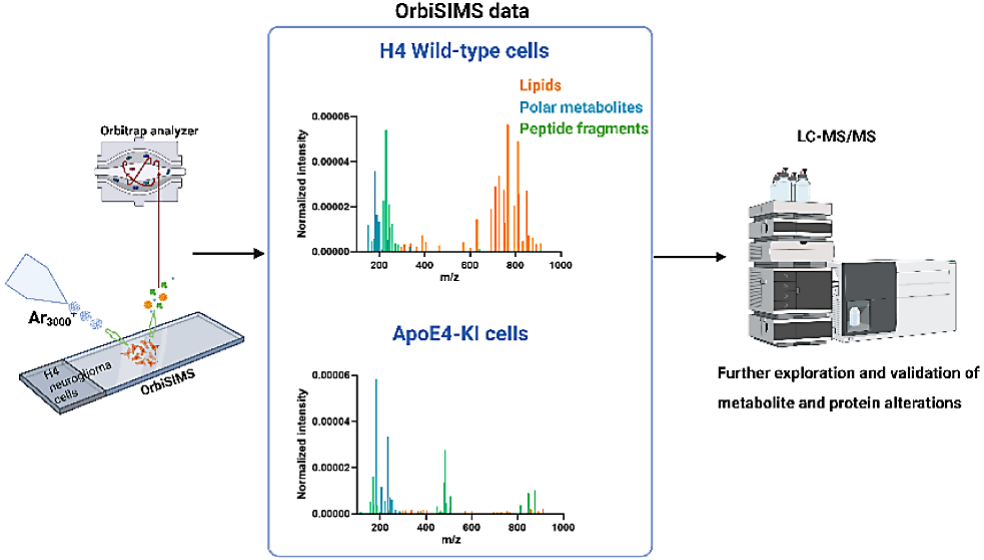

Growing clinical evidence reveals that systematic molecular alterations in the brain occur 20 years before the onset of AD pathological features. Apolipoprotein E4 (ApoE4) is one of the most significant genetic risk factors for Alzheimer’s disease (AD), which is not only associated with the AD pathological features such as amyloid-β deposition, phosphorylation of tau proteins, and neuroinflammation but is also involved in metabolism, neuron growth, and synaptic plasticity. Multiomics, such as metabolomics and proteomics, are applied widely in identifying key disease-related molecular alterations and disease-progression-related changes. Despite recent advances in the development of analytical technologies, screening the entire profile of metabolites remains challenging due to the numerous classes of compounds with diverse chemical properties that require different extraction processes for mass spectrometry. In this study, we utilized Orbitrap Secondary Ion Mass Spectrometry (OrbiSIMS) as a chemical filtering screening tool to examine molecular alterations in ApoE4-carried neuroglioma cells compared to wild-type H4 cells. The findings were compared using liquid chromatography (LC)-MS/MS targeted metabolomics analysis for the confirmation of specific metabolite classes. Detected alterations in peptide fragments by OrbiSIMS provided preliminary indications of protein changes. These were extensively analyzed through proteomics to explore ApoE4’s impact on proteins. Our metabolomics approach, combining OrbiSIMS and LC-MS/MS, revealed disruptions in lipid metabolism, including glycerophospholipids and sphingolipids, as well as amino acid metabolism, encompassing alanine, aspartate, and glutamate metabolism; aminoacyl-tRNA biosynthesis; glutamine metabolism; and taurine and hypotaurine metabolism. Further LC-MS/MS proteomics studies confirmed the dysfunction in amino acid and tRNA aminoacylation metabolic processes, and highlighted RNA splicing alterations influenced by ApoE4.

## Introduction

Alzheimer’s disease (AD) is the most common form of dementia, affecting more than 50 million people worldwide, and is expected to rise by over 150 million cases by 2050.^1^ Late-onset AD (LOAD) represents about 95% of all AD cases. The pathological hallmarks of LOAD are identified as amyloid plaques (Aβ), neurofibrillary tangles (NFTs), glial responses, and neuronal and synaptic loss.^2^ Although it has been more than 100 years since Alois Alzheimer first discovered and described this disease, Aβ and NFTs remain the standard pathological hallmarks for AD. They are targeted by many novel drugs and therapeutics currently under clinical trials, which aim to reduce Aβ production, increase the Aβ clearance, and inhibit the phosphorylation of tau protein.^3^ However, the presence of Aβ and NFTs is not sufficient to fully explain the pathology of AD, indicating they are only pieces of the AD puzzle.^4^,^5^

Genome-wide association studies (GWAS) have found numerous risk genes involved in LOAD, including ApoE4, TREM2, ADAM10, and PLD3, providing us a better understanding of the associated pathophysiological processes.^6^ Among them, ApoE4 is known as the strongest risk gene, with individuals carrying copies of it facing up to 12-fold increased risk of developing AD compared to ApoE4 noncarriers. Understanding the role of ApoE4 should provide insight into mechanisms that drive Alzheimer’s pathogenesis.^7^ There is mounting evidence showing that ApoE4 is associated not only with Aβ deposition, phosphorylation of Tau, and neuroinflammation but also with lipid metabolism, neuron growth, synaptic plasticity, and blood–brain barrier (BBB) integrity.^8^

For the complex pathological mechanisms of ApoE4, growing evidence supports the use of omics techniques in identifying key disease-related molecular alteration and changes related to disease progression.^9^ In the past decades, transcriptomics and proteomics have been applied widely in neurodegenerative diseases to investigate gene or protein expression and help explain phenotypes of diseases. Barisano et al. applied multiomics methods, which included single-nucleus RNA sequencing, phosphoproteome, and proteome analysis, to study the effect of ApoE4 on mice blood–brain barrier (BBB) and synaptic dysfunction.^10^ They found the strong association of early disruption of BBB transcriptome in ApoE4 knock-in mice compared with ApoE3, dysregulation in protein signaling networks in brain endothelium, as well as transcription and RNA splicing suggestive of DNA damage in pericytes. The multiomics studies that combine transcriptome and proteomics analysis provide us a deep view for ApoE4-related molecular changes of mechanism; however, gene expression levels are not necessarily related to protein levels due to the complex and various translation processes. Therefore, in much recent research, metabolomics technology has been applied as a complementary tool to get a better understanding of disease-related molecular alterations.

Over the past decades, metabolomic studies have found that systematic metabolism dysfunction and hypometabolism occur for up to 20 years before the manifestation of AD symptoms.^11^ Lee et al. integrated single-cell RNA sequencing with metabolomics to systematically characterize the role of ApoE4 in microglial response and found that microglial ApoE4 displays the disruption of aerobic glycolysis and lipid metabolism pathways.^12^ A study of targeted LC-MS/MS metabolomics showed that ApoE4 disturbed neuron–astrocyte coupling of fatty acid metabolism and inhibited astrocytic ApoE4 impeding the progression of tau-mediated neurodegeneration.^13^ A large amount of evidence suggests that dysfunction in lipid metabolism pathways is associated with AD pathogenesis, leading to impaired synaptic plasticity, increased Aβ, and hyperphosphorylated tau.^14^ Several studies investigating ApoE4 are mostly based on the transgenic animal model.^15^ Although human iPSC (induced pluripotent stem cells)-derived neural stem cells have been applied in recent preclinical studies to investigate the potential mechanism of ApoE4,^16^ the lack of sensitive and efficient metabolomics screening platforms has limited the ability to simultaneously study various metabolites that may be affected in the presence of ApoE4. Above all, many studies based on LC-MS/MS applied multiple approaches for investigating untargeted metabolomics or targeted methods for compounds of interest whose chemical identity is known before analysis. In this regard, while LC-MS/MS stands as a frequently employed instrument in metabolomics, certain considerations imply limitations of LC-MS/MS in metabolite screening. In addition, the disruptive method required for LC-MS/MS analysis results in the loss of localization information, and the extraction process can lead to metabolite loss and degradation. Localization of molecules provides crucial insights into their biological functions. Therefore, we explore the state-of-the-art Orbitrap Secondary Ion Mass Spectrometry (OrbiSIMS) as an *in situ* metabolomics screening tool due to its high mass resolution and superior surface analysis capabilities. It also has the advantage of being applicable to precious samples that are available only in limited quantities as previously reported.^17^

OrbiSIMS has been applied for metabolomics analysis enabled by the high mass resolving power of the Orbitrap analyzer (>240,000 at *m*/*z* 200).^18^ Passarelli et al. employed OrbiSIMS to image neurotransmitters in mouse hippocampal sections and putatively annotated 127 lipid species, revealing the diverse components and distinct localization of neurotransmitters in brain sections. OrbiSIMS has also been utilized to investigate drug distribution and cellular responses in rat alveolar macrophage cells at the single-cellular level.^18^ Recently, single-cell *in situ* metabolic analysis with OrbiSIMS has been employed to characterize macrophage subsets, providing insight into the phenotypes and immune responses of different macrophage subsets (M0, M1, and M2).^19^ The OrbiSIMS technique has further found application *in situ* protein identification through *de novo* peptide sequencing of proteins.^20^ OrbiSIMS-based metabolomics serves as a crucial platform for *in situ* metabolic analysis of tissue and cells due to its characteristics of minimal sample preparation, small sample size, and rapid analysis time. This enhances the possibility of probing the functions of risk genes in different cell types. Building upon our established ApoE4-expressing H4 neuroglioma cells, the current work aims to explore the capability of OrbiSIMS as a screening tool for identifying alterations in lipids, metabolites, and peptide fragments influenced by the AD risk gene ApoE4 in H4 cells followed by LC-MS/MS validation to confirm the reliability of OrbiSIMS as a screening tool for metabolomic studies. Our results have elucidated the metabolic alterations in H4 neuroglioma cells in the presence of ApoE4, supporting the hypothesis on the low ability of ApoE4 in transporting lipids and discovering amino acid pathways that may be involved in AD. In addition, peptide secondary ion assignments from OrbiSIMS and proteomics analysis by LC-MS/MS further proved the ApoE4-mediated dysfunction of protein biosynthesis, nitrogen compound and amino acids, tRNA aminoacylation metabolic processes, and RNA splicing process.

## Experimental Section

The experimental details of OrbiSIMS measurements, metabolomics data analysis, and proteomics are outlined below. Information regarding cell culture experiments and validation approaches using lipid dye and LC-MS/MS metabolomics is available in Supporting Information S1.

### Cell Sample Preparation for OrbiSIMS

The H4 wild-type cells and ApoE4 KI cells were seeded on glass slides and incubated in complete culture medium for 24 h. After the cells adhered to the glass slides, the medium was discarded, and the cells were washed three times using 150 mM ammonium formate solution. The slides were then frozen in liquid nitrogen and freeze-dried for 48 h. Subsequently, the freeze-dried samples were removed from the −80 °C freezer and allowed to stabilize at room temperature for 1 h before OrbiSIMS analysis.

### OrbiSIMS Analysis

OrbiSIMS analysis was performed using a hybrid SIMS instrument from IONTOF GmbH. The Orbitrap analyzer was calibrated using silver clusters of a silver sample plate, following the method described by Passarelli et al.^18^ For calibration, liquid metal ion gun with Bi_3_^+^ clusters as primary ion species was used in spectrometry mode together with the ThermoFisher Tune software. For the subsequent measurements, the following parameters were employed. The gas cluster ion beam (GCIB) was used with 20 keV Ar_3000_^+^ argon clusters with 20 μm beam diameter as the primary ion source for sputtering of cell samples (around 400–500 cells per analysis). Mass spectra were recorded in full-MS scan in the *m*/*z* range of 75.0–1125.0 in negative polarity. Samples were analyzed at room temperature across a 300 × 300 μm^2^ area using random raster mode with crater size of 384.6 × 384.6 μm^2^ and a mass resolving power of 240,000 at *m*/*z* 200. Cycle time was set to 200 μs, and duty cycle was set to 4.4%. Ar_3000_^+^ primary ion clusters were used with a target current of approximately 200 pA with charge compensation performed using a low-energy (21 eV) electron flood gun. Argon gas flooding was utilized as well to aid with charge compensation, which led to a pressure of 9.0 × 10^–7^ mbar in the main chamber. The maximum injection time was set to 500 ms. The OrbiSIMS collision cell pressure was set to 6.15 × 10^–2^ mbar, and the target potential was set at −278 V during the experiment. Three separative areas were analyzed on each sample, and each measurement lasted 300 scans.

### Cell Sample Preparation for LC-MS/MS Analysis

Incubation media was removed, and the cells were briefly washed with PBS (37 °C). 500 μL of cold methanol (−40 °C) was added to simultaneously quench the metabolism and extract the intracellular metabolites. Cells (at least 1 × 10^6^ cells each sample) were scraped and vortexed vigorously for 1 h on ice and centrifuged at 14000 rpm for 10 min at 4 °C. Supernatants were transferred into new tubes and evaporated using a Jouan Centrifugal Evaporator. The remaining residue was reconstituted in 70 μL of methanol (4 °C) and centrifuged at 14000 rpm for 10 min at 4 °C. 50–70 μL of the extract was transferred into an amber HPLC vial with a glass insert, labeled, and used for LC-MS/MS analysis. 10 μL of each sample was collected and mixed as a pooled QC for untargeted analysis and MS/MS analysis to check the performance of the analytical system. LC-MS/MS analysis can be seen in Supporting Information S1.

### Identification and Annotation of Metabolites

The OrbiSIMS is operated by SurfaceLab7 (IONTOF, Germany). First, we performed a peak search on each raw data, a minimum counts threshold 3000 was set by visual inspection of the spectra that distinguished it from a noise peak. Ions extracted from the spectrum were assigned by applying elemental restrictions with mass deviation <2 ppm for ions >*m*/*z* 95 and 5 ppm for ions <*m*/*z* 95 for molecular formula prediction, which was conducted by using software simsMFP (simsMFP is a MATLAB-based script developed by Edney et al.,^21^ especially for chemical filtering of the OrbiSIMS data set): Lipid search (C_1–230_, H_3–130_, N_0–2_, O_0–20_, P_0–2_, S_0–1_) and other energy-related metabolism (C_3–30_, H_1–40_, N_0–10_, O_0–25_, P_0–3_, S_0–1_), subsequentially matching the chemical formula with LIPID MAPS (https://www.lipidmaps.org/databases/lmsd/overview) and Human Metabolome Database (HMDB) (https://hmdb.ca/spectra/ms/search) database. For peptide-related fragments assignment, simsMFP was also applied for chemical element filtering H4 data set. First, elemental restriction (C_4–100_, H_8–200_, N_0–20_, O_0–20_, S_0–1_) and DBE value (0.1667C_n_ < DBE < 0.6667C_n_) reported by Kotowska et al. were applied to filter out peptide-related peaks for the negative-ion mode.^20^ Another constraint for restricting chemical formulas was element ratios of H/C, N/C, and O/C, which were also based on protein fragments from 16 pure protein samples. More information on the simsMFP filtering can be found in Supporting Information S5.

### Metabolomics Analysis

#### Pathway and Enrichment Analysis

Pathway and enrichment analysis were performed using Metaboanalyst web-based software (https://www.metaboanalyst.ca/). Lipid enrichment was based on 35 super and 464 main chemical class metabolite sets or lipid sets, containing at least 2 entries to match the metabolite set library.

### Gene Ontology Analysis for Metabolomics

To cover the relevant Gene Ontology (GO) metabolic processes that were not mentioned by alternative pathway analysis approaches, IDSL.GOA (gene ontology analysis for metabolomics) was performed for a more comprehensive and accurate analysis of metabolite pathway data (https://goa.idsl.site/goa/#/intro). First, to perform IDSL.GOA analysis, we mapped the significant metabolites to KEGG ID that were used as inputs for IDSL.GOA by using the enrichment function in Metaboanalyst (https://genap.metaboanalyst.ca/MetaboAnalyst/upload/EnrichUploadView.xhtml) and the PubChem Identifier Exchange service.

(https://pubchem.ncbi.nlm.nih.gov/idexchange/idexchange.cgi.)

### Proteomics Data Analysis

For proteomics sample preparation and information on equipment, please see Supporting Information S6.

Protein identification and label-free quantification were achieved using Proteome Discoverer, version 2.5 (Thermo Fisher Scientific). Raw files were searched against the UniProt *Homo sapiens* database (Swiss-Prot with isoforms) using the Sequest HT search algorithm and modified standard processing and consensus workflows. The processing workflow included the mass recalibration node (spectrum files RC) along with the standard spectrum selector, Minora Feature Detector, Sequest HT, and Percolator nodes. The precursor mass tolerance was set to 10 ppm, and the fragment mass tolerance was set to 0.02 Da, with maximum number of missed cleavages set to 2. Carbamidomethylation of cysteine residues (+57.021 Da) was set as static modification, while the oxidation of methionine residues (+15.995 Da) and N-terminal protein modifications of acetyl (+42.011 Da) were set as dynamic modifications. False discovery rate (FDR) tolerances in the Percolator node were set to 0.01 for high confidence and 0.05 for medium confidence. Final results were rescored with percolator and filtered to 1% FDR.

The proteomics network and enrichment analysis were performed by using Cytoscape and StringApp according to the method reported by Doncheva et al.^22^

## Results and Discussion

ApoE4-carried neuroglioma H4 cells were generated using CRISPR/Cas9 gene editing technology and selected by monoclonal culturing. The protein level of ApoE4 and Sanger sequence of monoclonal cultured cell lines showed success in gene editing (Figure S1).

### OrbiSIMS Metabolic Screening of H4 and ApoE4-Carried H4 Cells

**Figure 1:**
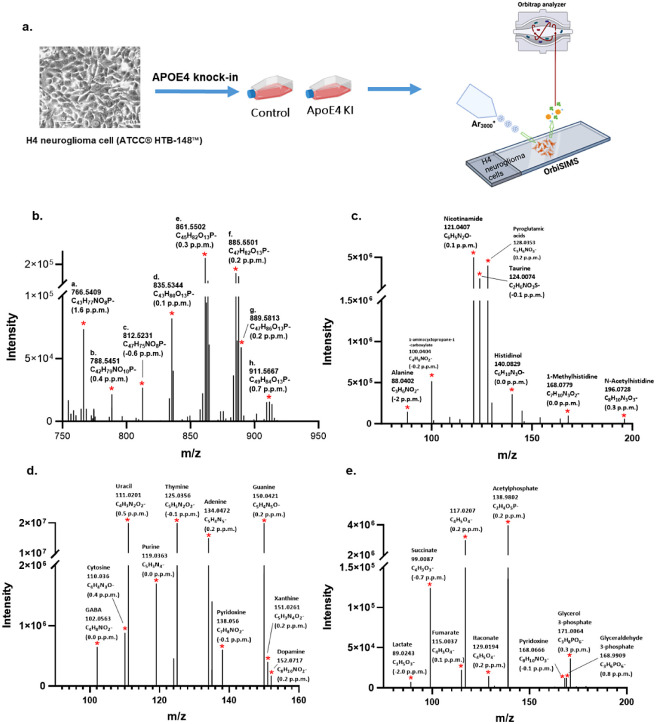
(a) Schematic of sample preparation from wild-type (control) and ApoE4 KI H4 cells. OrbiSIMS spectra for the control H4 cell sample with putative assignments for (b) lipids (only one assignment labeled for each lipid ion in this figure), (c) amino acids and amino acid derivatives, (d) neurotransmitters and nucleic acids, and (e) carbohydrates, carboxylic acids, and other organic acids. Full assignment for (b): PE 38:4(CerP 43:5;O4/PC 35:4/PC O-35:5;O/PE O-38:5;O); PS 36:1(CerP 42:3;O6/PS O-36:2;O); PE 42:9(PC 39:9/PC O-39:10;O/PE O-42:10;O); PI 34:1(LPI 34:2;O/PI O-34:2;O); PI 36:2(PI O-36:3;O); PI 38:4(PI O-38:5;O); PI 38:2(PI O-38:3;O); PI 40:5(PI O-40:6;O).

The ApoE4-KI and wide-type (control) H4 cells were seeded and grown separately on the chamber slide for 24 h, followed by freeze drying under vacuum conditions as described in the Experimental section, prior to OrbiSIMS depth profiling analysis in negative polarity, as shown in Figure 1a.

A comparison of positive and negative polarity data showed that the negative data exhibited a larger number of secondary ions representing amino acids, lipids, and TCA metabolites.^19,23^ All ions were generated and extracted by searching peaks that can differentiate them with noise peaks. Finally, 192 putatively annotated metabolites were detected from the H4 control and ApoE4-KI groups using the OrbiSIMS by assigning chemical formula using simsMFP software:^21^ Lipid search (C_1–230_, H_3–130_, N_0–2_, O_0–20_, P_0–2_, S_0–1_), other energy-related metabolism (C_3–30_, H_1–40_, N_0–10_, O_0–25_, P_0–3_, S_0–1_), followed by matching with LIPIDMAPS and HMDB databases (Table S3).

These 192 putatively annotated metabolites are classified into nine groups based on their major chemical classes and involvement in metabolism pathways, including lipids, amino acids and derivatives, carbohydrates and carbohydrate conjugates, carboxylic/dicarboxylic acids and derivatives, purines nucleotides and purine derivatives, pyridines and derivatives, pyrimidines and pyrimidine derivatives, neurotransmitters, and other organic compounds.

The numbers of each class of annotated metabolites are present in Table 1. It is notable that lipids constitute a substantial portion of metabolites. This observation is rationalized by the inherent capability of SIMS in effectively analyzing intact lipids. Additional details about these lipid classes are presented in Supporting Information S2. The depth profiles of select secondary ions are depicted in Figure S2, offering insights into the distinct localization or enrichment patterns of molecules within the cells. Phospholipids are essential components of the cell that modulate membrane stability, transmit cellular signals, and stabilize synapses.^24^ The phospholipid ions (assigned as C_n_H_n_O_n_P^–^) observed on the cell surface in Figure S2A exhibit a higher concentration at the beginning of the analysis, which then gradually decreases with increasing analysis depth. This is consistent with the hypothesis that phospholipids are most abundant in the membrane. Similarly, the depth profile of fatty acid ions (C_n_H_n_O_2_−) follows the same trend as phospholipids, as the fatty acid fragment can be derived from intact phospholipids.

**Table 1:** Classification of Annotated Metabolites Detected from ApoE4 KI and Wild-Type H4 Cells by OrbiSIMS

**classification of metabolites**	**number of metabolites (192)**
lipids	115 (86 ions have multiple assignment)
amino acids and derivatives	27
carbohydrates and carbohydrate conjugates	6
carboxylic/Dicarboxylic acids and derivatives	5
purines nucleotides and purine derivatives	11
pyridines and derivatives	4
pyrimidines and pyrimidine derivatives	11
neurotransmitters	3
other organic compounds	10

Figure 1b shows the assignment of a targeted series of lipid ions that were detected from the H4 control and ApoE4-KI groups, including putatively annotated lipids such as C_43_H_77_NO_8_P^–^ (CerP 43:5;O4/PC 35:4/PC O-35:5;O/PE 38:4/PE O-38:5;O, *m*/*z* 766.5409), C_42_H_79_NO_10_P^–^ (CerP 42:3;O6/PS 36:1/PS O-36:2;O, *m*/*z* 788.5451), and C_45_H_82_O_13_P^–^ (PI 36:2/PI O-36:3;O, *m*/*z* 861.5502). Overall, 115 lipids (86 ions have multiple assignment) have been annotated from H4 wild-type and ApoE4-KI cells, including the main 11 classes (FA, PA, PE, PS, PG, PI, SM, CL, CPA, CerP, HexCer) (as shown in Table 1), which were also largely detected in a recent study of single-cell macrophage metabolomics using OrbiSIMS.^19^ In addition, in the OrbiSIMS analysis of the H4 control and ApoE4 KI cells, amino acids and their derivatives were observed, including asparagine, glutamine, and taurine (Figure 1c). Neurotransmitters (GABA, dopamine), pyrimidines and derivatives (cytosine, thymine, uracil), and purine and derivatives (guanine, adenine, hypoxanthine) were assigned in our study as well (Figure 1d). Such ions have been observed in previous studies by Passarelli et al.^18,19^ However, purine and xanthine were not detected in this previous work. Figure 1e shows the assigned ions for carbohydrates (glyceraldehyde 3-phosphate), carboxylic acids (fumarate), and other organic acids (lactate) that were not detected in previous studies. Overall, metabolic screening of H4 cells using OrbiSIMS allows us to obtain a range of chemical classes *in situ* covering polar metabolites and low polar lipids.

### OrbiSIMS Pathway Analysis and Signatures of H4 Cells Affected by ApoE4

**Figure 2:**
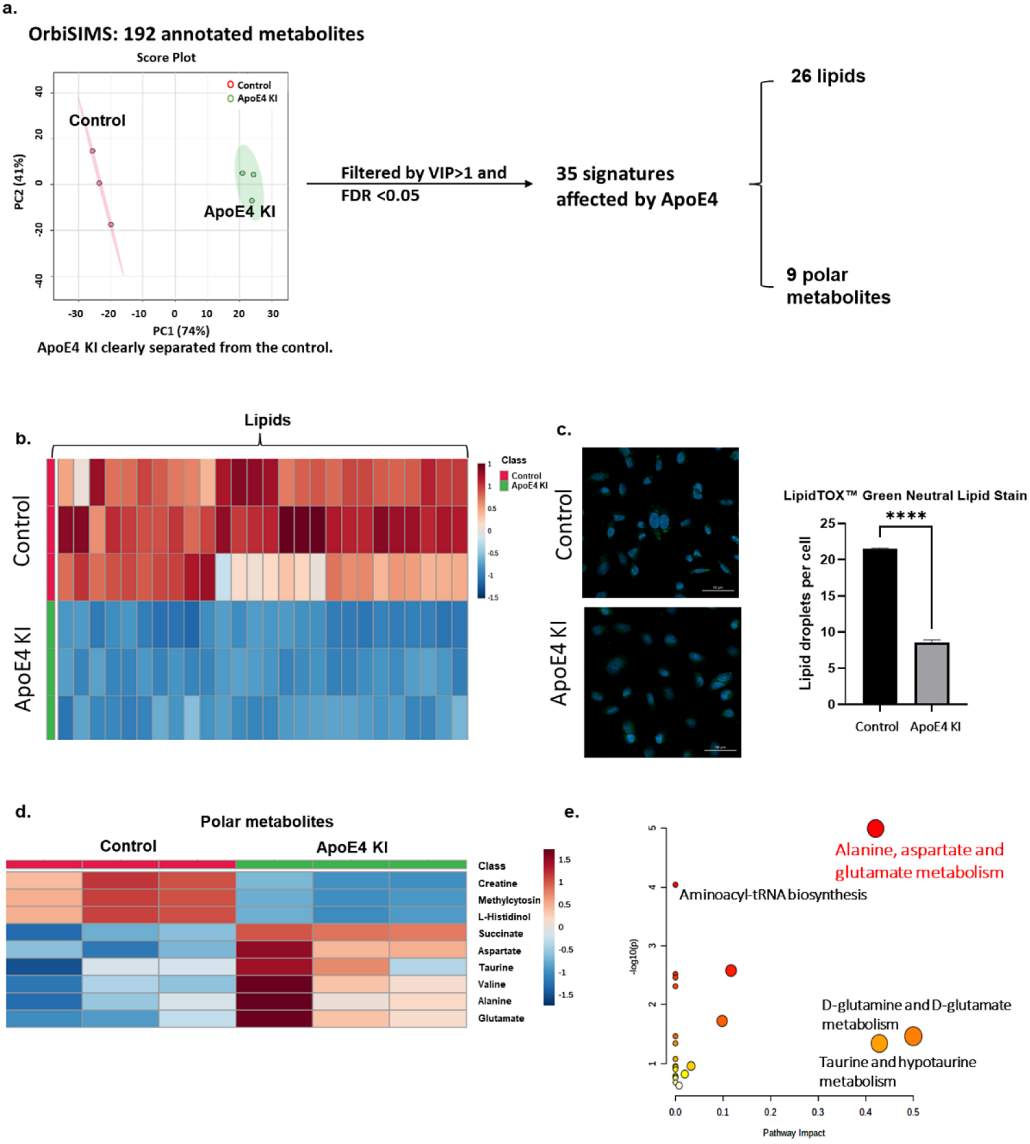
(a) Score plot of PLS-DA of OrbiSIMS data, followed by the lipid classification and pathway analysis for 35 significant metabolites. (b) Heatmap of 26 lipid signatures between control and ApoE4 KI group. (c) Fluorescence image of LipidTOX Green Neutral Lipid Stain and relative intensity of this fluorescence. (d) Heatmap of 9 signatures between control and ApoE4 KI group. (e) Pathway analysis of 9 out of 35 signatures except for 26 lipids.

To further explore the ApoE4-related metabolic profile, PLS-DA (partial least-squares discriminant analysis) of all ions from ApoE4 KI were used for discriminative analysis compared with control cells. The scores plot in Figure 2a shows the divergence in metabolic profiling between the control and ApoE4-expressing cells. The features with variable importance for projection (VIP) > 1 and false discovery rate (FDR) adjusted *P* < 0.05 were considered statistically significant. From this analysis, we obtained 35 metabolic signatures (Table S4) for further pathway analysis, and their relative intensities are shown in Figure 2b,d. Of these secondary ions, 26 out of 35 are lipids that are depleted in the ApoE4-carried cells compared to control cells (Figure 2a,b). The assignments for those 26 lipid ions were mainly categorized as the classes of glycerophospholipids (PA, PE, PS, PC, PG, PI) and sphingolipids (SM, CerP, Hexcer), indicating the disruption of the glycerophospholipid and sphingolipids by ApoE4 (Table S4). To substantiate the impact of ApoE4 on lipid alterations, cellular staining was performed using the LipidTOX neutral lipid dye. This resulted in a notable decrease in lipid droplets within the ApoE4 KI group compared to control (Figure 2c). The results are consistent with the low ability of ApoE4 in transporting lipids and cholesterol, resulting in the dysfunction of the intracellular lipid state which is associated with aggravation of AD progression.^25^ Furthermore, ApoE presents as two states in the cell, lipidated ApoE and free ApoE, and the function of ApoE is highly dependent on its lipidated degree. A recent AD brain-based study found that the lower level of lipidated ApoE lipoprotein in the E4 carrier impacts Aβ binding to microglia and subsequently affects microglia clearance in response to Aβ.^26^ Our results further support this hypothesis that ApoE4 triggers metabolic disorders of AD by affecting the abundance of glycerophospholipids.

In addition, the remaining nine secondary ions assigned by comparison with the HMDB are enriched in the ApoE4 KI cells compared to control, mostly in alanine, aspartate, and glutamate metabolism (Figure 2d,e), suggesting the metabolic disorders of these three amino acids. Additionally, aminoacyl-tRNA biosynthesis, glutamine metabolism, and taurine and hypotaurine metabolism have also been affected in the ApoE4 cells compared to wild-type cells. Levels of alanine, aspartate, and glutamate all increase in the ApoE4 KI group, which partially contrasts with other metabolomics studies showing their low concentrations in AD patients.^27^

Glutamate is one of the excitatory neurotransmitters in the human brain and plays a crucial role in multiple cerebral functions such as memory, cognition, and motor behavior.^28^ A low level of glutamate has been detected in the aged human brain and AD patients, indicating the vital role of glutamate as a biomarker of brain functions. Aside from glutamate, the level of aspartate in AD patients’ brains was decreased compared with the normal brain in previous studies.^29^

Aspartate has many biochemical roles, donating amino groups to the urea cycle, participating in gluconeogenesis, as well as stimulating NMDA (N-methyl-d-aspartate) receptors, which play a crucial role in synaptic modification.^30^

In mammals, alanine is used to make proteins, to convert glucose into energy and repair muscle tissue.^31^ The level of alanine during the development of AD is controversial due to the different functions of D- and l-alanine. Total alanine content in Alzheimer’s gray matter has shown a very significant decrease compared with normal tissues.^32^ In the OrbiSIMS data used in our study, D and l enantiomers of alanine could not be differentiated; therefore, the alanine level is supposed to be lower in ApoE4-carried H4 cells.

Overall, the results indicated that OrbiSIMS could detect an extensive range of metabolites, which provides a quick approach to metabolically profile cells in diseased states. It further supported the capability of OrbiSIMS to provide an objective analysis and direct toward chemical species of interest, which vary between different types of samples. The lipids obtained through OrbiSIMS analysis have been validated by lipid dye staining, showing trends consistent with ApoE4 effects. However, amino acids (alanine, aspartate, and glutamate) show contrasting trends compared to other metabolomics studies. Due to SIMS’s tendency to generate numerous fragment species during secondary ion production, we utilized LC-MS/MS, offering a softer ionization technique for more reliable identification of these polar metabolites.

### LC-MS/MS of Polar Metabolites Analysis

To validate the findings from the OrbiSIMS analysis, particularly those related to amino acids, as shown in Figure 2d, and to enhance our understanding of additional polar metabolites that may be affected, we employed hydrophilic interaction chromatography (HILIC) LC-MS/MS to quantify endogenous polar metabolites. Details of the LC-MS/MS analysis and metabolite identification are provided in Supporting Information S3. In total, 121 metabolites were identified by matching accurate mass, retention time, and MS/MS spectra. Specifically, we examined alterations in alanine, aspartate, and glutamate in ApoE4 KI cells compared to control cells. The levels of alanine, aspartate, and glutamate decreased in the presence of ApoE4, which aligns with clinical metabolomics findings yet contrasts with the OrbiSIMS results previously noted. Furthermore, pathway analysis of the LC-MS/MS data suggests that taurine and hypotaurine metabolism is the most significantly affected pathway in ApoE4 KI cells.

**Figure 3:**
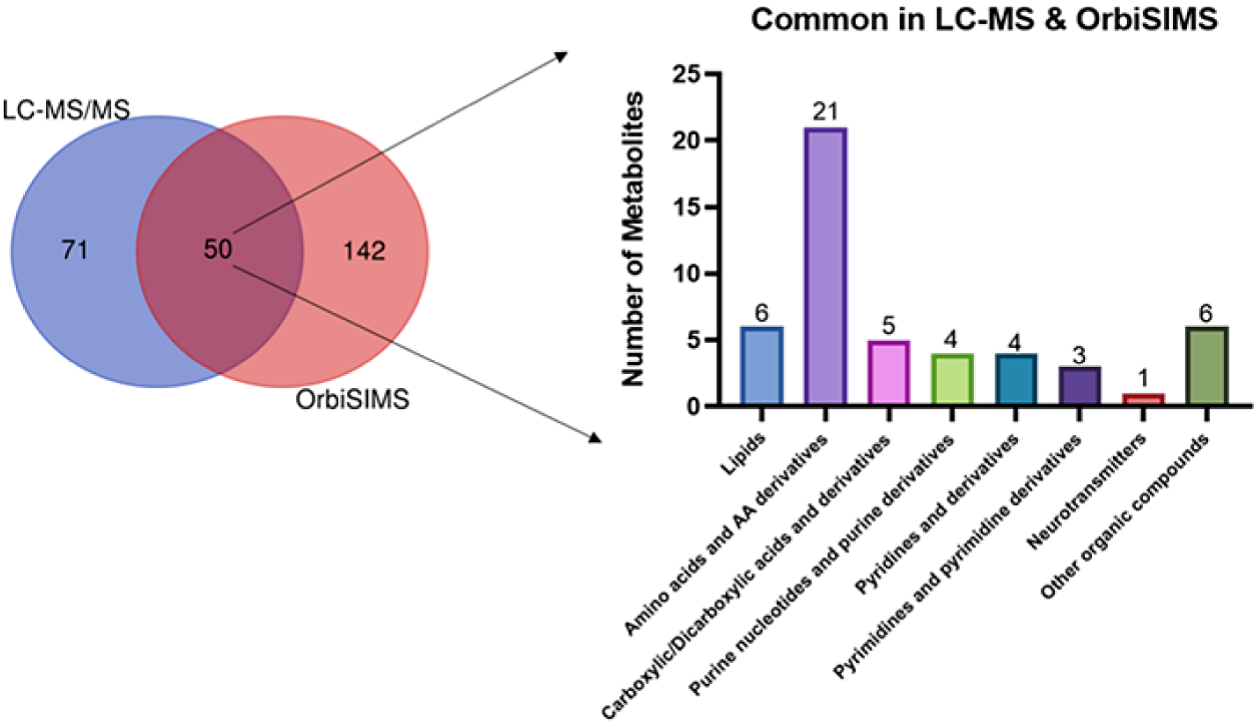
Venn diagram of identified metabolites in LC-MS/MS compared with putatively annotated metabolites in OrbiSIMS, in which 50 metabolites are commonly detected from both LC-MS/MS and OrbiSIMS.

Additionally, we compared the metabolites detected from LC-MS/MS with OrbiSIMS to further evaluate the performance of OrbiSIMS on metabolomics. The Venn diagram in Figure 3 suggested that 50 metabolites are detected by both methods, mostly free amino acids and amino acids derivatives (20 species). Interestingly, most of them show the same enrichment/depletion between the control and ApoE4 KI groups (Figure S5). More information on the comparison of OrbiSIMS and LC-MS/MS results can be found in Supporting Information S4.

The conclusion of the above metabolomic study is that OrbiSIMS demonstrated broad coverage in detecting various chemical classes, including polar and nonpolar metabolites. This confirms the qualitative and semiquantitative capabilities of OrbiSIMS. However, the production of small fragments hampers the quantitative ability of OrbiSIMS. Fragments produced from higher-mass molecules might share the same chemical structure with some low-mass molecules, making them mix, which is challenging to differentiate in the spectrum. Therefore, LC-MS/MS was applied to further confirm those lower-mass-range metabolites and complement the OrbiSIMS data. Combining both methods, we identified that the presence of ApoE4 in H4 cells significantly affects the lipid metabolism (glycerophospholipids and sphingolipids) and amino acid metabolism, including alanine, aspartate, and glutamate metabolism; aminoacyl-tRNA biosynthesis; glutamine metabolism; and taurine and hypotaurine metabolism.

### Proteins Affected by ApoE4 in H4 Cells

Aside from the metabolomics application of OrbiSIMS, we can filter out peptide-related peaks from the OrbiSIMS data set based on elemental composition (using simsMFP). Although peptide assignment and protein identification have been reported,^20,21^ peptide assignment in such complex cell samples has not been studied yet. To do this, we applied simsMFP to filter out peptide-related peaks based on elemental composition from each of the control and ApoE4 KI samples. Results of peptide fragments showed the effect of ApoE4 on various protein biological function, and detailed method and information of peptide assignment using OrbiSIMS are summarized in Supporting Information S5.

The detection of peptides by OrbiSIMS provides clues on the alteration of peptides in the ApoE4-carried H4 cells. Fifty-two peptide fragments were commonly detected from both control and ApoE4 KI cells but showed much lower intensities in control cells, possibly indicating the ApoE4-mediated dysfunction of protein biosynthesis. Moreover, 38 peptide fragments were uniquely present in control cells and 43 in ApoE4 cells, suggesting that some proteins were affected by ApoE4, possibly through changes in protein expression levels or modifications.

The changes in assigned peptide peaks between ApoE4 KI and control samples provide an overview of how ApoE4 may affect protein levels. Common peptides in both groups, which all show lower levels in ApoE4 KI, might suggest a disruption in general protein levels. Additionally, peptides uniquely detected either in control or ApoE4 KI cells indicate potential changes in protein modifications or protein levels. These observations could provide crucial insights for further proteomics studies.

Therefore, to further investigate the role of ApoE4-mediated dysfunction of protein biosynthesis in the protein expression level, proteomics was performed for H4 control and ApoE4 KI cells. The experimental details of proteomics are described in Supporting Information S6. Proteomics identified a total of 2925 proteins from the two groups of samples. Based on the different expression levels of these proteins between ApoE4-carried and wild-type H4 cells, 1503 proteins have been selected by filtering the protein with ApoE4/control ratios of >2 and <0.5. Network building was performed using the STRING database by inputting these 1503 proteins; 1458 protein IDs were recognized by STRING protein database.

Starting with the list of 1458 proteins with significantly regulated features in the study, we first generated the corresponding STRING protein network in Cytoscape. Then, the log ratios of differentially expressed proteins between ApoE4-carried cells and wild-type H4 cells can be visualized on the network nodes. Here, we used a blue-white-red color gradient to highlight the nodes with low or high log ratios (Figure S8).

**Figure 4:**
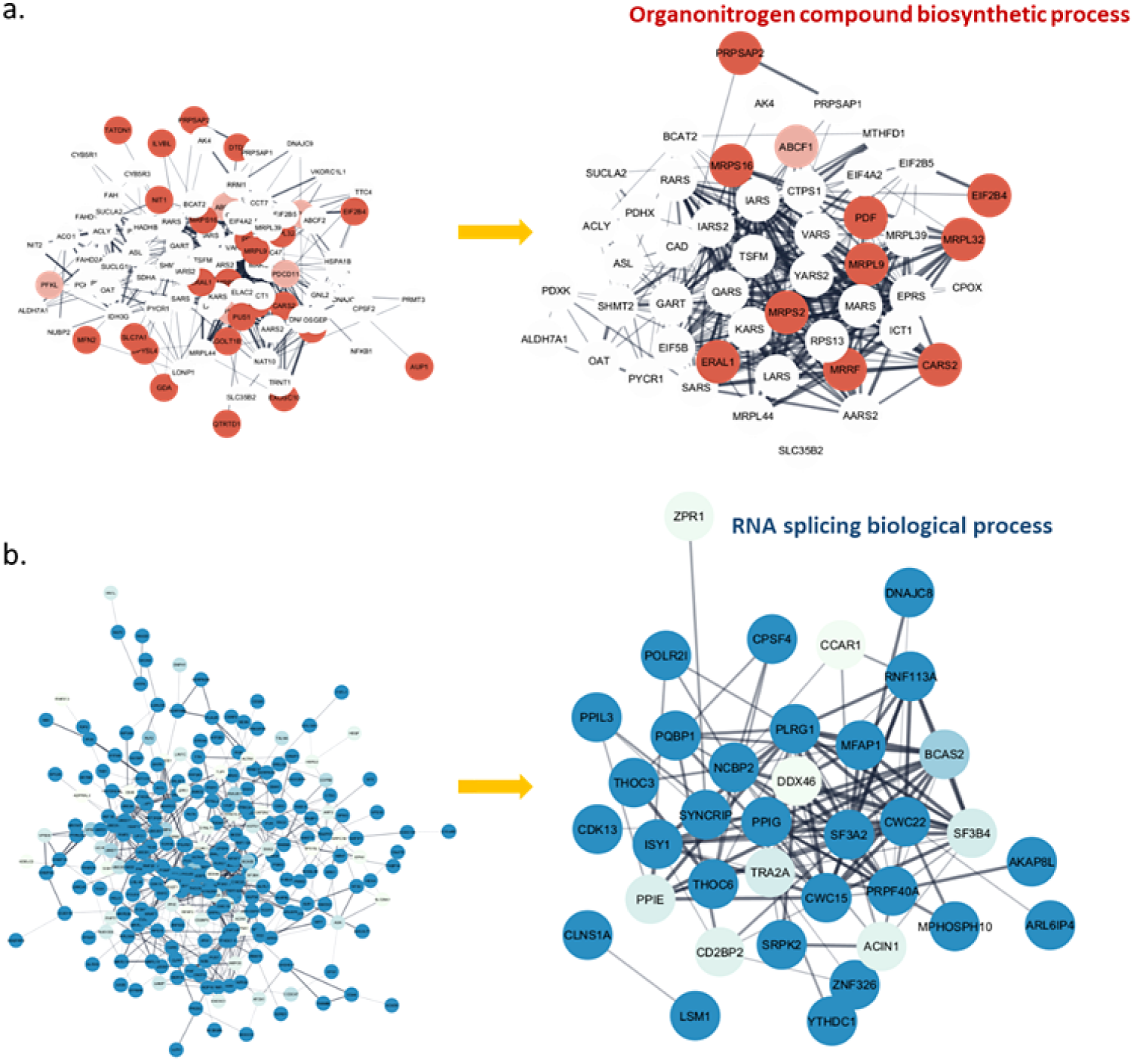
Clustered protein association network of upregulated proteins (a) and downregulated proteins (b), along with the proteins involved in their corresponding Gene Ontology (GO) biological processes. Log ratios between the ApoE4-carried group and the control group for each protein were mapped to the nodes using a blue–white–red gradient. Full details of those proteins are listed in Tables S12 and S14.

Next, to functionally characterize these up or down-regulated proteins, we used stringApp to perform functional enrichment analysis. For 1161 upregulated proteins (19864 interactions) affected by ApoE4, Markov clustering (MCL) algorithms have been performed to group the proteins in the network based on their interactions from STRING (inflation value: 4.0). After MCL simplification, only 1495 interactions within clusters are retained. Next, we only focused on the most cluster (101 proteins with 611 interactions) in the upregulated protein network. The details of those 101 proteins are listed in Table S13. To functionally characterize the cluster, we used stringApp to perform functional enrichment analysis, resulting in a list of 96 statistically significant terms (FDR < 0.05) spanning two categories: Gene Ontology (GO) Biological Process and KEGG Pathways (listed in Table S14). Of these, the most significant GO biological processes were the organonitrogen compound biosynthetic process (Figure 4a), cellular amino acid metabolic process, mitochondrial gene expression, nucleobase-containing compound metabolic process, and tRNA processing, suggesting an influence of ApoE4. The proteins that participate in metabolic processes of H4 cells have been upregulated. In addition, the KEGG pathways, including aminoacyl-tRNA biosynthesis; biosynthesis of amino acids; and alanine, aspartate, and glutamate metabolism, fit well with previous metabolomics finding in this study that is consistent with dysfunction of aminoacyl-tRNA biosynthesis and amino acids affected by ApoE4.

For 297 downregulated proteins (715 interactions) affected by ApoE4, Markov clustering (MCL) algorithms have been performed to group the proteins in the network based on their interactions from STRING (inflation value: 4.0). After MCL simplification, we focused on the most cluster (255 proteins with 706 interactions) in the downregulated protein network (Table S15). In terms of downregulated proteins after ApoE4 KI, the enrichment analysis resulted in a list of 69 (downregulated) statistical terms (Table S16). Of these, the two most significant GO biological processes were RNA splicing and RNA processing (Figure 4b). The downregulated enrichment pathways affected by ApoE4 fit well with the finding by Towfique et al. and Ping-Chung et al.^33^ that the RNA splicing dysfunction is related to AD pathology. In addition, our KEGG pathways identified significant enrichment of spliceosome, endocytosis, and RNA transport pathways. Our proteomics data supports the idea that ApoE4 is associated with the disruption of gene transcription and translation; however, the exact mechanism is not clear and needs to be further confirmed.

### GO Analysis of Metabolomics and Proteomics Affected by ApoE4

Finally, to comprehensively interpret cross-connection between proteomics data with metabolomics, we applied GO analysis for metabolomics. Gene Ontology (GO) analysis is a powerful tool used in genomics and proteomics to categorize and understand the functions of genes and proteins within biological systems. Typically, the biological interpretation of metabolomics data involves pathway analysis and metabolite set enrichment analysis. However, the biological functions of metabolites and metabolic pathways are not comprehensively covered and can vary across different databases, which may lead to misunderstandings and poor biological interpretations. Mahajan et al.^34^ reported an online GO tool for metabolomic data sets that can identify important GO metabolic processes not covered in existing pathway databases. Therefore, we applied IDSL.GOA to our metabolomic data sets to gain deeper biological insights.

**Figure 5:**
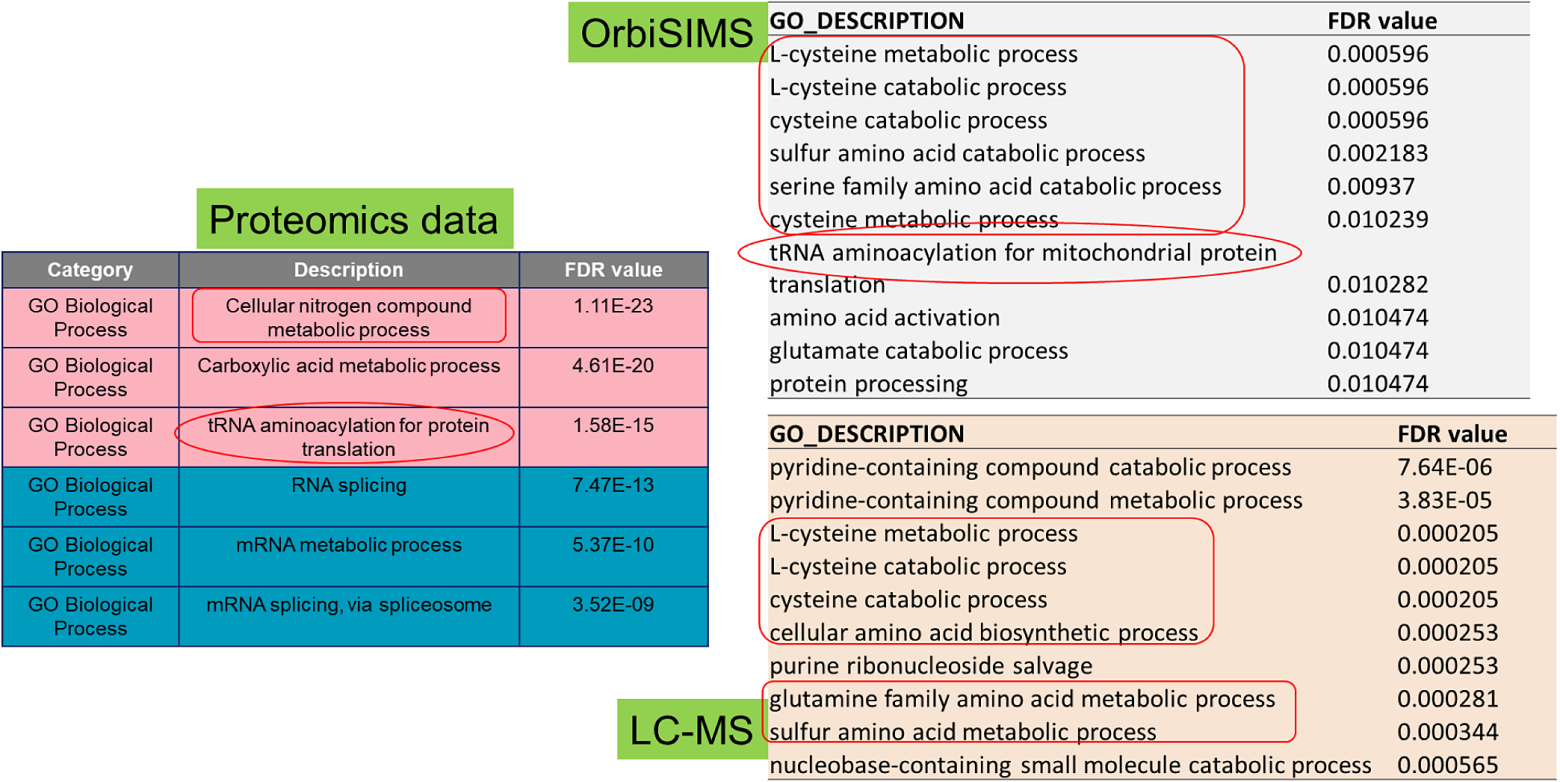
GO analysis of metabolomics data sets from OrbiSIMS and LC-MS/MS, and comparison with proteomics GO analysis.

We built up metabolite data sets that were significantly different between the ApoE4 KI group and the control group from OrbiSIMS (35) and LC-MS/MS (40), respectively. Out of this list, 15 (OrbiSIMS) and 28 (LC-MS/MS) metabolites had KEGG identifiers available and were used as input for IDSL.GOA analysis.^34^ The GO analysis for OrbiSIMS metabolomics suggested a total of 17 GO processes that were changed, such as the cysteine, sulfur amino acid catabolic processes (Table S17), and tRNA aminoacylation for mitochondrial protein that were significantly affected by ApoE4. For LC-MS/MS, 120 GO biological processes were affected by ApoE4, which involved pyridine-containing compound process, cysteine metabolic process, cellular amino acid biosynthesis process, etc. (Table S18). We found many overlapping metabolic processes from OrbiSIMS and LC-MS/MS data sets, which are all involved in amino acid metabolism (Figure 5). Furthermore, by comparing GO analysis of metabolomics with proteomics GO analysis, cellular nitrogen compound metabolic process and tRNA aminoacylation process are found commonly from both omics results. Overall, GO analysis of metabolomics by using IDSL.GOA provides more biological function information from metabolites study, as well as helps build the link between metabolomics and proteomics, overall suggesting that nitrogen compounds, amino acids, and tRNA aminoacylation metabolic processes play important roles in ApoE4-mediated molecular alterations in AD.

## Conclusions

Label-free detection of metabolites and peptide fragments can be achieved with direct analysis of a single sample using OrbiSIMS. This has the advantage of a single-analysis approach over LC-MS/MS, which requires three different sample extraction and separation processes for lipids, metabolites, and peptides. This demonstrates the capability of OrbiSIMS as an efficient screening tool for molecular alteration in cells, especially for metabolomics analysis. Lipids are particularly well covered, while amino acids and other polar analytes could be putatively annotated from the OrbiSIMS spectrum; multiple small fragments produced during the SIMS process complicate the assignment of molecular ions. LC-MS/MS can be utilized to further confirm and complement OrbiSIMS.

Glycerophospholipid biosynthesis is the most affected pathway in the ApoE4-carried neuroglioma H4 cells, followed by the dysfunction of alanine, aspartate, and glutamate metabolism and alanine, taurine, and hypotaurine metabolism. These findings could help to further understand the metabolisms involved in ApoE4-related pathogenesis of AD. Peptide fragments have also been assigned from wild-type and ApoE4-carried cells, indicating the disorder of protein biosynthesis by ApoE4. However, H4 neuroglioma cells are not derived from Alzheimer's disease (AD) patients, and the gene editing used to activate ApoE4 in these cells does not fully elucidate the clinical and genetic mechanisms of ApoE4. Additionally, the function of ApoE4 exhibits significantly different effects on various cell types, including neurons, microglia, and astrocytes. Consequently, more clinically relevant samples and studies are necessary to comprehensively understand the role of ApoE4 in AD.

**Figure 6:**
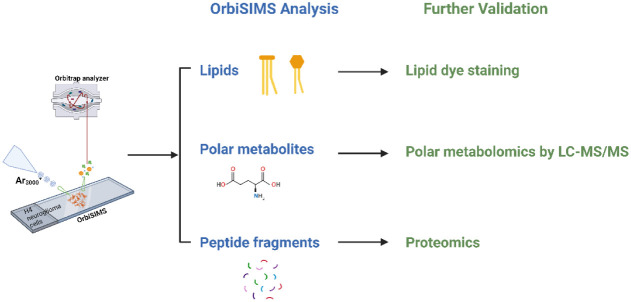
Novel workflow of OrbiSIMS as a screening tool suggests further validation methods for each type of metabolite. Initially, OrbiSIMS depth profile analysis is conducted to obtain spectral information, including lipids, polar metabolites, and peptide fragments. If differentiated levels of lipids are observed under disease or treatment conditions, they are further validated or confirmed through lipid dye staining or lipidomics studies. Similarly, alterations in polar metabolites can be complemented and further explored using LC-MS/MS metabolomics. Finally, the applicability of OrbiSIMS to detect peptide fragments provides initial indications of protein-level changes, which can be further investigated by proteomics analysis.

Collectively, by applying the novel workflow as shown in Figure 6 that uses OrbiSIMS as a metabolomics screening tool and LC-MS/MS as a complementary approach, we found that in the presence of ApoE4, the glycerophospholipid metabolism of H4 cells is less effective than wild-type H4 cells, followed by hypometabolism of alanine, aspartate, glutamate, and taurine. We also proved the applicability of OrbiSIMS for peptide detection, which can be further used to investigate the defective protein biosynthesis mediated by ApoE4. In addition, we performed proteomics analysis that indicates the dysfunction of the organonitrogen compound metabolic process and RNA splicing affected by ApoE4. The overlapping GO analysis of metabolomics with proteomics data further indicated the importance of amino acids and tRNA aminoacylation metabolic process involved in ApoE4 pathology. Overall, it suggests the key role of ApoE4 played in cellular metabolism and the translation process associated with the pathological mechanism of AD.

## Supporting Information Available

Detailed experimental information on cell culture, Western blotting, lipid staining, LC-MS/MS measurement and data analysis, as well as proteomics data analysis; detailed assignment peak list of OrbiSIMS and LC-MS/MS; raw mass spectrometry data of OrbiSIMS and LC-MS/MS; and identified protein list (PDF)

## References

[ref1] Alzheimer’s A. 2019 Alzheimer’s disease facts and figures. Alzheimer’s Dement. 2019, 15 (3), 321–387. 10.1016/j.jalz.2019.01.010.

[ref2] a.Zhang Q.; Sidorenko J.; Couvy-Duchesne B.; Marioni R. E.; Wright M. J.; Goate A. M.; Marcora E.; Huang K.-L.; Porter T.; Laws S. M.; et al. Risk prediction of late-onset Alzheimer’s disease implies an oligogenic architecture. Nat. Commun. 2020, 11 (1), 4799 10.1038/s41467-020-18534-1. 32968074 PMC7511365

[ref3] Cummings J.; Zhou Y.; Lee G.; Zhong K.; Fonseca J.; Cheng F. Alzheimer’s disease drug development pipeline: 2023. Alzheimer’s Dement. Transl. Res. Clin. Interventions 2023, 9 (2), e12385 10.1002/trc2.12385. PMC10210334 37251912

[ref4] Li T.; Lu L.; Pember E.; Li X.; Zhang B.; Zhu Z. New Insights into Neuroinflammation Involved in Pathogenic Mechanism of Alzheimer’s Disease and Its Potential for Therapeutic Intervention. Cells 2022, 11 (12), 1925 10.3390/cells11121925. 35741054 PMC9221885

[ref5] van der Flier W. M.; de Vugt M. E.; Smets E. M. A.; Blom M.; Teunissen C. E. Towards a future where Alzheimer’s disease pathology is stopped before the onset of dementia. Nat. Aging 2023, 3 (5), 494–505. 10.1038/s43587-023-00404-2. 37202515

[ref6] Bellenguez C.; Küçükali F.; Jansen I. E.; Kleineidam L.; Moreno-Grau S.; Amin N.; Naj A. C.; Campos-Martin R.; Grenier-Boley B.; Andrade V.; et al. New insights into the genetic etiology of Alzheimer’s disease and related dementias. Nat. Genet. 2022, 54 (4), 412–436. 10.1038/s41588-022-01024-z. 35379992 PMC9005347

[ref7] Martens Y. A.; Zhao N.; Liu C.-C.; Kanekiyo T.; Yang A. J.; Goate A. M.; Holtzman D. M.; Bu G. ApoE Cascade Hypothesis in the pathogenesis of Alzheimer’s disease and related dementias. Neuron 2022, 110 (8), 1304–1317. 10.1016/j.neuron.2022.03.004. 35298921 PMC9035117

[ref8] Chen Y.; Durakoglugil M. S.; Xian X.; Herz J. ApoE4 reduces glutamate receptor function and synaptic plasticity by selectively impairing ApoE receptor recycling. Proc. Natl. Acad. Sci. U. S. A. 2010, 107 (26), 12011 10.1073/pnas.0914984107. 20547867 PMC2900641

[ref9] a.Gowda G. A. N.; Zhang S.; Gu H.; Asiago V.; Shanaiah N.; Raftery D. Metabolomics-based methods for early disease diagnostics. Expert Rev. Mol. Diagn. 2008, 8 (5), 617–633. 10.1586/14737159.8.5.617. 18785810 PMC3890417

[ref10] Barisano G.; Kisler K.; Wilkinson B.; Nikolakopoulou A. M.; Sagare A. P.; Wang Y.; Gilliam W.; Huuskonen M. T.; Hung S.-T.; Ichida J. K.; et al. A “multi-omics” analysis of blood–brain barrier and synaptic dysfunction in APOE4 mice. J. Exp. Med. 2022, 219 (11), e20221137 10.1084/jem.20221137. 36040482 PMC9435921

[ref11] Dubois B.; Hampel H.; Feldman H. H.; Scheltens P.; Aisen P.; Andrieu S.; Bakardjian H.; Benali H.; Bertram L.; Blennow K.; et al. Preclinical Alzheimer’s disease: Definition, natural history, and diagnostic criteria. Alzheimer’s Dement. 2016, 12 (3), 292–323. 10.1016/j.jalz.2016.02.002. 27012484 PMC6417794

[ref12] Lee S.; Devanney N. A.; Golden L. R.; Smith C. T.; Schwartz J. L.; Walsh A. E.; Clarke H. A.; Goulding D. S.; Allenger E. J.; Morillo-Segovia G.; et al. APOE modulates microglial immunometabolism in response to age, amyloid pathology, and inflammatory challenge. Cell Rep. 2023, 42 (3), 112196 10.1016/j.celrep.2023.112196. 36871219 PMC10117631

[ref13] Qi G.; Mi Y.; Shi X.; Gu H.; Brinton R. D.; Yin F. ApoE4 Impairs Neuron-Astrocyte Coupling of Fatty Acid Metabolism. Cell Rep. 2021, 34 (1), 108572 10.1016/j.celrep.2020.108572. 33406436 PMC7837265

[ref14] aLiu Y.; Thalamuthu A.; Mather K. A.; Crawford J.; Ulanova M.; Wong M. W. K.; Pickford R.; Sachdev P. S.; Braidy N. Plasma lipidome is dysregulated in Alzheimer’s disease and is associated with disease risk genes. Transl. Psychiatry 2021, 11 (1), 344 10.1038/s41398-021-01362-2. 34092785 PMC8180517

[ref15] Balu D.; Karstens A. J.; Loukenas E.; Maldonado Weng J.; York J. M.; Valencia-Olvera A. C.; LaDu M. J. The role of APOE in transgenic mouse models of AD. Neurosci. Lett. 2019, 707, 134285 10.1016/j.neulet.2019.134285. 31150730 PMC6717006

[ref16] Zhao J.; Fu Y.; Yamazaki Y.; Ren Y.; Davis M. D.; Liu C.-C.; Lu W.; Wang X.; Chen K.; Cherukuri Y.; et al. APOE4 exacerbates synapse loss and neurodegeneration in Alzheimer’s disease patient iPSC-derived cerebral organoids. Nat. Commun. 2020, 11 (1), 5540 10.1038/s41467-020-19264-0. 33139712 PMC7608683

[ref17] Meurs J.; Scurr D. J.; Lourdusamy A.; Storer L. C. D.; Grundy R. G.; Alexander M. R.; Rahman R.; Kim D. H. Sequential Orbitrap Secondary Ion Mass Spectrometry and Liquid Extraction Surface Analysis-Tandem Mass Spectrometry-Based Metabolomics for Prediction of Brain Tumor Relapse from Sample-Limited Primary Tissue Archives. Anal. Chem. 2021, 93 (18), 6947–6954. 10.1021/acs.analchem.0c05087. 33900724

[ref18] Passarelli M. K.; Pirkl A.; Moellers R.; Grinfeld D.; Kollmer F.; Havelund R.; Newman C. F.; Marshall P. S.; Arlinghaus H.; Alexander M. R.; et al. The 3D OrbiSIMS—label-free metabolic imaging with subcellular lateral resolution and high mass-resolving power. Nat. Methods 2017, 14 (12), 1175–1183. 10.1038/nmeth.4504. 29131162

[ref19] Suvannapruk W.; Edney M. K.; Kim D.-H.; Scurr D. J.; Ghaemmaghami A. M.; Alexander M. R. Single-Cell Metabolic Profiling of Macrophages Using 3D OrbiSIMS: Correlations with Phenotype. Anal. Chem. 2022, 94, 9389 10.1021/acs.analchem.2c01375. 35713879 PMC9260720

[ref20] Kotowska A. M.; Trindade G. F.; Mendes P. M.; Williams P. M.; Aylott J. W.; Shard A. G.; Alexander M. R.; Scurr D. J. Protein identification by 3D OrbiSIMS to facilitate in situ imaging and depth profiling. Nat. Commun. 2020, 11 (1), 5832 10.1038/s41467-020-19445-x. 33203841 PMC7672064

[ref21] Edney M. K.; Kotowska A. M.; Spanu M.; Trindade G. F.; Wilmot E.; Reid J.; Barker J.; Aylott J. W.; Shard A. G.; Alexander M. R.; et al. Molecular Formula Prediction for Chemical Filtering of 3D OrbiSIMS Datasets. Anal. Chem. 2022, 94 (11), 4703–4711. 10.1021/acs.analchem.1c04898. 35276049 PMC8943605

[ref22] Doncheva N. T.; Morris J. H.; Gorodkin J.; Jensen L. J. Cytoscape StringApp: Network Analysis and Visualization of Proteomics Data. J. Proteome Res. 2019, 18 (2), 623–632. 10.1021/acs.jproteome.8b00702. 30450911 PMC6800166

[ref23] aLinke F.; Johnson J. E. C.; Kern S.; Bennett C. D.; Lourdusamy A.; Lea D.; Clifford S. C.; Merry C. L. R.; Stolnik S.; Alexander M. R.; et al. Identifying new biomarkers of aggressive Group 3 and SHH medulloblastoma using 3D hydrogel models, single cell RNA sequencing and 3D OrbiSIMS imaging. Acta Neuropathol. Commun. 2023, 11 (1), 6 10.1186/s40478-022-01496-4. 36631900 PMC9835248

[ref24] García-Morales V.; Montero F.; González-Forero D.; Rodríguez-Bey G.; Gómez-Pérez L.; Medialdea-Wandossell M. J.; Domínguez- VíVíAs G.; García-Verdugo J. M.; Moreno-López B. Membrane-derived phospholipids control synaptic neurotransmission and plasticity. PloS Biol. 2015, 13 (5), e1002153–e1002153 10.1371/journal.pbio.1002153. 25996636 PMC4440815

[ref25] aPereira A. C.; Saroja S. Apolipoprotein E4 drives tau propagation through astrocyte-secreted glypican-4. Alzheimer’s Dement. 2021, 17 (S3), e054243 10.1002/alz.054243.

[ref26] Fitz N. F.; Nam K. N.; Wolfe C. M.; Letronne F.; Playso B. E.; Iordanova B. E.; Kozai T. D. Y.; Biedrzycki R. J.; Kagan V. E.; Tyurina Y. Y.; et al. Phospholipids of APOE lipoproteins activate microglia in an isoform-specific manner in preclinical models of Alzheimer’s disease. Nat. Commun. 2021, 12 (1), 3416 10.1038/s41467-021-23762-0. 34099706 PMC8184801

[ref27] Fayed N.; Modrego P. J.; Rojas-Salinas G.; Aguilar K. Brain glutamate levels are decreased in Alzheimer’s disease: a magnetic resonance spectroscopy study. Am. J. Alzheimer’s Dis Other Dementias 2011, 26 (6), 450–456. 10.1177/1533317511421780. PMC10845671 21921084

[ref28] Schousboe A.; Scafidi S.; Bak L. K.; Waagepetersen H. S.; McKenna M. C. Glutamate metabolism in the brain focusing on astrocytes. Adv. Neurobiol. 2014, 11, 13–30. 10.1007/978-3-319-08894-5_2. 25236722 PMC4667713

[ref29] Xu J.; Begley P.; Church S. J.; Patassini S.; Hollywood K. A.; Jüllig M.; Curtis M. A.; Waldvogel H. J.; Faull R. L. M.; Unwin R. D.; et al. Graded perturbations of metabolism in multiple regions of human brain in Alzheimer’s disease: Snapshot of a pervasive metabolic disorder. Biochim. Biophys. Acta, Mol. Basis Dis. 2016, 1862 (6), 1084–1092. 10.1016/j.bbadis.2016.03.001. PMC4856736 26957286

[ref30] Lau C. G.; Zukin R. S. NMDA receptor trafficking in synaptic plasticity and neuropsychiatric disorders. Nat. Rev. Neurosci. 2007, 8 (6), 413–426. 10.1038/nrn2153. 17514195

[ref31] Bröer S.; Bröer A.; Hansen J. T.; Bubb W. A.; Balcar V. J.; Nasrallah F. A.; Garner B.; Rae C. Alanine metabolism, transport, and cycling in the brain. J. Neurochem. 2007, 102 (6), 1758–1770. 10.1111/j.1471-4159.2007.04654.x. 17504263

[ref32] D’Aniello A.; Vetere A.; Fisher G. H.; Cusano G.; Chavez M.; Petrucelli L. Presence of d-alanine in proteins of normal and Alzheimer human brain. Brain Res. 1992, 592 (1), 44–48. 10.1016/0006-8993(92)91656-Y. 1450921

[ref33] aRaj T.; Li Y. I.; Wong G.; Humphrey J.; Wang M.; Ramdhani S.; Wang Y.-C.; Ng B.; Gupta I.; Haroutunian V.; et al. Integrative transcriptome analyses of the aging brain implicate altered splicing in Alzheimer’s disease susceptibility. Nat. Genet. 2018, 50 (11), 1584–1592. 10.1038/s41588-018-0238-1. 30297968 PMC6354244

[ref34] Mahajan P.; Fiehn O.; Barupal D. IDSL.GOA: Gene Ontology Analysis for interpreting metabolomic datasets. Sci. Rep. 2024, 14, 1299 10.1101/2023.03.25.534225. 38221536 PMC10788336

