# Supplementary material for: Metabolomic and Proteomic Analysis of ApoE4-Carrying H4 Neuroglioma Cells in Alzheimer’s Disease Using OrbiSIMS and LC-MS/MS
Source: Anal Chem. 2024 Jul 11;96(29):11760–70. doi: 10.1021/acs.analchem.4c01201 (PMC11270533; doi:10.1021/acs.analchem.4c01201)
Supplement: Supplementary file 1 — ac4c01201_si_001.pdf [file ac4c01201_si_001.pdf]

Supporting Information

Metabolomic and Proteomic Analysis of ApoE4-Carrying H4 Neuroglioma Cells in Alzheimer's Disease Using OrbiSIMS and LC-MS/MS

Li Lu[1], Anna M. Kotowska[1], Stefanie Kern[1], Min Fang[2], Timothy R. Rudd[2], Morgan R. Alexander[1], David J. Scurr[1]*, Zheying Zhu[1]*

1. School of Pharmacy, The University of Nottingham, University Park Campus, NG7 2RD, UK

2. Medicines and Healthcare products Regulatory Agency (MHRA), Blanche Lane, South Mimms, EN6 3QG UK

Contents

S1. Supplemented Experimental Section 1

S2 OrbiSIMS data analysis of H4 control and ApoE4 KI cells........ 5

Note 1........ 5

S3. LC-MS/MS polar metabolomics data analysis of H4 control and ApoE4 KI cells........ 15

S4. Comparative analysis of LC-MS/MS with OrbiSIMS 22

S5. Peptide assignment by OrbiSIMS 27

S6. Proteomics data analysis and GO analysis 39

S1. Supplemented Experimental Section

Cell culture and ApoE4 knock-in neuroglioma cell by Crispr-cas9

Neuroglioma cells H4 (ATCC® HTB-148TM) were obtained from the American Type Culture Collection (ATCC) and grown at 37°C with 5% CO2 in Dulbecco's Modified Eagle's Medium supplemented with 10% fetal bovine serum and 1% penicillin-streptomycin. The cells are resuspended in a cryopreservation medium (complete culture medium supplemented with 5% DMSO) for cryopreservation. To start with the ApoE4 gene knock-in (KI) process, gRNA is firstly designed according to the target sequence, following the Invitrogen TrueDesign Genome Editor platform that designs gRNA and Donor DNA (https://apps.thermofisher.com/apps/genome-editing-portal/). The gRNA and donor DNA sequences of the ApoE4 gene are shown in Table S1.

The cells are seeded as 4×10^5/well in a 6-well plate before transfection so that they are 50% confluent on the day of transfection. On the day of transfection, the manufacturer's guide is followed for gene editing (Table S2). After transfection, the cells are isolated by trypsin and the concentration of cells is quantified by a hemocytometer. The cell suspension is further diluted at a concentration of 5 cells/mL, and 100µL of the 5 cells/mL suspension is transferred into each well of the 96-well plate and incubated for 7-14 days. Once the cells

reach 80% confluence, the cells are transferred and expanded to 12-well plates and 6-well plates. The expanded cells are used to perform Western blot to screen for lines with the highest ApoE4 expression.

Table S1. The sequences of gRNA and donor DNA for ApoE4 gene

Reagents	Sequences
gRNA	G*G*A*GGACGUGUGCGGCCGCC + modified scaffold
Donor DNA	OECAGGCCCGGCTGGGCGCGGACATGGAGGACGTGCGCGGCCGCCTG GTGCAGTACCGCGGCGAGGTGCAGGCCAZEC

Table S2. The gene-editing protocol and medium used in ApoE4 knock-in experiment. On the day of transfection, prepare Tube 1 and Tube 2 as described in the table, incubate Tube 2 for 1min at RT, then add Tube 2 into Tube1 and mix well by pipetting. After incubating for 10min at RT, the transfection mixture is added to cells and incubated at 37°C, 5% CO2 for 2 days. Finally, the transfected samples are used for further analysis and monoclonal culture.

Tube 1: Reagent	6-well	Tube 2: Reagent	6-well
Opti-MEM™ I Medium	125μL	Opti-MEM™ I Medium	125μL
TrueCut™ Cas9 Protein v2	6.26μL (6250ng/37.5pmol)	Lipofectamine™ CRISPRMAX™ Reagent	7.5μL
gRNA(10μM)	3.75μL (1200ng/37.5pmol)		
Donor DNA	10.2μL (2500ng)		
Lipofectamine™ Cas9 PLUS Reagent	12.5μL		

Protein extraction and Western blot

Proteins in cells were extracted using RIPA lysis buffer containing Halt Protease Inhibitor Cocktail and Halt Phosphatase Inhibitor Cocktail. The protein concentration was determined and normalized to the protein concentration of 2.5μg/μL for each cell lysate, an equal volume of 4X Laemmli sample buffer was added in. Cell lysate in sample buffer was then boiled at 100°C for 5min to reduce and denature proteins in samples. 20μg of total protein of each sample was loaded into each well of SDS-PAGE gel (12%), along with a molecular weight marker. The gel was run for 30min at 80V, followed by 120V for 60min. Proteins in the gel were transferred to the PVDF membrane at 2.5A, 25V for 3min in the Bio-Rad turbo system. The membrane was blocked for 1h at room temperature using blocking buffer (5% BSA). The PVDF membrane was incubated with APOE4 antibody (1:1000 dilution) in blocking buffer overnight at 4°C. After incubating overnight with primary antibody, the membrane was washed three times with TBST for 10min each. The membrane was incubated with secondary antibody in blocking buffer at RT for 1h and then washed three times with TBST for 10 min each. The secondary antibody in membrane was detected by Chemiluminescent solution using GelDoc. Information of the primary and secondary antibodies is as follows: β-actin (sigma, 1:1000), ApoE4 (1:1000), anti-rabbit IgG (A6154 sigma, 1:5000), anti-mouse IgG (A4416 sigma, 1:4000).

DNA isolation and Sanger sequence analysis

Cells are grown in a 6-well plate for 24h before DNA isolation, after aspirating with culture medium and being washed twice by PBS, 1 ml DNAzol Reagent (Invitrogen™, 10503027) is

added into each well. The cells are lysed by gently pipetting after being transferred into new Eppendorf tubes. DNA from cell lysate is precipitated by adding 500µL pure ethanol, mixed by inversion, and stored at room temperature for 3min. DNA quickly becomes visible, then the supernatant is aspirated. DNA precipitate is washed twice with 1 ml of 75% ethanol. DNA is air-dried for 15 seconds after removing ethanol. DNA is then dissolved in 300µL of 8mM NaOH. DNA in NaOH solution is stable for more than one year at -20°C.

The PCR products for sequencing are generated using Q5 High-Fidelity DNA polymerase (NEB, M0491), followed by the manufacturer's guidance. Forward primer for PCR (F-CCTCCCACTGTGCGACACCCTCC) and reverse primer (R-GTCCGGCTGCCCATCTCCTCCAT) were obtained from Invitrogen. PCR product length is ~532bp. PCR products were sent to the DNA sequencing facility of the University of Nottingham for Sanger sequence analysis.

HCS LipidTOX^{TM} green neutral lipid stain

H4 cells were plated at a density of 5×10^5/well in a 6-well plate with 50µg/mL poly-D-lysine coated $1^{\#}$ coverslips. After incubation at 37°C with 5% CO2 for 24h, cells were fixed by 4% formaldehyde for 30min at RT. Cells were washed with PBS buffer 3 times and then incubate with 1× LipidTOX neutral lipid stain for 30min. NucBlue DAPI stain was incubated with cells for 5min before imaging. After cells have been stained with Lipid and DAPI dyes, coverslips were picked up and gently mounted by using Fluoromount^{TM} Aqueous Mounting Medium (Sigma, F4680) on the glass slides. Allow fluoromount to dry for 30-45min at RT, then the stained samples were imaged using Confocal laser scanning microscopy (ZEISS LSM 900 with AiryScan2)from the Nanoscale and Microscale Research Centre of University of Nottingham. For LipidTox staining, an excitation wavelength of 498 nm and an emission wavelength of 507 nm were used. For imaging DAPI, excitation/emission (Ex/Em) wavelengths of 353 nm and 465 nm were utilized.

LC-MS/MS analysis

LC-MS/MS analysis was performed using Q-Exactive Plus mass spectrometer equipped with a Dionex U3000 UHPLC system. Samples for metabolomics (10µL, 4°C) were separated and eluted through a ZIC-pHILIC column (4.6 × 150 mm, 5µm particle size, Merck SeQuant, Darmstadt, Germany) at an HPLC flow rate of 300µL min^{-1} and a temperature of 45□. The gradients started with 20% A (20 mM ammonium carbonate in water) and 80% of B (acetonitrile) and increased to 95% A over 15 min, then the composition was returned to its initial conditions in 2 min, and the column was re-equilibrated for 7 min. The MS instrumentation was set as ESI+ and ESI- switching acquisition modes for profiling samples and in data-dependent MS/MS for identification of QC samples. Details of MS parameters were shown as follows: spray voltage at 4.5 kV (ESI^+) and -3.5 (ESI^-), the capillary voltage at 20 V (ESI^+) and −15 V (ESI^-). The sheath, auxiliary, and sweep gas flow rates were 40, 5, and 1 (arbitrary unit), respectively, for both modes. Capillary and heater temperatures were maintained at 275°C and 150°C, respectively. Data were acquired for LC-MS profiling with a resolution of 70,000 from m/z 70–1050. Top 5 ddMS/MS was performed on the QC sample (n = 3) at a resolution of 17,500 and stepped normalized collision energy (NEC) of 20, 30, and 40.

The cellular samples of the control group and ApoE4 KI group were randomized and analyzed in a single LC-MS analytical run with the mixtures of authentic standards and reagent blanks. Pooled QC (n = 4) was injected at the beginning of the analysis to condition the column and after every 3–5 samples (n = 6) to check the stability, robustness, repeatability, and performance of the analytical system.

LC-MS/MS data analysis

The LC-MS data are pre-processed for untargeted metabolomics, univariate analysis, and metabolite identification by Compound Discoverer 3.3 software (https://mycompounddiscoverer.com/). Furthermore, principal component analysis (PCA) and partial least squares-discriminant analysis (PLS-DA) are processed using SIMCA P. Metabolite identification is performed using Compound Discoverer 3.3 software by matching retention time of detected peaks with the authentic standards, MS/MS fragmentation with mzCloud database and accurate masses with human metabolites in BioCyc database (https://biocyc.org/?sid=biocyc14-3922769627). Retention time ranges from 0.5min to 15min (RT tolerance at 0.5min), the mass range is 70-1050 m/z, and mass tolerance ≤ 5 ppm is used for peak picking and identification. The confidence of identified metabolites is classified into four levels according to the classification rules of the metabolomics standard initiative. Level 1 confidence of metabolite identification is based on accurate mass, retention time, and MS/MS value, Level 2 confidence of metabolite identification is based on accurate mass, retention time, or MS/MS value. After confidence selection, Level 3 confidence of metabolite identification based on accurate mass and all unknown is classified as Level 4.

Data were analyzed using GraphPad Prism and expressed as the mean ± standard deviation (SD). The statistically significant difference of the metabolites between control and ApoE4 KI group was evaluated using Student's t-test and PLS-DA analysis. FDR-adjusted p-value <0.05 combined with VIP>1 was considered as statistical significance.

S2 OrbiSIMS data analysis of H4 control and ApoE4 KI cells.

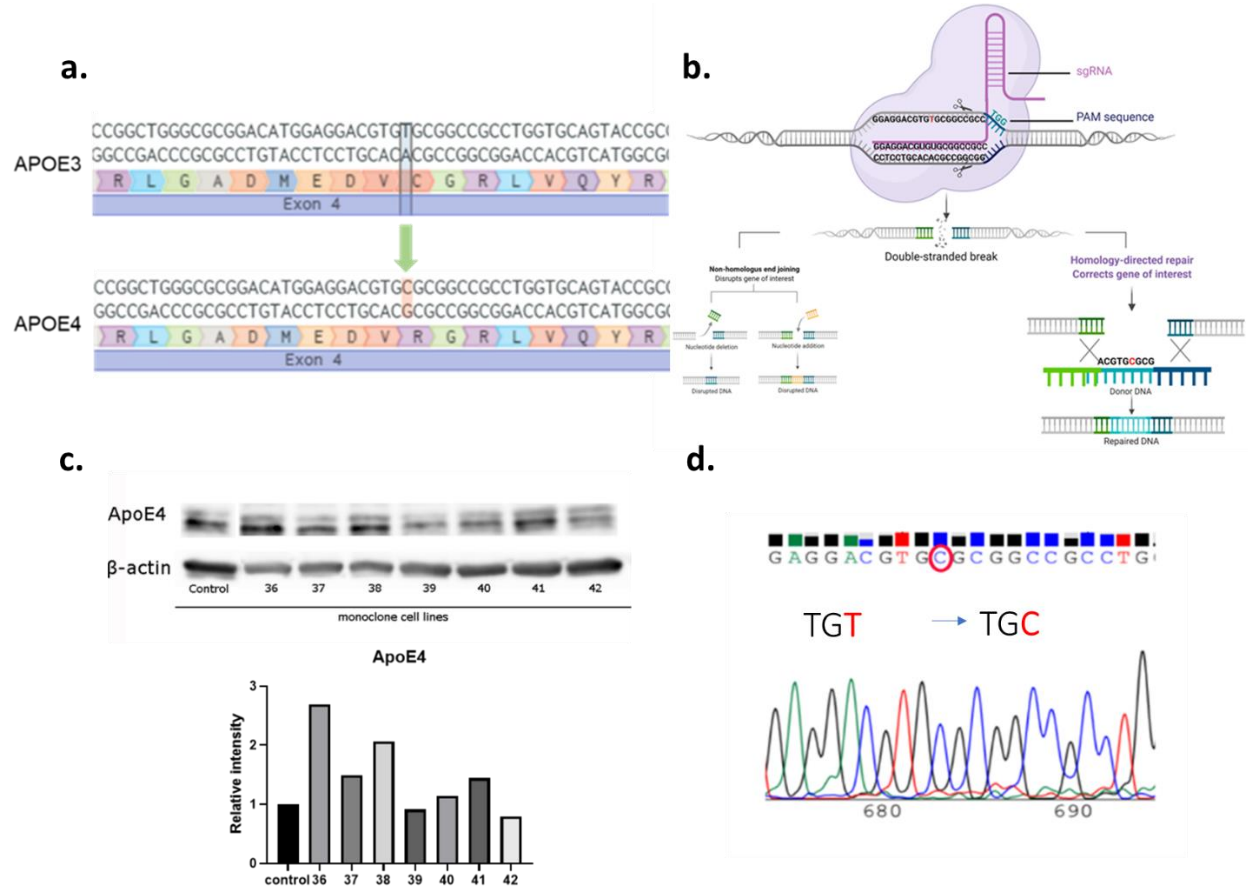

Figure S1. a) The DNA sequences of ApoE3 and ApoE4 variants. b) The gene editing principle of CRISPR-Cas9, in which to mutate ApoE3 into ApoE4 by replacing T to C, replacing cysteine by arginine in rs429358. In terms of the knock-in mechanism, firstly, gRNA is designed according to the target sequence of the gene. Then designed gRNA is combined with Cas9 protein, the gRNA-Cas9 complex was transfected into the cell with a lipofectamine reagent. When DNA is cleavaged by Cas9, two pathways happen, one is non-homologous end joining, which is the pathway of choice when utilizing CRISPR-Cas9 to generate a genetic knockout, the other is the homology-directed repair, in which a donor DNA template that shares homology with the targeted area can induce HDR, resulting in ApoE4 sequence knock-in. c) Cell line 36 has the most significantly ApoE4 over-expression that represents more than 2 times of expression compared with the control group. d) The Sanger sequence proved the successful ApoE4 knock-in into H4 cells. Therefore, cell line 36 was used to further analyze as ApoE4 knock-in group.

Note 1

The phospholipid is an essential component of the cell that modulates membrane stability, transmits cellular signal, and stabilizes synapsis [1]. *De novo* biosynthesis of phospholipids begins with the combination of triglyceride with fatty acid chains, followed by linking lipid head groups such as serine, ethanolamine, glycerol, inositol, and choline to generate phosphatidylserine (PS), phosphatidylethanolamine (PE), phosphatidylglycerol (PG), phosphatidylinositol (PI) and phosphatidylcholine (PC) [2, 3]. Phosphatidic acid (PA) is the vital biosynthetic precursor for forming all of the acylglycerol lipids in the cell. Cyclic phosphatidic acid (CPA) is an analog of a growth factor-like phospholipid mediator, while,

lysophosphatidic acid (LPA) is related to the regulation of the cell cycle, differentiation and survival of neuronal cells. Aside from glycerophospholipid, sphingolipid is a class of lipid-containing core of long-chain amino alcohol and sphingosine, including sphingomyelins and glycosphingolipids, which play important roles in signal transduction and cell recognition. Hexosylceramides (HexCer) serve as key precursors for the biosynthesis of glycosphingolipids which are also a part of the cell membrane. Sphingomyelin (SM) constitutes microdomains of the cell membrane (lipid raft and caveolae) and implicates in the regulation of transmembrane signaling [4], and CerP is a ceramide 1-phosphate that is a derivative of sphingosine. Therefore, disorders of sphingolipid metabolism particularly impact neural tissue.

Table S3. The peak list of annotated metabolites in OrbiSIMS analysis

**Metabolites**	**Classification**	**Deviation (ppm)**	**Accurate peak mass**	**Formula**	**Detected from previous OrbiSIMS studies**
myo-Inositol	Alcohols and polyols	0.5	179.0563	$C_6H_{11}O_6^-$	No
Lactate	Alpha hydroxy acids and derivatives	-2	89.02458	$C_3H_5O_3^-$	Yes
1-Aminocyclopropane-1-carboxylate	Amino acids	-0.2	100.0404	$C_4H_6NO_2^-$	No
Alanine	Amino acids	-2	88.0402	$C_3H_6NO_2^-$	No
Asparagine	Amino acids	-0.1	131.0465	$C_4H_7N_2O_3^-$	Yes
Glutamate	Amino acids	0	146.0461	$C_5H_8NO_4^-$	No
Glutamine	Amino acids	-0.1	145.062	$C_5H_9N_2O_3^-$	Yes
Histidine	Amino acids	0.1	154.0622	$C_6H_8N_3O_2^-$	Yes
Leucine/isoleucine	Amino acids	-0.1	130.0873	$C_6H_{12}NO_2^-$	Yes
Lysine	Amino acids	-0.3	145.0982	$C_6H_{13}N_2O_2^-$	Yes
Ornithine	Amino acids	-0.1	131.0826	$C_5H_{11}N_2O_2^-$	No
Proline	Amino acids	0.2	114.0561	$C_5H_8NO_2^-$	Yes
Taurine	Amino acids	-0.1	124.0076	$C_2H_6NO_3S^-$	No
Tryptophan	Amino acids	0.7	203.0828	$C_{11}H_{11}N_2O_2^-$	Yes
Tyrosine	Amino acids	0.3	180.0668	$C_9H_{10}NO_3^-$	Yes
Valine	Amino acids	0.2	118.0861	$C_5H_{10}NO_2^-$	Yes
Aspartate	Amino acids	1.3	132.0305	$C_4H_6NO_4^-$	Yes
Acetylglycine	Amino acids derivative	0.3	116.0353	$C_4H_6NO_3^-$	No
1-Methyl-histidine	Amino acids derivatives	0	168.0779	$C_7H_{10}N_3O_2^-$	No
C16 taurine	Amino acids derivatives	0.6	362.2373	$C_{18}H_{36}NO_4S^-$	Yes
C18:1 taurine	Amino acids derivatives	-0.5	388.2525	$C_{20}H_{38}NO_4S^-$	Yes
Cysteate	Amino acids derivatives	1.7	167.9974	$C_3H_6NO_5S^-$	No
Glutathione	Amino acids derivatives	0.5	306.0766	$C_{10}H_{16}N_3O_6S^-$	No

Homocysteic acid	Amino acids derivatives	1.8	182.0132	$C_4H_8NO_5S^-$	No
Kynurenine	Amino acids derivatives	0.4	207.0776	$C_{10}H_{11}N_2O_3^-$	No
N-Acetylhistidine	Amino acids derivatives	0.3	196.0728	$C_8H_{10}N_3O_3^-$	No
Phenylalanine	Amino acids derivatives	0.2	164.0719	$C_9H_{10}NO_2^-$	Yes
Pyroglutamic acid	Amino acids derivatives	0.2	128.0356	$C_5H_6NO_3^-$	No
N-Acetylaspartate	Amino acids derivatives	1	174.0409	$C_6H_8NO_5^-$	No
4-Aminobenzoate	Benzenoids	-0.3	136.0404	$C_7H_6NO_2^-$	No
Benzoate	Benzenoids	0.1	121.0295	$C_7H_5O_2^-$	No
Hippurate	Benzenoids	0.4	178.0512	$C_9H_8NO_3^-$	No
Dimethylbenzimidazole	Benzimidazoles	-0.2	145.0771	$C_9H_9N_2^-$	No
Glucose 6-phosphate	Carbohydrates and carbohydrate conjugates	0.6	259.0226	$C_6H_{12}O_9P^-$	No
Glyceraldehyde 3-phosphate	Carbohydrates and carbohydrate conjugates	0.8	168.9909	$C_3H_6O_6P^-$	No
5-phospho-α-D-ribose 1-diphosphate, [M-H-H20]-	Carbohydrates and carbohydrate conjugates	0.7	370.934	$C_5H_{10}P_3O_{13}^-$	No
Glucosamine 6-phosphate, [M-H-H2O]-	Carbohydrates and carbohydrate conjugates	0.6	240.0281	$C_6H_{11}PNO_7^-$	No
X5P/R5P [M-H-H20]-	Carbohydrates and carbohydrate conjugates	0.5	211.0014	$C_5H_8PO_7^-$	No
N-Acetyl-glucosamine 1-phosphate	Carbohydrates and carbohydrate conjugates	0.8	300.0492	$C_8H_{15}NO_9P^-$	No
5-Aminolevulinate	Carboxylic acids and derivatives	-0.1	130.0512	$C_5H_9NO_3^-$	No
Creatine	Carboxylic acids and derivatives	-0.2	130.0622	$C_4H_8N_3O_2^-$	No
Creatinine	Carboxylic acids and derivatives	0.6	112.0517	$C_4H_6N_3O^-$	No
Fumarate	Dicarboxylic acids and derivatives	0.1	115.004	$C_4H_3O_4^-$	No
Succinate	Dicarboxylic acids and derivatives	2	117.0196	$C_4H_5O_4^-$	No
2-Oxobutanoate	Keto acids and derivatives	-0.3	101.0247	$C_4H_6O_3^-$	No
Glycerol 3-phosphate	Lipids	0.3	171.0067	$C_3H_8O_6P^-$	Yes
Itaconate	Lipids	0.2	129.0194	$C_5H_5O_4^-$	No
CAR 13:0;O4	Lipids	0.1	420.2603	$C_{20}H_{38}NO_8^-$	Yes
CerP(21:0)/LPC O-13:1/LPE O-16:1	Lipids	-0.1	436.2833	$C_{21}H_{43}NO_6P^-$	No
Cholesterol Sulfate	Lipids	-0.5	465.3042	$C_{27}H_{45}O_4S^-$	No
CL(78:9) $[M-2H]^{2-}$	Lipids	-1.4	764.5169	$C_{87}H_{150}O_{17}P_2^-$	No

CPA(16:0)/ LPA O-16:2	Lipids	0.5	391.2257	$C_{19}H_{36}O_6P^-$	Yes
CPA(18:0)/LPA O-18:2	Lipids	0.5	419.257	$C_{21}H_{40}O_6P^-$	Yes
CPA(18:1)/LPA O-18:3	Lipids	0.5	417.2414	$C_{21}H_{38}O_6P^-$	Yes
FA(14:0)	Lipids	0.4	227.2019	$C_{14}H_{27}O_2^-$	Yes
FA(15:0)	Lipids	1	241.2175	$C_{15}H_{29}O_2^-$	Yes
FA(16:0)	Lipids	0.9	255.2332	$C_{16}H_{31}O_2^-$	Yes
FA(16:1)	Lipids	0.8	253.2175	$C_{16}H_{29}O_2^-$	Yes
FA(17:0)	Lipids	0.9	269.2488	$C_{17}H_{33}O_2^-$	Yes
FA(17:1)	Lipids	0.8	267.2332	$C_{17}H_{31}O_2^-$	Yes
FA(18:0)	Lipids	0.6	283.2642	$C_{18}H_{35}O_2^-$	Yes
FA(18:1)	Lipids	0.5	281.2486	$C_{18}H_{33}O_2^-$	Yes
FA(18:2)	Lipids	0.4	279.2331	$C_{18}H_{31}O_2^-$	Yes
FA(20:0)	Lipids	0.9	311.2957	$C_{20}H_{40}O_2^-$	No
FA(20:1)	Lipids	0.3	309.28	$C_{20}H_{37}O_2^-$	No
FA(20:2)	Lipids	0.3	307.2643	$C_{20}H_{35}O_2^-$	No
FA 20:3/ST 20:0;O2	Lipids	0.3	305.2487	$C_{20}H_{33}O_2^-$	Yes
FA 20:4/ST 20:1;O2	Lipids	0.3	303.233	$C_{20}H_{31}O_2^-$	Yes
FA 20:5/ST 20:2;O2'	Lipids	0	301.2173	$C_{20}H_{29}O_2^-$	Yes
FA(22:0)	Lipids	-0.8	339.3266	$C_{22}H_{43}O_2^-$	Yes
FA(22:1)	Lipids	0	337.3112	$C_{22}H_{41}O_2^-$	No
FA(22:2)	Lipids	0	335.2956	$C_{22}H_{39}O_2^-$	No
FA 22:3/ST 22:0;O2	Lipids	0	333.2799	$C_{22}H_{37}O_2^-$	Yes
FA 22:4/ST 22:1;O2	Lipids	0.4	331.2644	$C_{22}H_{35}O_2^-$	Yes
FA 22:5/ST 22:2;O2	Lipids	0.2	329.2487	$C_{22}H_{33}O_2^-$	Yes
FA 22:6/ST 22:3;O2	Lipids	0	327.2329	$C_{22}H_{31}O_2^-$	No
FA(24:0)	Lipids	-0.6	367.3579	$C_{24}H_{47}O_2^-$	No
FA(24:1)	Lipids	-0.3	365.3424	$C_{24}H_{45}O_2^-$	No
PA head group	Lipids	0	152.9958	$C_3H_6O_5P^-$	Yes
Hex2Cer30:1;O4	Lipids	0.1	836.5378	$C_{42}H_{78}NO_{15}^-$	No
Hex2Cer32:1;O4	Lipids	0.1	864.5691	$C_{44}H_{82}NO_{15}^-$	No
Hex2Cer32:2;O4	Lipids	0.1	862.5534	$C_{44}H_{80}NO_{15}^-$	No
Hex2Cer34:4;O4	Lipids	0	886.5534	$C_{46}H_{80}NO_{15}^-$	No
HexCer32:1;O4	Lipids	0.4	702.5165	$C_{38}H_{72}NO_{10}^-$	Yes
LdMePE(16:0)/LPC 15:0/LPC O-15:1;O/LPE 18:0/LPE O-18:1;O	Lipids	0.2	480.3097	$C_{23}H_{47}O_7NP^-$	No
LPA(16:0) /LPA O-16:1;O	Lipids	0.3	409.2362	$C_{19}H_{38}O_7P^-$	No
LPA(18:0) /LPA O-18:1;O	Lipids	0.1	437.2674	$C_{21}H_{42}O_7P^-$	No
LPE(P-18:0)/LPC O-15:1/LPE O-18:1	Lipids	0	464.3147	$C_{23}H_{47}O_6NP^-$	No
LPI(16:0)/LPI O-16:1;O	Lipids	0.5	571.2891	$C_{25}H_{48}O_{12}P^-$	No
LPI(18:0)/LPI O-18:1;O	Lipids	0.6	599.3205	$C_{27}H_{52}O_{12}P^-$	No

Oleamide/SPB 18:2;O	Lipids	0.5	280.2646	$C_{18}H_{34}NO^-$	No
LPA 32:2;O/LPG O-29:3/PA 32:1/PA O-32:2;O	Lipids	0	645.45	$C_{35}H_{66}O_8P^-$	No
LPA 34:2;O/LPG O-31:3/PA 34:1/PA O-34:2;O	Lipids	0.4	673.4816	$C_{37}H_{70}O_8P^-$	Yes
LPA 34:3;O/LPG O-31:4/PA 34:2/PA O-34:3;O/DG 41:11/TG O-41:11	Lipids	1.3	671.4666	$C_{37}H_{68}O_8P^-$	No
LPG O-33:3/PA 36:1/PA O-36:2;O	Lipids	0.6	701.5131	$C_{39}H_{74}O_8P^-$	Yes
LPG O-33:4/PA 36:2/PA O-36:3;O	Lipids	0.4	699.4973	$C_{39}H_{72}O_8P^-$	Yes
LPA 32:1;O/LPG O-29:2/PA 32:0/PA O-32:1;O/DG 39:9/DG O-39:10;O/TG O-39:9	Lipids	0.7	647.4662	$C_{35}H_{68}O_8P^-$	Yes
PA 38:1/PA O-38:2;O	Lipids	0.6	729.5444	$C_{41}H_{78}O_8P^-$	Yes
PA 38:3/PA O-38:4;O/DG 45:12/TG O-45:12	Lipids	1.3	725.5134	$C_{41}H_{74}O_8P^-$	Yes
PA 38:4/PA O-38:5;O	Lipids	-0.7	723.4965	$C_{41}H_{72}O_8P^-$	Yes
CerP 39:1;O4/LPC 31:1;O/LPE 34:1;O/LPS O-33:1/PC 31:0/PC O-31:1;O/PE 34:0/PE O-34:1;O	Lipids	0.7	718.5398	$C_{39}H_{77}NO_8P^-$	Yes
CerP 39:2;O4/LPC 31:2;O/LPE 34:2;O/LPS O-33:2/PC 31:1/PC O-31:2;O/PE 34:1/PE O-34:2;O	Lipids	0.4	716.5239	$C_{39}H_{75}NO_8P^-$	Yes
CerP 41:2;O4/LPC 33:2;O/PC 33:1/PC O-33:2;O/PE 36:1/PE O-36:2;O	Lipids	0.8	744.5555	$C_{41}H_{79}NO_8P^-$	Yes
CerP 41:3;O4/LPC 33:3;O/PC 33:2/PC O-33:3;O/PE 36:2/PE O-36:3;O	Lipids	1.7	742.5405	$C_{41}H_{77}NO_8P^-$	Yes
CerP 42:2;O4/LPC 34:2;O/PC 34:1/PC O-34:2;O/PE 37:1/PE O-37:2;O	Lipids	-2	758.569	$C_{42}H_{81}NO_8P^-$	No
CerP 42:3;O4/LPC 34:3;O/PC 34:2/PC O-34:3;O/PE 37:2/PE O-37:3;O	Lipids	1.3	756.5559	$C_{42}H_{79}NO_8P^-$	No
PE(38:3)/HexCer 37:3;O4	Lipids	13	768.5649	$C_{43}H_{79}NO_8P^-$	No
CerP 43:5;O4/PC 35:4/PC O-35:5;O/PE 38:4/PE O-38:5;O	Lipids	2.2	766.5409	$C_{43}H_{77}NO_8P^-$	Yes
PC 37:7/PC O-37:8;O/PE 40:7/PE O-40:8;O	Lipids	-0.6	788.5231	$C_{45}H_{75}NO_8P^-$	No

PC 39:9/PC O-39:10;O/PE 42:9/PE O-42:10;O	Lipids	-0.6	812.5231	$C_{47}H_{75}NO_8P^-$	No
CerP 39:2;O3/LPC 31:2/LPC O-31:3;O/LPE 34:2/LPE O-34:3;O/PC O-31:2/PE O-34:2/ PE(P-34:1)	Lipids	0.5	700.529	$C_{39}H_{75}NO_7P^-$	Yes
PE(P-36:1)	Lipids	3.4	728.5624	$C_{41}H_{79}NO_7P^-$	Yes
CerP 43:3;O3/PC O-35:3/PE O-38:3/ PE(P-38:2)	Lipids	1.8	754.577	$C_{43}H_{81}NO_7P^-$	Yes
CerP 43:6;O3/PC O-35:6/PE O-38:6/ PE(P-38:5)	Lipids	2.1	748.5303	$C_{43}H_{75}NO_7P^-$	Yes
PE(P-40:6)/HexCer 39:6;O3	Lipids	9.3	774.5515	$C_{45}H_{77}NO_7P^-$	Yes
BMP 34:1/LPG 34:2;O/PG 34:1/PG O-34:2;O	Lipids	0.3	747.5184	$C_{40}H_{76}O_{10}P^-$	No
DG 46:10;O2/TG 46:9;O/TG O-46:10;O2/PG 36:1/PG O-36:2;O	Lipids	1.5	775.5507	$C_{42}H_{80}O_{10}P^-$	No
PG 36:2/PG O-36:3;O	Lipids	0.5	773.5342	$C_{42}H_{78}O_{10}P^-$	No
Phosphocholine-CH3	Lipids	-0.1	168.0431	$C_4H_{11}NO_4P^-$	Yes
PI head group	Lipids	0.8	241.0121	$C_6H_{10}PO_8^-$	Yes
LPI 32:1;O/PI 32:0/PI O-32:1;O	Lipids	0.4	809.5188	$C_{41}H_{78}O_{13}P^-$	No
LPI 32:2;O/PI 32:1/PI O-32:2;O	Lipids	-0.4	807.5026	$C_{41}H_{76}O_{13}P^-$	No
LPI 34:2;O/PI 34:1/PI O-34:2;O	Lipids	0.1	835.5343	$C_{43}H_{80}O_{13}P^-$	Yes
LPI 34:3;O/PI 34:2/PI O-34:3;O	Lipids	-0.2	833.5184	$C_{43}H_{78}O_{13}P^-$	No
PI 35:1/PI O-35:2;O	Lipids	0.2	849.55	$C_{44}H_{82}O_{13}P^-$	No
PI 35:2/PI O-35:3;O	Lipids	0.2	847.5344	$C_{44}H_{80}O_{13}P^-$	No
PI 36:1/PI O-36:2;O	Lipids	0.3	863.5657	$C_{45}H_{84}O_{13}P^-$	Yes
PI 36:2/PI O-36:3;O	Lipids	0.3	861.5501	$C_{45}H_{82}O_{13}P^-$	Yes
PI 36:3/PI O-36:4;O	Lipids	0.8	859.5349	$C_{45}H_{80}O_{13}P^-$	No
PI 36:4/PI O-36:5;O	Lipids	0.7	857.5191	$C_{45}H_{78}O_{13}P^-$	Yes
PI 37:1/PI O-37:2;O	Lipids	1.5	877.5825	$C_{46}H_{86}O_{13}P^-$	No
PI 37:2/PI O-37:3;O	Lipids	1.2	875.5666	$C_{46}H_{84}O_{13}P^-$	No
PI 37:3/PI O-37:4;O	Lipids	-0.7	873.5492	$C_{46}H_{82}O_{13}P^-$	No
PI 37:4/PI O-37:5;O	Lipids	-1.7	871.5327	$C_{46}H_{80}O_{13}P^-$	No
PI 38:1/PI O-38:2;O	Lipids	1.5	891.5981	$C_{47}H_{88}O_{13}P^-$	No
PI 38:2/PI O-38:3;O	Lipids	0.2	889.5813	$C_{47}H_{86}O_{13}P^-$	No
PI 38:3/PI O-38:4;O	Lipids	0.4	887.5659	$C_{47}H_{84}O_{13}P^-$	Yes
PI 38:4/PI O-38:5;O	Lipids	0.2	885.5501	$C_{47}H_{82}O_{13}P^-$	Yes
PI 38:5/PI O-38:6;O	Lipids	0.2	883.5343	$C_{47}H_{80}O_{13}P^-$	No
PI 38:6/PI O-38:7;O	Lipids	-0.7	881.5179	$C_{47}H_{78}O_{13}P^-$	No

PI 39:3/PI O-39:4;O	Lipids	0.2	901.5813	$C_{48}H\_{86}O_{13}P^-$	No
PI 39:4/PI O-39:5;O	Lipids	0.3	899.5658	$C_{48}H_{84}O_{13}P^-$	No
PI 40:2/PI O-40:3;O	Lipids	1.4	917.6137	$C_{49}H_{90}O_{13}P^-$	No
PI 40:3/PI O-40:4;O	Lipids	0.3	915.5971	$C_{49}H_{88}O_{13}P^-$	No
PI 40:4/PI O-40:5;O	Lipids	0.5	913.5816	$C_{49}H_{86}O_{13}P^-$	No
PI 40:5/PI O-40:6;O	Lipids	0.7	911.5661	$C_{49}H_{84}O_{13}P^-$	Yes
PI 40:6/PI O-40:7;O	Lipids	0.1	909.5499	$C_{49}H_{82}O_{13}P^-$	No
PI 40:7/PI O-40:8;O	Lipids	0.5	907.5347	$C_{49}H_{80}O_{13}P^-$	No
PI(P-36:0)/PI O-36:1/TG 49:9;O3	Lipids	0.9	849.587	$C_{45}H_{86}O_{12}P^-$	No
PI(P-36:3)/PI O-36:4/TG 49:12;O3	Lipids	2.3	843.5413	$C_{45}H_{80}O_{12}P^-$	No
POV-PG/BMP 21:1;O/LPI O-18:2/PG 21:1;O	Lipids	0.4	581.3099	$C_{27}H_{50}O_{11}P^-$	Yes
CerP 40:3;O6/LPS 34:2;O/PS 34:1/PS O-34:2;O	Lipids	0.1	760.5135	$C_{40}H_{75}NO_{10}P^-$	No
CerP 41:3;O6/PS 35:1/PS O-35:2;O	Lipids	-1.3	774.5281	$C_{41}H_{77}NO_{10}P^-$	No
CerP 42:3;O6/PS 36:1/PS O-36:2;O	Lipids	0	788.5447	$C_{42}H_{79}NO_{10}P^-$	Yes
CerP 42:4;O6/PS 36:2/PS O-36:3;O	Lipids	0.9	786.5297	$C_{42}H_{77}NO_{10}P^-$	Yes
PS(39:3)	Lipids	5.3	826.5647	$C_{45}H_{81}NO_{10}P^-$	No
CerP 42:3;O5/PC 34:2;O/PE 37:2;O/PS O-36:2/PS(P-36:1)	Lipids	-1.1	772.5489	$C_{42}H_{79}NO_9P^-$	No
CerP 44:5;O5/PC 36:4;O/PE 39:4;O/PS O-38:4/ PS(P-38:3)	Lipids	0.1	796.5499	$C_{44}H_{79}NO_9P^-$	No
PE-Cer 36:1;O2/SM 33:1;O2	Lipids	0.4	687.5449	$C_{38}H_{76}N_2O_6P^-$	Yes
Dopamine	Neurotransmitters	0	152.0717	$C_8H_{10}NO_2^-$	Yes
GABA	Neurotransmitters	0	102.0563	$C_4H_8NO_2^-$	Yes
Serotonin	Neurotransmitters	0.1	175.0877	$C_{10}H_{11}N_2O^-$	Yes
Acetylphosphate	Organic phosphoric acids and derivatives	0	138.9802	$C_2H_4O_5P^-$	No
L-Histidinol	Organonitrogen compounds	0	140.0829	$C_6H_{10}N_3O^-$	No
Phenylpyruvic acid	Phenylpyruvic acid derivatives	-0.1	163.0401	$C_9H_7O_3^-$	No
Adenosine	Purine nucleosides	1.7	266.0894	$C_{10}H_{12}N_5O_4^-$	No
Guanosine	Purine nucleosides	0.7	282.0846	$C_{10}H_{12}N_5O_5^-$	No
ADP	Purine nucleotides	0.8	426.0225	$C_{10}H_{14}N_5O_{10}P_2^-$	No
AMP	Purine nucleotides	0.3	346.0559	$C_{10}H_{13}N_5O_7P^-$	Yes
cAMP	Purine nucleotides	0.7	328.046	$C_{10}H_{11}N_5O_6P^-$	Yes
GMP Guanosine	Purine	0.7	362.051	$C_{10}H_{13}N_5O_8P^-$	Yes

monophosphate	nucleotides				
Adenine	Purines and purine derivatives	0.2	134.0472	$C_5H_4N_5^-$	Yes
Guanine	Purines and purine derivatives	-0.2	150.0421	$C_5H_4N_5O^-$	Yes
Hypoxanthine	Purines and purine derivatives	0.2	135.0313	$C_5H_3N_4O^-$	Yes
Purine	Purines and purine derivatives	0	119.0363	$C_5H_3N_4^-$	No
xanthine	Purines and purine derivatives	-0.1	151.0261	$C_5H_3N_4O_2^-$	No
6-Methylnicotinamide	Pyridines and derivatives	0.2	135.0564	$C_7H_7N_2O^-$	No
Pyridoxine	Pyridines and derivatives	-0.1	168.0666	$C_8H_{10}NO_3^-$	No
Pyridoxal	Pyridines and derivatives	0	166.051	$C_8H_8NO_3^-$	No
Nicotinamide	Pyridines and derivatives	0.1	121.0407	$C_6H_5N_2O^-$	No
Cytidine	Pyrimidine nucleosides	1.7	242.0787	$C_9H_{12}N_3O_5^-$	No
Uridine	Pyrimidine nucleosides	1.4	243.0626	$C_9H_{11}N_2O_6^-$	No
Cytidine monophosphate	Pyrimidine nucleotides	0.1	322.0446	$C_9H_{13}N_3O_8P^-$	No
dCMP	Pyrimidine nucleotides	0.6	306.0498	$C_9H_{13}N_3O_7P^-$	No
dUMP	Pyrimidine nucleotides	1.5	307.0341	$C_9H_{12}N_2O_8P^-$	No
UDP-N-acetylglucosamine	Pyrimidine nucleotides	0.2	606.0748	$C_{17}H_{26}N_3O_{17}P_2^-$	No
Uridine 5'-monophosphate	Pyrimidine nucleotides	0.1	323.0286	$C_9H_{12}N_2O_9P^-$	No
Cytosine	Pyrimidines and pyrimidine derivatives	0.4	110.036	$C_4H_4N_3O^-$	Yes
Methylcytosine	Pyrimidines and pyrimidine derivatives	-0.2	124.0516	$C_5H_6N_3O^-$	Yes
Thymine	Pyrimidines and pyrimidine derivatives	-0.1	125.0356	$C_5H_5N_2O_2^-$	Yes
Uracil	Pyrimidines and pyrimidine derivatives	0.5	111.0203	$C_4H_3N_2O_2^-$	Yes

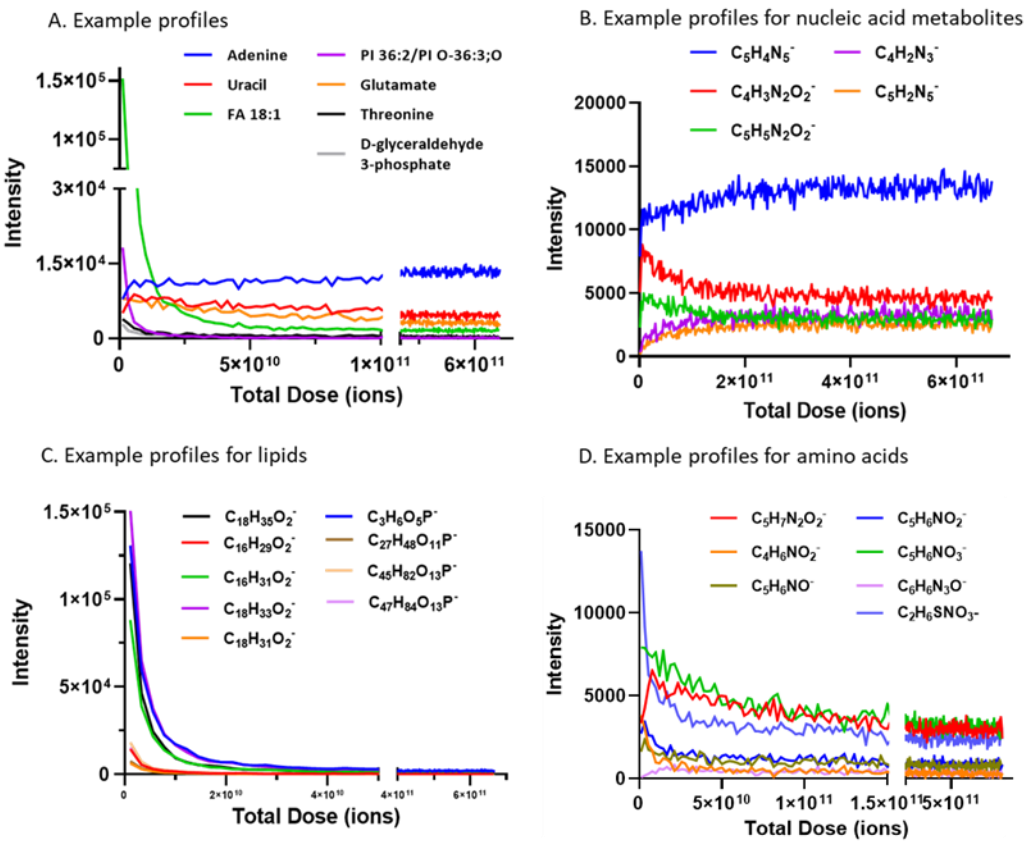

Figure S2. The example profiles of metabolites assigned from H4 neuroglioma cells by using OrbiSIMS. It shows the unique depth profile of each compound class, such as lipids, the major components of membrane, which are highly enriched in the surface of cells.

Table S4. The peak list of significant features in OrbiSIMS

Feature	Accurate mass	Formula	FDR	VIP
Alanine	90.05489	$C_3H_7NO_2$	0.001168	1.0242
Succinate	117.01962	$C_4H_6O_4$	0.001368	1.1278
Valine	118.08605	$C_5H_{11}NO_2$	0.000765	1.0213
Taurine	124.00758	$C_2H_7NO_3S$	0.00597	1.0231
Methylcytosine	124.0516	$C_5H_6N_3O^-$	0.0008	1.0228
Aspartate	132.03047	$C_4H_7NO_4$	0.005173	1.1645
Creatine	132.07656	$C_4H_9N_3O_2$	0.002327	1.1287
L-Histidinol	140.0829	$C_6H_{10}N_3O^-$	0.001411	1.1308
Glutamate	146.04608	$C_5H_9NO_4$	0.002434	1.1162
LPA 32:2;O/LPG O-29:3/PA 32:1/PA O-32:2;O	645.45	$C_{35}H_{66}O_8P$-	0.009797	1.3917
LPA 34:2;O/LPG O-31:3/PA 34:1/PA O-34:2;O	673.4816	$C_{37}H_{70}O_8P$-	0.021659	1.0075
PE-Cer 36:1;O2/SM 33:1;O2	687.5449	$C_{38}H_{76}N_2O_6P^-$	0.000994	1.2395
CerP 39:2;O4/LPC 31:2;O/LPE	716.5239	$C_{39}H_{75}NO_8P^-$	0.006462	1.1123

34:2;O/LPS O-33:2/PC 31:1/PC O-31:2;O/PE 34:1/PE O-34:2;O				
BMP 34:1/LPG 34:2;O/PG 34:1/PG O-34:2;O	747.5184	$C_{40}H_{76}O_{10}P^-$	0.006266	1.0287
CerP 43:6;O3/PC O-35:6/PE O-38:6/ PE(P-38:5)	748.5303	$C_{43}H_{75}NO_{7}P^-$	0.029789	1.4563
CerP 43:3;O3/PC O-35:3/PE O-38:3/ PE(P-38:2)	754.577	$C_{43}H_{81}NO_{7}P^-$	0.002326	1.5094
CerP 42:3;O4/LPC 34:3;O/PC 34:2/PC O-34:3;O/PE 37:2/PE O-37:3;O	756.5559	$C_{42}H_{79}O_{8}NP^-$	0.001956	1.6763
CerP 42:2;O4/LPC 34:2;O/PC 34:1/PC O-34:2;O/PE 37:1/PE O-37:2;O	758.569	$C_{42}H_{81}O_{8}NP^-$	0.009101	1.5345
CerP 40:3;O6/LPS 34:2;O/PS 34:1/PS O-34:2;O	760.5135	$C_{40}H_{75}O_{10}NP^-$	0.006746	1.1989
CL(78:9) $[M-2H]^{2-}$	764.5169	$C_{87}H_{150}O_{17}P_{2}^-$	0.018211	2.149
CerP 43:5;O4/PC 35:4/PC O-35:5;O/PE 38:4/PE O-38:5;O	766.5409	$C_{43}H_{77}NO_{8}P^-$	0.004197	2.1336
PE(38:3)/HexCer 37:3;O4	768.5649	$C_{43}H_{79}O_{8}NP^-$	0.003316	1.5664
CerP 42:3;O5/PC 34:2;O/PE 37:2;O/PS O-36:2/PS(P-36:1)	772.5489	$C_{42}H_{79}O_{9}NP^-$	0.003612	1.716
CerP 41:3;O6/PS 35:1/PS O-35:2;O	774.5281	$C_{41}H_{77}O_{10}NP^-$	0.011201	1.7778
PE(P-40:6)/HexCer 39:6;O3	774.5515	$C_{45}H_{77}O_{7}NP^-$	0.001559	1.4745
DG 46:10;O2/TG 46:9;O/TG O-46:10;O2/PG 36:1/PG O-36:2;O	775.5507	$C_{42}H_{8}0O_{10}P^-$	0.049386	1.2227
CerP 42:4;O6/PS 36:2/PS O-36:3;O	786.5297	$C_{42}H_{77}NO_{10}P^-$	0.008293	1.2742
PC 37:7/PC O-37:8;O/PE 40:7/PE O-40:8;O	788.5231	$C_{45}H_{75}O_{8}NP^-$	0.005007	1.6742
PC 39:9/PC O-39:10;O/PE 42:9/PE O-42:10;O	812.5231	$C_{47}H_{75}NO_{8}P^-$	0.010054	1.7771

PI 36:3/PI O-36:4;O	859.5349	$C_{45}H_{80}O_{13}P^-$	0.022609	1.0812
PI 37:3/PI O-37:4;O	873.5492	$C_{46}H_{82}O_{13}P^-$	0.006905	1.2638
PI 38:3/PI O-38:4;O	887.5659	$C_{47}H_{84}O_{13}P^-$	0.005864	1.0319
PI 38:2/PI O-38:3;O	889.5813	$C_{47}H_{86}O_{13}P^-$	0.039471	1.1953
PI 39:4/PI O-39:5;O	899.5658	$C_{48}H_{84}O_{13}P^-$	0.00597	1.0172
PI 39:3/PI O-39:4;O	901.5813	$C_{48}H_{86}O_{13}P^-$	0.03287	1.0457

S3. LC-MS/MS polar metabolomics data analysis of H4 control and ApoE4 KI cells.

The HILIC LC-MS/MS methodology, especially for extracting polar metabolites, includes extracting intracellular metabolic pools using methanol and separating molecules using the HILIC LC condition (gradients LC eluted system by using mobile phase A (20 mM ammonium carbonate in water) and phase B (acetonitrile)). ESI ionization was used in LC-MS/MS analysis, the soft ionization of ESI makes little fragmentation of analyte molecules (Figure S3a). PCA analysis includes QC samples used to evaluate analytical performance, and the similarity of ion number detected in each group also shows the good performance of the LC-MS/MS method (Figure S4a and S4b).

Table S5. The classification of annotated metabolites detected from H4 cells by LC-MS/MS.

**Classification of metabolites**	**Number of metabolites (121)**
Lipids	22
Amino acids and AA derivatives	25
Carbohydrates and carbohydrate conjugates	10
Carboxylic/Dicarboxylic acids and derivatives	20
Purines nucleotides and purine derivatives	6
Pyridines and derivatives	4
Pyrimidines and pyrimidine derivatives	5
Nucleosides, nucleotides, and analogues	4
Neurotransmitters	1
Other organic compounds	24

From the data acquired, 1095 peaks have been detected, which were used for metabolites identification by using the levels of confidence suggested by the metabolomics standard

initiative. After confidence selection, 121 metabolites were identified with high confidence in identification (Levels 1 and 2) based on MSI levels of identification (Table S5, S6), including amino acids and derivatives, lipids (fatty acids), carboxylic/dicarboxylic acids, carbohydrates, purines, pyridines, pyrimidines, and other organic acids. The level of alanine, aspartate and glutamate decreased under the presence of ApoE4 which is consistent with clinical metabolomics alteration (Figure S3b).

The score plot of the PLS-DA analysis shows that the metabolic profile of the control is significantly separated from that of the ApoE4 KI group (Figure S4b). Moreover, 40 metabolites were considered statistically significant with the condition of VIP > 1 and FDR value < 0.05 (Table S7). The log normalized intensity of these 40 features is shown in Figures S3c and S3d. Pathway analysis based on 40 features presented in Figure S3d indicates that the metabolism of taurine and hypotaurine is the most affected pathway by LC-MS/MS. Metabolites (cysteate, taurine, hypotaurine) involved in this pathway are down-regulated in ApoE4-expressing cells. Taurine has a neuromodulator role in brain development, and one of its intermediates is hypotaurine [5]. Moreover, cysteate is an amino acid generated by the oxidation of cysteine, which is employed in regulating major endogenous antioxidant molecules [6]. Overall, ApoE4 might impair neuronal function and trigger the production of oxidant molecules by interrupting taurine and hypotaurine metabolism.

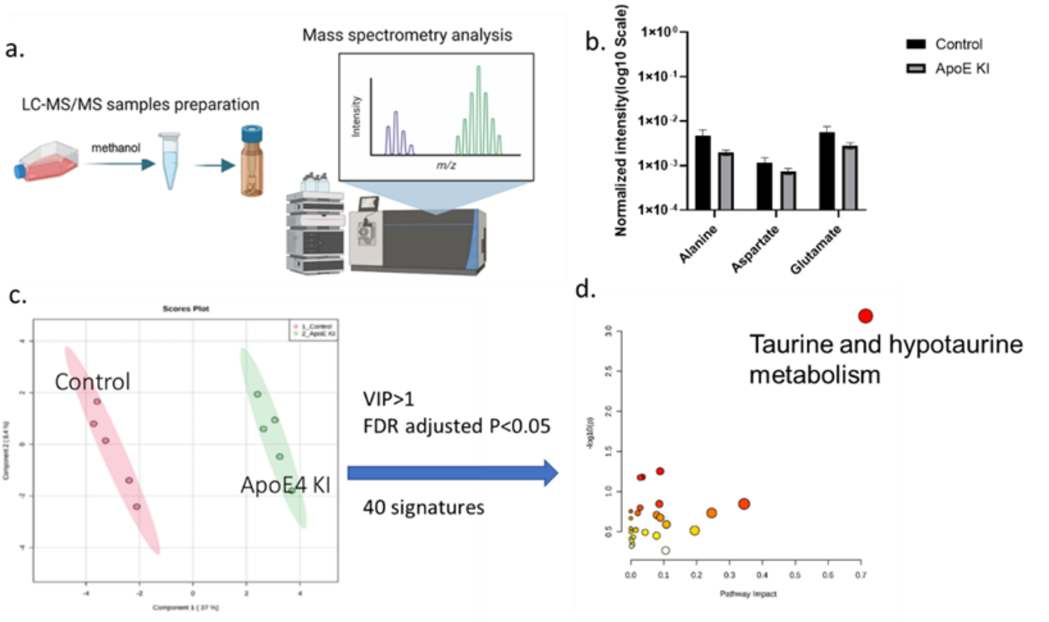

Figure S3. (a) The brief workflow of LC-MS/MS analysis of H4 neuroglioma cells. (b) The normalized intensity of alanine, aspartate, and glutamate observed from Control and ApoE4 KI groups. (c) The PLS-DA score plots for the Control and ApoE4 KI cells. (d) The pathway analysis of 40 signatures by LC-MS/MS.

Table S6. The peak list of identified metabolites (Level 1, 2) in LC-MS analysis

Metabolites 1	Classification	Accurate mass	Formula
myo-Inositol	Alcohols and polyols	179.0563	$C_6H_{12}O_6$
Lactate	Alpha hydroxy acids and derivatives	89.02458	$C_3H_6O_3$
1-Aminocyclopropane-1-carboxylate	Amino acids	84.04431	$C_4H_7NO_2$
Alanine	Amino acids	90.05489	$C_3H_7NO_2$

Asparagine	Amino acids	131.04652	$C_4H_8N_2O_3$
Aspartate	Amino acids	132.03047	$C_4H_7NO_4$
Glutamate	Amino acids	146.04608	$C_5H_9NO_4$
Glutamine	Amino acids	145.06203	$C_5H_{10}N_2O_3$
Histidine	Amino acids	156.07667	$C_6H_9N_3O_2$
Lysine	Amino acids	147.11269	$C_6H_{14}N_2O_2$
Proline	Amino acids	116.07044	$C_5H_9NO_2$
Taurine	Amino acids	124.00758	$C_2H_7NO_3S$
Tryptophan	Amino acids	203.08281	$C_{11}H_{12}N_2O_2$
Tyrosine	Amino acids	180.06684	$C_9H_{11}NO_3$
Valine	Amino acids	118.08605	$C_5H_{11}NO_2$
Arginine	Amino acids	175.11883	$C_6H_{14}N_4O_2$
Glycine	Amino acids	76.03928	$C_2H_5NO_2$
Leucine	Amino acids	132.10172	$C_6H_{13}NO_2$
Serine	Amino acids	106.0498	$C_3H_7NO_3$
Threonine	Amino acids	120.06541	$C_4H_9NO_3$
1-Methyl-histidine	Amino acids derivatives	170.09217	$C_7H_{11}N_3O_2$
Cysteate	Amino acids derivatives	167.99743	$C_3H_7NO_5S$
Glutathione	Amino acids derivatives	306.07657	$C_{10}H_{17}N_3O_6S$
Kynurenine	Amino acids derivatives	207.07762	$C_{10}H_{12}N_2O_3$
N-Acetylaspartate	Amino acids derivatives	174.04089	$C_6H_9NO_5$
Phenylalanine	Amino acids derivatives	164.07185	$C_9H_{11}NO_2$
Pyroglutamic acid	Amino acids derivatives	128.03556	$C_5H_7NO_3$
4-Aminobenzoate	Benzenoids	138.05484	$C_7H_7NO_2$
Benzoate	Benzenoids	123.04388	$C_7H_6O_2$
Hippurate	Benzenoids	178.05115	$C_9H_9NO_3$
2-Ethylhexyl phthalate	Benzenoids	279.15866	$C_{16}H_{22}O_4$
homovanillate	Benzenoids	181.05082	$C_9H_{10}O_4$
N-(2-hydroxyphenyl)acetamide	Benzenoids	152.0705	$C_8H_9NO_2$
Erythrose 4-phosphate	Carbohydrates and carbohydrate conjugates	199.0015	$C_4H_9O_7P$
Fructose 6-phosphate	Carbohydrates and carbohydrate conjugates	259.02242	$C_6H_{13}O_9P$
Gluconic acid	Carbohydrates and carbohydrate conjugates	195.05112	$C_6H_{12}O_7$
Glucono 1,4-lactone	Carbohydrates and carbohydrate conjugates	214.1797	$C_{12}H_{20}O_2$
Glycerate	Carbohydrates and carbohydrate conjugates	105.0196	$C_3H_6O_4$
L-Threonic acid	Carbohydrates and carbohydrate conjugates	135.03021	$C_4H_8O_5$
Mannitol	Carbohydrates and carbohydrate conjugates	181.07155	$C_6H_{14}O_6$
N-Acetyl-D-glucosamine	Carbohydrates and carbohydrate conjugates	222.09686	$C_8H_{15}NO_6$
N-Acetylneuraminate	Carbohydrates and carbohydrate conjugates	308.09875	$C_{11}H_{19}NO_9$

Sucrose	Carbohydrates and carbohydrate conjugates	360.14926	$C_{12}H_{22}O_{11}$
5-Aminolevulinate	Carboxylic acids and derivatives	130.05117	$C_5H_9NO_3$
Creatine	Carboxylic acids and derivatives	132.07656	$C_4H_9N_3O_2$
Creatinine	Carboxylic acids and derivatives	114.06606	$C_4H_7N_3O$
2-Aminoadipate	Carboxylic acids and derivatives	162.07581	$C_6H_{11}NO_4$
Acetylcysteine	Carboxylic acids and derivatives	162.02328	$C_5H_9NO_3S$
Capryloylglycine	Carboxylic acids and derivatives	202.1434	$C_{10}H_{19}NO_3$
Citrate	Carboxylic acids and derivatives	191.01992	$C_6H_8O_7$
Cystathionine	Carboxylic acids and derivatives	223.07439	$C_7H_{14}N_2O_4S$
Glutarate	Carboxylic acids and derivatives	131.0352	$C_5H_8O_4$
Glutathione disulfide	Carboxylic acids and derivatives	613.15822	$C_{20}H_{32}N_6O_{12}S_2$
Guanidinoacetate	Carboxylic acids and derivatives	118.06102	$C_3H_7N_3O_2$
Methionine	Carboxylic acids and derivatives	148.04399	$C_5H_{11}NO_2S$
N-Acetylglutamine	Carboxylic acids and derivatives	189.08669	$C_7H_{12}N_2O_4$
N-Acetyl-L-glutamate	Carboxylic acids and derivatives	190.07075	C_7H_{11} NO_5
N-Acetyl-L-methionine	Carboxylic acids and derivatives	190.0545	$C_7H_{13}NO_3S$
N-Acetylornithine	Carboxylic acids and derivatives	175.10754	$C_7H_{14}N_2O_3$
N-Methyl-lysine	Carboxylic acids and derivatives	161.12835	$C_7H_{16}N_2O_2$
Phenylacetylglycine	Carboxylic acids and derivatives	192.06677	$C_{10}H_{11}NO_3$
Fumarate	Dicarboxylic acids and derivatives	115.00399	$C_4H_4O_4$
Succinate	Dicarboxylic acids and derivatives	117.01962	$C_4H_6O_4$
S-Adenosyl-L-homocysteine	Gamma butyrolactones	385.128	$C_{14}H_{20}N_6O_5S$
Malate	Hydroxy acids and derivatives	133.01447	$C_4H_6O_5$
4-Coumarate	Hydroxycinnamic acids and derivatives	182.08114	$C_9H_8O_3$
5-Hydroxyindoleacetate	Indoles and derivatives	209.09175	$C_{10}H_9NO_3$
2-Oxobutanoate	Keto acids and derivatives	101.02468	$C_4H_6O_3$
Glycerol 3-phosphate	Lipids	171.00669	$C_3H_9O_6P$
Oleamide	Lipids	280.26456	$C_{18}H_{35}NO$
(5Z)-3-aminonon-5-enoic acid	Lipids	170.11886	$C_9H_{17}NO_2$
3-Hydroxy-3-methylglutaric acid	Lipids	161.04575	$C_6H_{10}O_5$
4-Trimethylammoniobutanoate	Lipids	146.11736	$C_7H_{15}NO_2$
Caprylic acid	Lipids	143.10792	$C_8H_{16}O_2$

Decanoic acid	Lipids	171.13924	$C_{10}H_{20}O_2$
Ethyl myristate	Lipids	255.23302	$C_{16}H_{32}O_2$
FA(14:0), Myristic acid	Lipids	227.20186	$C_{14}H_{28}O_2$
FA(18:0), Octadecanoic acid	Lipids	283.2642	$C_{18}H_{36}O_2$
FA(18:1), Oleic acid	Lipids	281.24863	$C_{18}H_{34}O_2$
FA(20:0), Arachidic acid	Lipids	311.29566	$C_{20}H_{40}O_2$
Glycero-3-Phosphocholine	Lipids	258.10959	$C_8H_{20}NO_6P$
Heptanoic acid	Lipids	129.09238	$C_7H_{14}O_2$
Hexadecanamide	Lipids	256.26287	$C_{16}H_{33}NO$
Hexanoic acid	Lipids	115.07669	$C_6H_{12}O_2$
Hexanoylcarnitine	Lipids	260.18501	$C_{13}H_{25}NO_4$
Leucinic acid	Lipids	131.07158	$C_6H_{12}O_3$
Nonanoic acid	Lipids	157.12354	$C_9H_{18}O_2$
O-Acetylcarnitine	Lipids	204.12277	$C_9H_{17}NO_4$
O-Butanoylcarnitine	Lipids	232.15388	$C_{11}H_{21}NO_4$
Propionylcarnitine	Lipids	218.1383	$C_{10}H_{19}NO_4$
GABA	Neurotransmitters	102.05631	$C_4H_9NO_2$
Inosine	Nucleosides, nucleotides, and analogues	269.08739	$C_{10}H_{12}N_4O_5$
Methylthioadenosine	Nucleosides, nucleotides, and analogues	298.09609	$C_{11}H_{15}N_5O_3S$
NAD+	Nucleosides, nucleotides, and analogues	664.11513	$C_{21}H_{27}N_7O_{14}P_2$
NADH	Nucleosides, nucleotides, and analogues	666.13093	$C_{21}H_{29}N_7O_{14}P_2$
2-Hydroxyglutarate	Organic acids and derivatives	147.03014	$C_5H_8O_5$
2-Oxoglutarate	Organic acids and derivatives	145.01451	$C_5H_6O_5$
3-Methyl-2-oxobutanoic acid	Organic acids and derivatives	115.04032	$C_5H_8O_3$
Hypotaurine	Organic acids and derivatives	110.02693	$C_2H_7NO_2S$
Phenolsulfonphthalein	Organic acids and derivatives	355.06255	$C_{19}H_{14}O_5S$
Carnitine	Organic nitrogen compounds	162.11223	$C_7H_{15}NO_3$
Choline	Organic nitrogen compounds	104.10679	$C_5H_{13}NO$
Choline phosphate	Organic nitrogen compounds	184.07308	$C_5H_{14}NO_4P$
Acetoin	Organic oxygen compounds	87.04533	$C_4H_8O_2$
Pantothenate	Organic oxygen compounds	220.11751	$C_9H_{17}NO_5$
methylglyoxal	Organooxygen compounds	71.0139	$C_3H_4O_2$
Adenosine	Purine nucleosides	268.10332	$C_{10}H_{13}N_5O_4$
cAMP	Purine nucleotides	328.04599	$C_{10}H_{12}N_5O_6P$
dGMP	Purine nucleotides	346.0554	$C_{10}H_{14}N_5O_7P$
Adenine	Purines and purine derivatives	136.0616	$C_5H_5N_5$

Hypoxanthine	Purines and purine derivatives	137.04562	$C_5H_4N_4O$
Xanthine	Purines and purine derivatives	151.02634	$C_5H_4N_4O_2$
6-Methylnicotinamide	Pyridines and derivatives	137.0708	$C_7H_8N_2O$
Pyridoxal	Pyridines and derivatives	168.06533	$C_8H_9NO_3$
Pyridoxine	Pyridines and derivatives	170.08091	$C_8H_{11}NO_3$
Nicotinamide	Pyridines and derivatives	123.05507	$C_6H_6N_2O$
UDP-N-acetylglucosamine	Pyrimidine nucleotides	606.07479	$C_{17}H_{27}N_3O_{17}P_2$
UDP-Glucose	Pyrimidine nucleotides	565.04819	$C_{15}H_{24}N_2O_{17}P_2$
Cytosine	Pyrimidines and pyrimidine derivatives	112.05039	$C_4H_5N_3O$
Uracil	Pyrimidines and pyrimidine derivatives	111.02033	$C_4H_4N_2O_2$
Thiamine	Pyrimidines and pyrimidine derivatives	265.11129	$C_{12}H_{16}N_4OS$

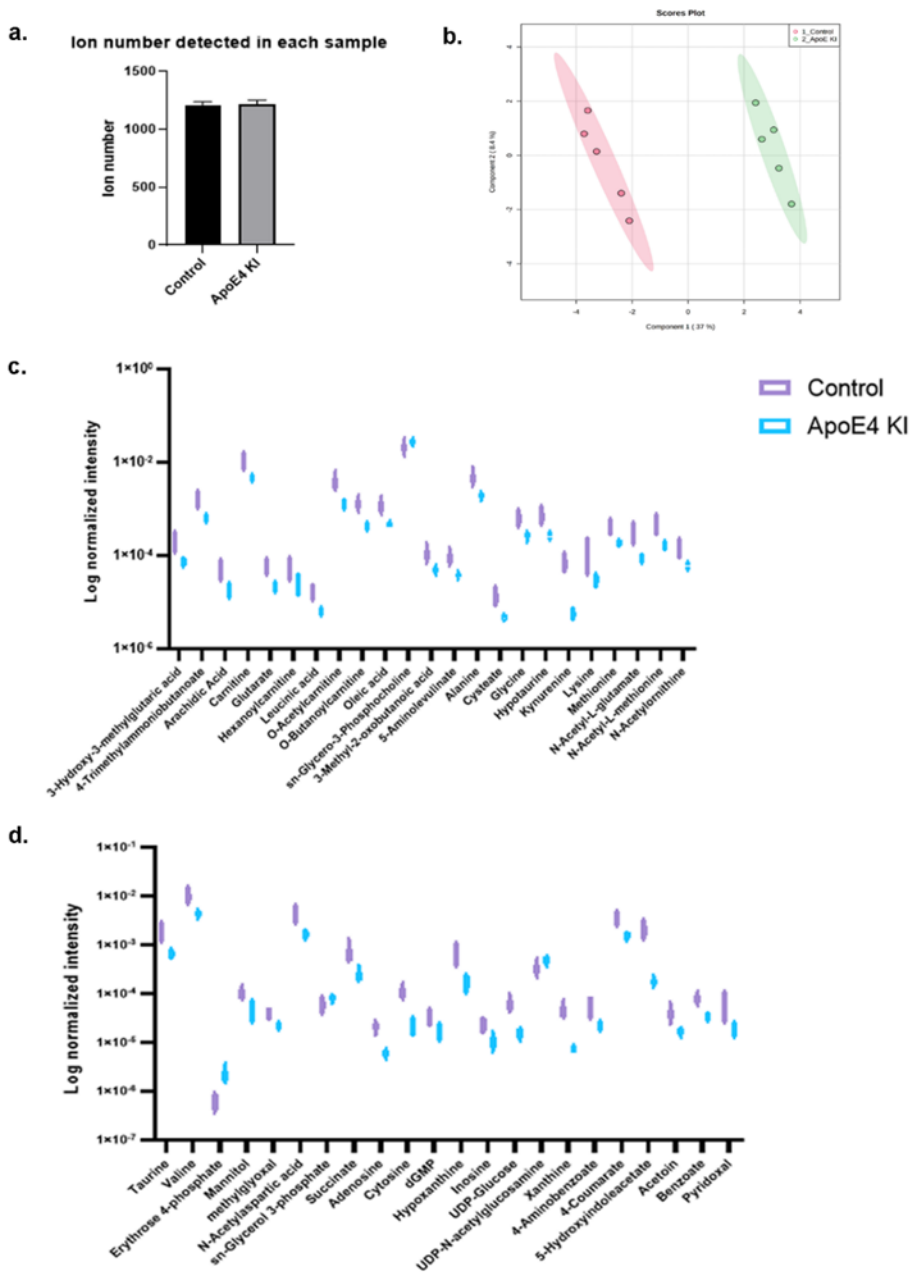

Figure S4. (a) The total ion (feature) numbers were detected in each group by LC-MS/MS. (b) The PLS-DA score plots for control and ApoE4 KI cells. (c)&(d) The relative normalized intensity of significantly identified metabolites detected by LC-MS/MS.

Table S7. The peak list of significant features detected by LC-MS/MS

**Metabolites**	**Accurate mass**	**Formula**	**FDR**	**VIP**
Glycine	76.03928	$C_2H_5NO_2$	0.0019342	1.0322
Acetoin	87.04533	$C_4H_8O_2$	0.0010709	1.031
Alanine	90.05489	$C_3H_7NO_2$	0.000306	1.1256
Hypotaurine	110.02693	$C_2H_7NO_2S$	1.63E-05	1.2865
Cytosine	112.05039	$C_4H_5N_3O$	2.11E-05	2.2366
Nicotinamide	123.05507	$C_6H_6N_2O$	0.0008376	1.2782
Taurine	124.00758	$C_2H_7NO_3S$	3.54E-06	1.3482
5-Aminolevulinate	130.05117	$C_5H_9NO_3$	0.0001145	1.1411
Glutarate	131.0352	$C_5H_8O_4$	0.0018339	1.3432

Leucinic acid	131.07158	$C_6H_{12}O_3$	0.0025993	1.118
Adenine	136.0616	$C_5H_5N_5$	0.028771	1.0925
Hypoxanthine	137.04562	$C_5H_4N_4O$	0.028771	1.5604
4-Aminobenzoate	138.05484	$C_7H_7NO_2$	0.0159	1.2599
4-Trimethylammoniobutanoate	146.11736	$C_7H_{15}NO_2$	1.90E-05	1.1487
Xanthine	151.02634	$C_5H_4N_4O_2$	1.46E-06	2.469
3-Hydroxy-3-methylglutaric acid	161.04575	$C_6H_{10}O_5$	0.0020058	1.1621
N-Methyl-lysine	161.12835	$C_7H_{16}N_2O_2$	0.0094808	1.2952
Cysteate	167.99743	$C_3H_7NO_5S$	0.0001012	1.3467
Pyridoxine	170.08091	$C_8H_{11}NO_3$	0.049896	1.0912
Glycerol 3-phosphate	171.00669	$C_3H_9O_6P$	2.48E-08	2.2516
N-Acetylaspartate	174.04089	$C_6H_9NO_5$	6.31E-06	1.3018
Hippurate	178.05115	$C_9H_9NO_3$	0.037362	1.3491
N-Acetyl-L-methionine	190.0545	$C_7H_{13}NO_3S$	7.08E-06	1.4342
N-Acetyl-L-glutamate	190.07075	$C_7H_{11}NO_5$	6.76E-06	1.7313
Erythrose 4-phosphate	199.0015	$C_4H_9O_7P$	0.000306	2.9675
O-Acetylcarnitine	204.12277	$C_9H_{17}NO_4$	4.97E-05	1.5328
Kynurenine	207.07762	$C_{10}H_{12}N_2O_3$	9.02E-10	3.1993
5-Hydroxyindoleacetate	209.09175	$C_{10}H_9NO_3$	1.61E-10	3.1377
Propionylcarnitine	218.1383	$C_{10}H_{19}NO_4$	0.0058255	2.4719
Pantothenate	220.11751	$C_9H_{17}NO_5$	0.018678	1.3718
O-Butanoylcarnitine	232.15388	$C_{11}H_{21}NO_4$	1.77E-06	1.6527
Glycero-3-Phosphocholine	258.10959	$C_8H_{20}NO_6P$	3.53E-09	2.1667
Fructose 6-phosphate	259.02242	$C_6H_{13}O_9P$	0.032232	1.5376
Hexanoylcarnitine	260.18501	$C_{13}H_{25}NO_4$	0.032008	1.5369
Adenosine	268.10332	$C_{10}H_{13}N_5O_4$	3.65E-05	1.8456
FA(18:1), Oleic acid	281.24863	$C_{18}H_{34}O_2$	0.0025592	1.2298
Methylthioadenosine	298.09609	$C_{11}H_{15}N_5O_3S$	0.047189	1.0305
Glutathione	306.07657	$C_{10}H_{17}N_3O_6S$	0.0001693	1.0561
UDP-Glucose	565.04819	$C_{15}H_{24}N_2O_{17}P_2$	8.05E-05	1.9115
UDP-N-acetylglucosamine	606.07479	$C_{17}H_{27}N_3O_{17}P_2$	1.43E-07	2.3304

S4. Comparative analysis of LC-MS/MS with OrbiSIMS

To evaluate the screening performance of OrbiSIMS in cellular metabolomics, we compared OrbiSIMS data with LC-MS data to exploit how many metabolites were commonly detected in the two methods. The comparative analysis of OrbiSIMS and LC-MS was based on all annotated metabolites. Firstly, the Venn Diagram in Figure 4 shows 50 metabolites commonly detected in both methods, mainly including major amino acids and amino acid derivatives. Moreover, the relative abundance of these molecules in LC-MS and OrbiSIMS are presented in Figures S8, respectively. Almost all features show the same trend between the control and ApoE4 KI groups. While there are six features that did not retain the same change between the two methodologies, including four amino acids and AA derivatives (green circled, alanine, glutamate, taurine, and aspartate), one lipid (glycerol 3-phosphate) and one carboxylic/dicarboxylic acid (fumarate). These six features (m/z <150) not only play a role in energy metabolism and signal transduction, but also as metabolic intermediates. Therefore, they share a similar structure with other higher molecular weight analytes, resulting in the annotated peaks might contain the area of free analytes and fragments that are produced from

other metabolites. In addition, the multiple amino acid fragments detected in OrbiSIMS (Table S7 & S8) also affected the ability of relative quantitative metabolomics.

In conclusion, OrbiSIMS data show a high matching pattern with LC-MS data, suggesting that OrbiSIMS can be used as a metabolomics-screen tool for quickly mapping the metabolic profile of ApoE4-expressing cells. Subsequently, LC-MS is required to further confirm the alteration of the signature obtained from OrbiSIMS analysis, especially for metabolites with lower molecular weight and might be produced from other analytes such as amino acids.

a.

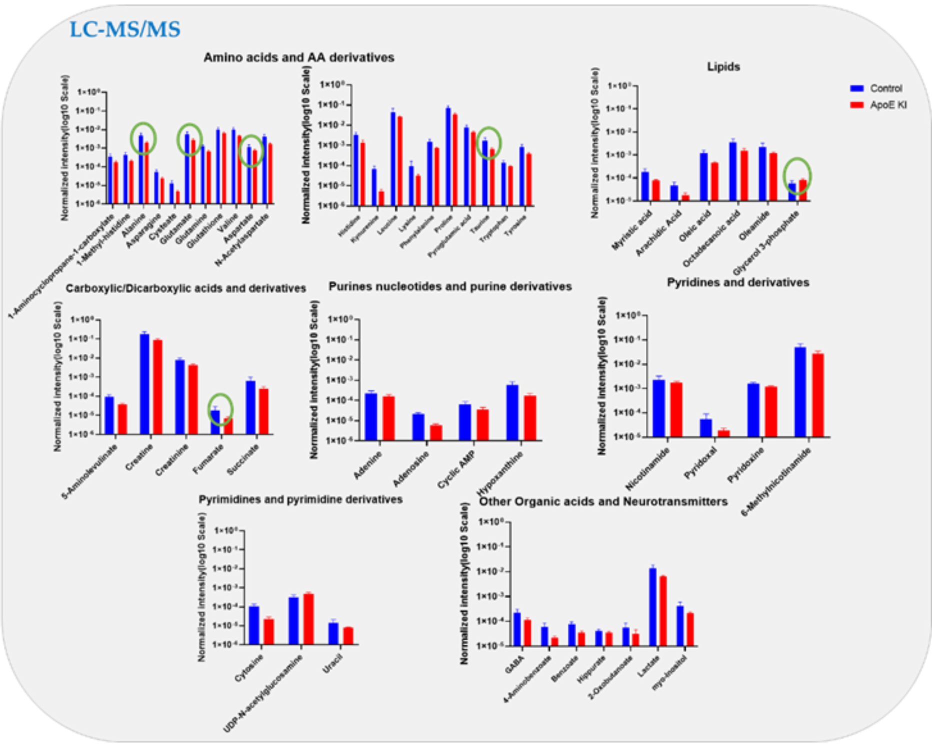

b.

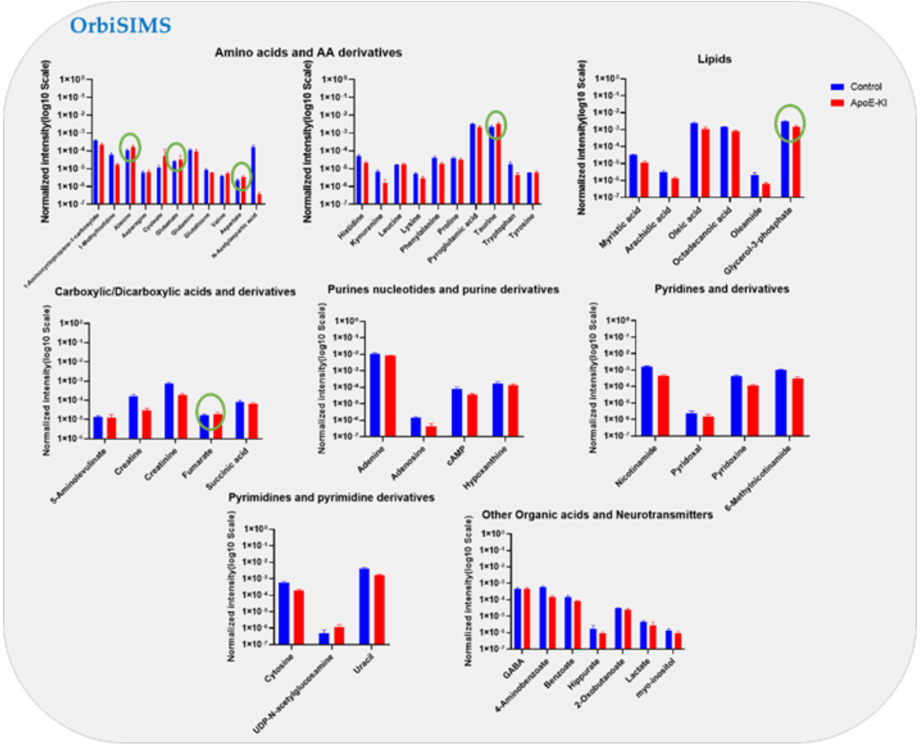

Figure S5. (a) The bar chart shows the relative intensity of 50 metabolites commonly detected in the

LC-MS method between control group and ApoE4 KI group. (b) The bar chart shows the relative intensity of 50 metabolites commonly detected in the OrbiSIMS method between control group and ApoE4 KI group. All of these data were normalized by total ion intensity and scaled by log10 in the bar chart. The green circled metabolites indicate the five features that did not retain the same change between the LC-MS and OrbiSIMS methods.

Table S8. Molecular ions and fragments of amino acids in OrbiSIMS spectra.

**m/z**	**Assignment**	**Amino acids**	**AA Abbreviation**	**Deviation/ppm in H4 control_1 sample**
88.0401	$C_3H_6NO_2^-$	Alanine, Aspartate	A, D	-2
86.0245	$C_3H_4NO_2^-$	Alanine, Cysteine, Hydroxyproline, Leucine, Lysine	A, C, O, L, K	-2.4
131.0825	$C_5H_{11}N_2O_2^-$	Arginine	R	-0.1
156.0777	$C_6H_{10}N_3O_2^-$	Arginine	R	0.3
158.0933	$C_6H_{12}N_3O_2^-$	Arginine	R	0.4
113.0357	$C_4H_5N_2O_2^-$	Asparagine	N	0.6
114.0196	$C_4H_4NO_3^-$	Asparagine	N	0.2
131.0461	$C_4H_7N_2O_3^-$	Asparagine	N	-0.1
115.0037	$C_4H_3O_4^-$	Aspartate	D	0.1
117.0192	$C_4H_5O_4^-$	Aspartate	D	-1.4
132.0304	$C_4H_6NO_4^-$	Aspartate	D	1.3
98.0246	$C_4H_4NO_2^-$	Aspartate, Methionine	D, M	-0.4
102.9859	$C_3H_3SO_2^-$	Cysteine	C	0.7
102.056	$C_4H_8NO_2^-$	Glutamate	E	0
146.0458	$C_5H_8NO_4^-$	Glutamate [M-H]-	E	0
128.0353	$C_5H_6NO_3^-$	Glutamate, Glutamine, Hydroxyproline	E, Q, O	0.2
100.0403	$C_4H_6NO_2^-$	Glutamate, Isoleucine, Methionine, Valine	E, I, M, V	-0.2
85.0292	$C_4H_5O_2^-$	Glutamate, Valine	E, V	-2.7
99.0563	$C_4H_7N_2O^-$	Glutamine	Q	-0.2
145.0617	$C_5H_9N_2O_3^-$	Glutamine [M-H]-	Q	-0.1
82.0295	$C_4H_4NO^-$	Glutamine,	Q, O	-3

		Hydroxyproline		
80.0375	$C_4H_4N_2^-$	Histidine	H	-3.6
81.0454	$C_4H_5N_2^-$	Histidine	H	-3.3
93.0457	$C_5H_5N_2^-$	Histidine	H	-1
104.0254	$C_5H_2N_3^-$	Histidine	H	0.2
108.0567	$C_5H_6N_3^-$	Histidine	H	0.5
137.0355	$C_6H_5N_2O_2^-$	Histidine	H	-0.2
154.0621	$C_6H_8N_3O_2^-$	Histidine	H	0.1
99.0087	$C_4H_3O_3^-$	Hydroxyproline	O	-0.2
110.0247	$C_5H_4NO_2^-$	Hydroxyproline	O	0.4
130.0509	$C_5H_8NO_3^-$	Hydroxyproline	O	-0.1
128.0717	$C_6H_{10}NO_2^-$	Isoleucine, Leucine, Lysine	I, L, K	0.2
130.0872	$C_6H_{12}NO_2^-$	Isoleucine, Leucine, Lysine	I, L, K	-0.1
114.056	$C_5H_8NO_2^-$	Isoleucine, Leucine, Proline	I, L, P	0.2
145.0982	$C_6H_{13}N_2O_2^-$	Lysine	K	-0.3
91.0551	$C_7H_7^-$	Phenylalanine	F	-1.4
97.0083	C_8H^-	Phenylalanine	F	-0.3
103.0553	$C_8H_7^-$	Phenylalanine	F	1.2
118.0662	$C_8H_8N^-$	Phenylalanine	F	0.5
147.0451	$C_9H_7O_2^-$	Phenylalanine	F	0.1
164.0716	$C_9H_{10}NO_2^-$	Phenylalanine	F	0.2
112.0404	$C_5H_6NO_2^-$	Proline	P	0.6
87.0085	$C_3H_3O_3^-$	Serine	S	-2.3
104.0353	$C_3H_6NO_3^-$	Serine	S	1
118.0509	$C_4H_8NO_3^-$	Threonine	T	-0.3
108.0455	$C_6H_6NO^-$	Tryptophan	W	0.5
116.0506	$C_8H_6N^-$	Tryptophan	W	0.3
136.0532	$C_8H_8O_2^-$	Tryptophan	W	2.4
142.0661	$C_{10}H_8N^-$	Tryptophan	W	-0.3
157.0769	$C_{10}H_9N_2^-$	Tryptophan	W	-0.2
203.0825	$C_{11}H_{11}N_2O_2^-$	Tryptophan	W	0.7
93.0344	$C_6H_5O^-$	Tyrosine	Y	-1
119.0502	$C_8H_7O^-$	Tyrosine	Y	0
134.061	$C_8H_8NO^-$	Tyrosine	Y	0

163.04	$C_9H_7O_3^-$	Tyrosine	Y	-0.1
180.0665	$C_9H_{10}NO_3^-$	Tyrosine	Y	0.3
116.0717	$C_5H_{10}NO_2^-$	Valine	V	0.2

Table S9. Fragments of amino acids in peptide chains.

**m/z**	**Assignment**	**Amino acids**	**AA Abbreviation**	**Deviation/ppm in H4 control_1 sample**
82.0295	$C_4H_4NO^-$	Glutamine	Q	-3.0
82.0659	$C_5H_8N^-$	Lysine	K	-3.0
84.0817	$C_5H_{10}N^-$	Leucine, Isoleucine	L, I	-2.8
85.0404	$C_3H_5N_2O^-$	Asparagine	N	-2.5
86.0245	$C_3H_4NO_2^-$	Aspartate	D	-2.4
99.0563	$C_4H_7N_2O^-$	Glutamine	Q	-0.2
100.0403	$C_4H_6NO_2^-$	Glutamate	E	-0.2
108.0567	$C_5H_6N_3^-$	Histidine	H	0.5
110.0723	$C_5H_8N_3^-$	Arginine	R	0.4
118.0662	$C_8H_8N^-$	Phenylalanine	F	0.5
124.0403	$C_6H_6NO_2^-$	Proline	P	-0.2
124.0767	$C_7H_{10}NO^-$	Lysine	K	-0.1
127.0512	$C_5H_7N_2O_2^-$	Glutamine	Q	0.1
127.0876	$C_6H_{11}N_2O^-$	Lysine	K	0.1
134.061	$C_8H_8NO^-$	Tyrosine	Y	0
136.0515	$C_6H_6N_3O^-$	Histidine	H	-0.3
157.0769	$C_{10}H_9N_2^-$	Tryptophan	W	-0.2

S5. Peptide assignment by OrbiSIMS

Peptide fragments assignment by using simsMFP

After performing peak search on each raw data with minimal counts 5000, ion peak list of each sample are generated. Next, simsMFP is performed to filter the ions based on elemental restriction: (C_{4-100}, H_{8-200}, N_{0-20}, O_{0-20}, S_{0-1}) [7]. Then DBE value ($0.1667C_n<DBE<0.6667C_n$) and N/C, O/C, H/C ratio are performed according to Figure S9 that based on the 16 example proteins by Kotowska et al [8]. Finally, peptide fragments assignment from each of H4 samples are listed in Table S10.

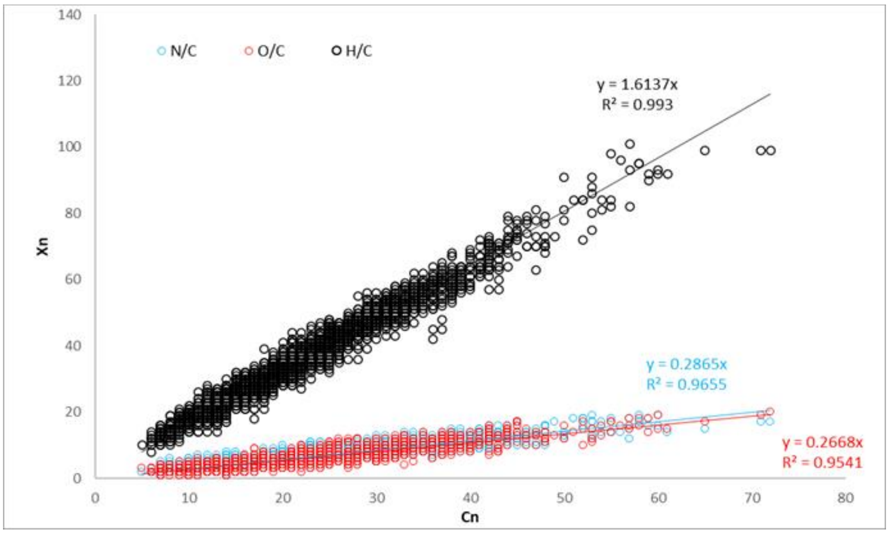

Figure S6. N, O, and H to C ratio of peptide-related ion peaks based on the 16 example proteins.

After applying all restrictions, more than 100 peptide assignments were found in each cell sample (Table S9) that are listed in Table S10 and S11. Venn diagram showed 90 peptide assignments commonly observed in the Control group, and 95 in the ApoE4 KI group. Venn analysis between 90 peptide fragments of Control and 95 of ApoE4 KI further revealed that 52 fragments are detected both from Control and ApoE4 KI. Moreover, we found the relative abundance of those 52 peptide assignments in ApoE4-carried cells much lower than those in control cells. This was suggested that the protein biosynthesis might be inhibited by ApoE4. The defective protein synthesis of neurons by ApoE4 has been reported by Sarayu et al. which indicates an NMDA-mediated alteration of synaptic signalling in ApoE4 treatment [9].

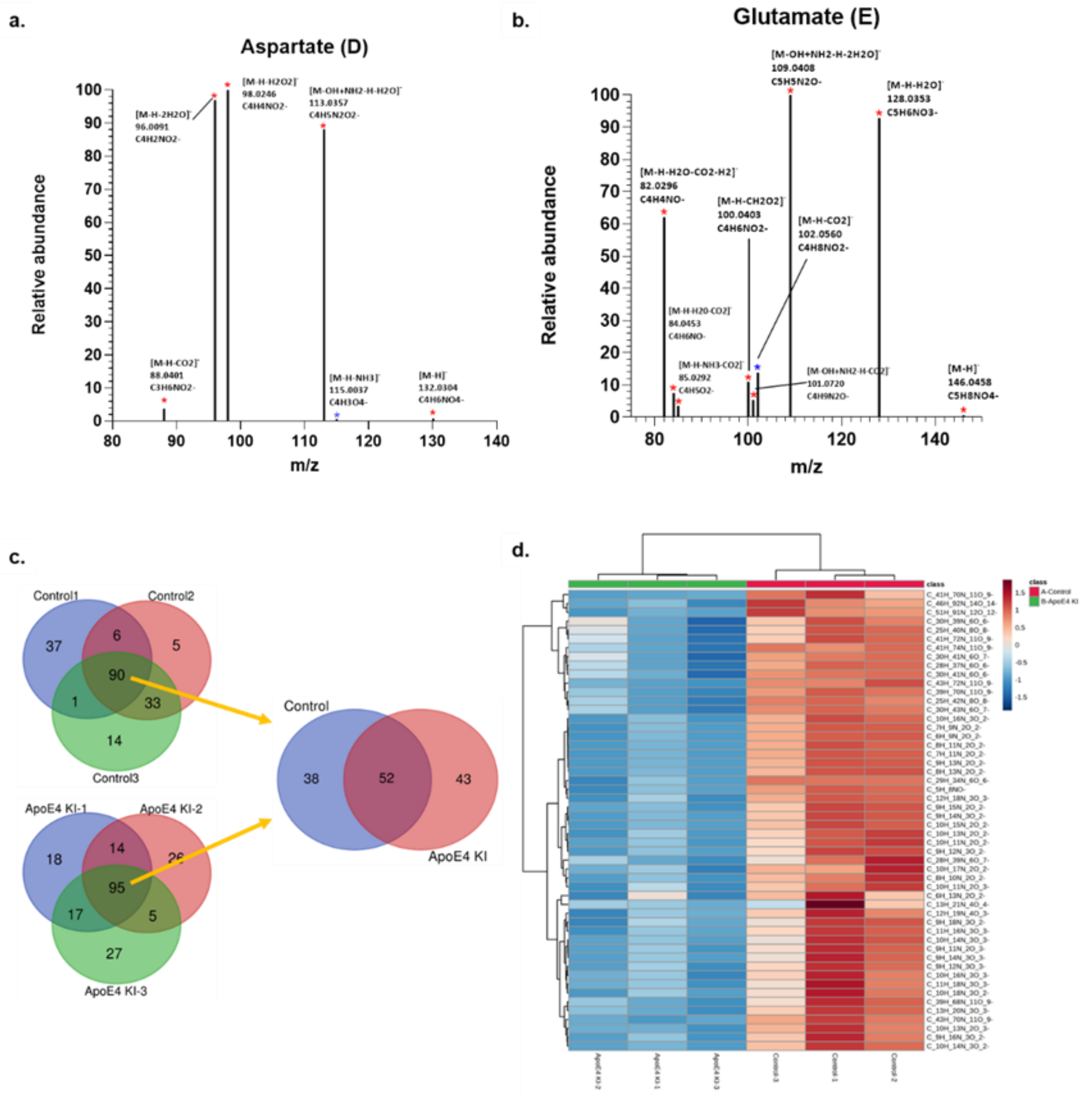

Figure S7. (a) The spectrum of aspartate-related fragments. (b) The spectrum of glutamate-related fragments. The red star means unique fragments for each amino acid assignment; blue star means non-unique fragments which can be produced from amino acids. (c) The Venn diagram of peptide assignment from each of samples and assignments commonly detected from the control and ApoE4 KI groups. (d) The heatmap of 52 common peptide fragments from the control and ApoE4 KI groups.

Table S10. The number of peptide assignment after applying different chemical filtering restriction.

Sample name	Control1	Control2	Control3	ApoE4 KI-1	ApoE4 KI-2	ApoE4 KI-3
Ion peaks	3012	3012	3012	6871	7091	6829
Elemental restriction	6793	6773	6823	6193	6851	7616
DBE and N, O, H to C ratio restriction	134	134	138	144	140	144

Table S11. Peptide assignments from each of three Control H4 samples

Control1			Control2			Control3		
Mass	Normalized intensity	Chemical formula	Mass	Normalized intensity	Chemical formula	Mass	Normalized intensity	Chemical formula

98.0611	0.000204	C5H8NO-	98.0611	0.000204	C5H8NO-	98.0611	0.000178	C5H8NO-
141.067	0.000676	C6H9N2O2-	141.067	0.000676	C6H9N2O2-	141.067	0.00051	C6H9N2O2-
145.0982	4.29E-06	C6H13N2O2-	145.098	4.29E-06	C6H13N2O2-	145.0982	4.43E-06	C6H13N2O2-
153.0669	0.00149	C7H9N2O2-	153.067	0.00149	C7H9N2O2-	153.0669	0.00114	C7H9N2O2-
155.0826	0.000683	C7H11N2O2-	155.083	0.000683	C7H11N2O2-	155.0826	0.000509	C7H11N2O2-
166.0748	8.89E-06	C8H10N2O2-	166.075	8.89E-06	C8H10N2O2-	166.0748	5.26E-06	C8H10N2O2-
167.0826	0.000592	C8H11N2O2-	167.083	0.000592	C8H11N2O2-	167.0826	0.000451	C8H11N2O2-
169.0982	0.000352	C8H13N2O2-	169.098	0.000352	C8H13N2O2-	169.0983	0.000235	C8H13N2O2-
181.0983	0.000385	C9H13N2O2-	181.098	0.000385	C9H13N2O2-	181.0983	0.000271	C9H13N2O2-
183.1139	5.84E-05	C9H15N2O2-	183.114	5.84E-05	C9H15N2O2-	183.1139	3.85E-05	C9H15N2O2-
191.0826	3.38E-05	C10H11N2O2-	191.083	3.38E-05	C10H11N2O2-	191.0827	1.79E-05	C10H11N2O2-
193.0982	2.25E-05	C10H13N2O2-	193.098	2.25E-05	C10H13N2O2-	193.0983	1.30E-05	C10H13N2O2-
194.0935	9.98E-05	C9H12N3O2-	194.094	9.98E-05	C9H12N3O2-	194.0935	5.39E-05	C9H12N3O2-
195.0775	3.00E-05	C9H11N2O3-	195.078	3.00E-05	C9H11N2O3-	195.0775	1.81E-05	C9H11N2O3-
195.1139	0.000148	C10H15N2O2-	195.114	0.000148	C10H15N2O2-	195.1139	9.61E-05	C10H15N2O2-
196.1092	9.37E-05	C9H14N3O2-	196.109	9.37E-05	C9H14N3O2-	196.1092	5.95E-05	C9H14N3O2-
197.1296	8.93E-06	C10H17N2O2-	197.13	8.93E-06	C10H17N2O2-	197.1296	6.08E-06	C10H17N2O2-
198.1249	4.47E-05	C9H16N3O2-	198.125	4.47E-05	C9H16N3O2-	198.1249	3.26E-05	C9H16N3O2-
200.1405	9.69E-06	C9H18N3O2-	200.141	9.69E-06	C9H18N3O2-	200.1406	5.28E-06	C9H18N3O2-
207.0776	8.21E-06	C10H11N2O3-	207.078	8.21E-06	C10H11N2O3-	207.0775	4.79E-06	C10H11N2O3-
208.1092	9.96E-05	C10H14N3O2-	208.109	9.96E-05	C10H14N3O2-	208.1092	6.84E-05	C10H14N3O2-
209.0932	1.10E-05	C10H13N2O3-	209.093	1.10E-05	C10H13N2O3-	209.0932	9.45E-06	C10H13N2O3-
210.0885	0.000123	C9H12N3O3-	210.089	0.000123	C9H12N3O3-	210.0885	9.18E-05	C9H12N3O3-
210.1249	4.15E-05	C10H16N3O2-	210.125	4.15E-05	C10H16N3O2-	210.1249	3.12E-05	C10H16N3O2-
212.1041	3.63E-05	C9H14N3O3-	212.104	3.63E-05	C9H14N3O3-	212.1042	2.14E-05	C9H14N3O3-
212.1405	3.13E-05	C10H18N3O2-	212.141	3.13E-05	C10H18N3O2-	212.1406	1.89E-05	C10H18N3O2-
224.1042	5.48E-05	C10H14N3O3-	224.104	5.48E-05	C10H14N3O3-	224.1042	2.81E-05	C10H14N3O3-
226.1199	2.18E-05	C10H16N3O3-	226.12	2.18E-05	C10H16N3O3-	226.1199	1.68E-05	C10H16N3O3-
238.1199	2.22E-05	C11H16N3O3-	238.12	2.22E-05	C11H16N3O3-	238.1199	1.35E-05	C11H16N3O3-
240.13	2.28E-	C11H18N3	240.1	2.28E-	C11H18N3O	240.13	1.58E-	C11H18N3O

55	05	O3-	36	05	3-	56	05	3-
252.13 56	7.35E-06	C12H18N3 O3-	252.1 36	7.35E-06	C12H18N3O 3-	252.13 56	5.89E-06	C12H18N3O 3-
266.15 12	5.31E-06	C13H20N3 O3-	266.1 51	5.31E-06	C13H20N3O 3-	266.15 13	3.81E-06	C13H20N3O 3-
267.14 65	3.09E-06	C12H19N4 O3-	267.1 47	3.09E-06	C12H19N4O 3-	267.14 65	2.24E-06	C12H19N4O 3-
297.15 71	3.31E-06	C13H21N4 O4-	297.1 57	3.31E-06	C13H21N4O 4-	297.15 69	1.98E-06	C13H21N4O 4-
553.27 86	1.69E-05	C28H37N6 O6-	553.2 79	1.69E-05	C28H37N6O 6-	553.27 86	1.41E-05	C28H37N6O 6-
562.25 56	5.68E-06	C29H34N6 O6-	562.2 56	5.68E-06	C29H34N6O 6-	562.25 55	5.66E-06	C29H34N6O 6-
571.28 91	5.19E-06	C28H39N6 O7-	571.2 89	5.19E-06	C28H39N6O 7-	571.28 88	2.54E-06	C28H39N6O 7-
579.29 43	5.70E-05	C30H39N6 O6-	579.2 94	5.70E-05	C30H39N6O 6-	579.29 42	4.58E-05	C30H39N6O 6-
580.29 76	1.13E-05	C25H40N8 O8-	580.2 98	1.13E-05	C25H40N8O 8-	580.29 77	8.05E-06	C25H40N8O 8-
581.30 98	9.60E-05	C30H41N6 O6-	581.3 1	9.60E-05	C30H41N6O 6-	581.30 99	8.87E-05	C30H41N6O 6-
582.31 33	2.04E-05	C25H42N8 O8-	582.3 13	2.04E-05	C25H42N8O 8-	582.31 34	2.08E-05	C25H42N8O 8-
597.30 51	1.33E-05	C30H41N6 O7-	597.3 05	1.33E-05	C30H41N6O 7-	597.30 48	1.16E-05	C30H41N6O 7-
599.32 05	3.20E-05	C30H43N6 O7-	599.3 21	3.20E-05	C30H43N6O 7-	599.32 06	3.08E-05	C30H43N6O 7-
834.52 21	5.85E-06	C39H68N11 O9-	834.5 22	5.85E-06	C39H68N11 O9-	834.52 23	2.95E-06	C39H68N11 O9-
836.53 79	2.85E-05	C39H70N11 O9-	836.5 38	2.85E-05	C39H70N11 O9-	836.53 78	2.48E-05	C39H70N11 O9-
860.53 83	4.04E-06	C41H70N11 O9-	860.5 38	4.04E-06	C41H70N11 O9-	860.53 78	5.70E-06	C41H70N11 O9-
862.55 35	6.67E-05	C41H72N11 O9-	862.5 54	6.67E-05	C41H72N11 O9-	862.55 33	4.39E-05	C41H72N11 O9-
864.56 9	5.27E-05	C41H74N11 O9-	864.5 69	5.27E-05	C41H74N11 O9-	864.56 9	5.02E-05	C41H74N11 O9-
884.53 78	1.04E-05	C43H70N11 O9-	884.5 38	1.04E-05	C43H70N11 O9-	884.53 8	9.11E-06	C43H70N11 O9-
886.55 34	5.72E-05	C43H72N11 O9-	886.5 53	5.72E-05	C43H72N11 O9-	886.55 33	4.84E-05	C43H72N11 O9-
1063.6 89	7.41E-06	C51H91N12 O12-	1063. 69	7.41E-06	C51H91N12 O12-	1063.6 891	9.49E-06	C51H91N12 O12-
1064.6 93	4.45E-06	C46H92N14 O14-	1064. 69	4.45E-06	C46H92N14 O14-	1064.6 929	6.38E-06	C46H92N14 O14-
86.060 9	7.24E-06	C4H8NO-	86.06 09	7.24E-06	C4H8NO-	86.060 9	4.44E-06	C4H8NO-
100.07 68	6.33E-06	C5H10NO-	100.0 77	6.33E-06	C5H10NO-	100.07 68	5.04E-06	C5H10NO-
168.09 05	4.80E-06	C8H12N2O 2-	168.0 91	4.80E-06	C8H12N2O2-	168.09 04	2.90E-06	C8H12N2O2-
179.08 26	9.86E-05	C9H11N2O 2-	179.0 83	9.86E-05	C9H11N2O2-	179.08 26	6.70E-05	C9H11N2O2-
206.09 35	2.79E-05	C10H12N3 O2-	206.0 94	2.79E-05	C00H32N3O 2S-	206.09 35	1.96E-05	C10H12N3O 2-
222.08 85	3.95E-06	C10H12N3 O3-	222.0 89	3.95E-06	C10H12N3O 3-	222.08 86	4.59E-06	C10H12N3O 3-
236.10 41	4.62E-06	C11H14N3 O3-	236.1 04	4.62E-06	C11H14N3O 3-	236.10 42	3.05E-06	C11H14N3O 3-

389.2099	4.38E-06	C16H31N5O4-	389.21	4.38E-06	C16H31N5O4S-	389.21	3.83E-06	C16H31N5O4S-
391.2257	5.78E-05	C16H33N5O4-	391.226	5.78E-05	C16H33N5O4S-	391.2257	5.62E-05	C16H33N5O4S-
405.2412	2.56E-06	C17H35N5O4-	405.241	2.56E-06	C17H35N5O4S-	405.2412	3.99E-06	C17H35N5O4S-
417.2413	0.000107	C18H35N5O4-	417.241	0.000107	C18H35N5O4S-	417.2413	8.75E-05	C18H35N5O4S-
575.2234	2.14E-06	C27H29N9O6-	575.223	2.14E-06	C27H29N9O6-	575.2252	2.03E-06	C29H31N6O7-
764.5165	9.58E-06	C38H68N8O8-	764.517	9.58E-06	C38H68N8O8-	575.2252	2.03E-06	C27H29N9O6-
772.53	1.10E-05	C36H70N9O9-	772.53	1.10E-05	C36H70N9O9-	764.517	2.02E-06	C38H68N8O8-
790.5202	2.99E-06	C39H68N9O8-	790.52	2.99E-06	C39H68N9O8-	772.5303	3.91E-06	C36H70N9O9-
798.5342	2.86E-06	C37H70N10O9-	798.534	2.86E-06	C37H70N10O9-	798.5343	2.70E-06	C37H70N10O9-
804.4966	1.32E-05	C37H64N12O8-	804.497	1.32E-05	C37H64N12O8-	804.4968	4.04E-06	C37H64N12O8-
804.4966	1.32E-05	C36H68N8O12-	804.497	1.32E-05	C36H68N8O12-	804.4968	4.04E-06	C36H68N8O12-
804.4966	1.32E-05	C34H66N11O11-	804.497	1.32E-05	C34H66N11O11-	804.4968	4.04E-06	C34H66N11O11-
806.4942	3.96E-06	C34H68N11O9-	806.494	3.96E-06	C34H68N11O9S-	810.5819	4.82E-05	C40H76N9O8-
810.5815	1.06E-05	C40H76N9O8-	810.582	1.06E-05	C40H76N9O8-	812.523	3.99E-06	C36H68N12O9-
812.5227	1.19E-05	C36H68N12O9-	812.523	1.19E-05	C36H68N12O9-	813.5102	2.55E-06	C38H69N8O11-
813.5111	3.09E-06	C38H69N8O11-	813.511	3.09E-06	C38H69N8O11-	814.5084	9.23E-06	C40H66N10O8-
814.508	1.56E-05	C40H66N10O8-	814.508	1.56E-05	C40H66N10O8-	814.5084	9.23E-06	C37H70N10O8S-
814.521	3.68E-06	C38H72N9O8-	814.521	3.68E-06	C38H72N9O8S-	814.5214	3.29E-06	C38H72N9O8S-
828.4966	2.39E-05	C39H64N12O8-	828.497	2.39E-05	C39H64N12O8-	828.4969	7.99E-06	C41H66N9O9-
828.4966	2.39E-05	C38H68N8O12-	828.497	2.39E-05	C38H68N8O12-	828.4969	7.99E-06	C39H64N12O8-
828.4966	2.39E-05	C36H66N11O11-	828.497	2.39E-05	C36H66N11O11-	828.4969	7.99E-06	C38H68N8O12-
830.4942	6.65E-06	C38H70N8O10-	830.494	6.65E-06	C38H70N8O10S-	828.4969	7.99E-06	C36H66N11O11-
830.4942	6.65E-06	C36H68N11O9-	830.494	6.65E-06	C36H68N11O9S-	830.494	3.75E-06	C38H70N8O10S-
833.5188	1.55E-05	C38H73N8O10-	833.519	1.55E-05	C38H73N8O10S-	830.494	3.75E-06	C36H68N11O9S-
834.5221	5.85E-06	C36H72N11O9-	834.522	5.85E-06	C36H72N11O9S-	830.5212	2.86E-06	C37H70N10O11-
850.4787	2.77E-05	C40H66N8O12-	850.479	2.77E-05	C40H66N8O12-	833.5186	7.26E-06	C38H73N8O10S-
850.4787	2.77E-05	C38H64N11O11-	850.479	2.77E-05	C38H64N11O11-	834.5223	2.95E-06	C36H72N11O9S-
852.4765	1.04E-05	C40H68N8O10-	852.477	1.04E-05	C40H68N8O10S-	850.4793	7.97E-06	C40H66N8O12-
852.4765	1.04E-05	C38H66N11O9-	852.477	1.04E-05	C38H66N11O9S-	850.4793	7.97E-06	C38H64N11O11-
857.51	5.15E-	C40H73N8	857.5	5.15E-	C40H73N8O	852.47	4.00E-	C40H68N8O

86	06	O10-	19	06	10S-	66	06	10S-
858.52 16	1.88E-06	C41H68N11 O9-	858.5 22	1.88E-06	C41H68N11 O9-	852.47 66	4.00E-06	C38H66N11 O9S-
859.53 44	1.56E-05	C40H75N8 O10-	859.5 34	1.56E-05	C40H75N8O 10S-	857.51 88	4.26E-06	C40H73N8O 10S-
860.53 83	4.04E-06	C38H74N11 O9-	860.5 38	4.04E-06	C38H74N11 O9S-	858.52 25	2.14E-06	C41H68N11 O9-
862.55 35	6.67E-05	C38H76N11 O9-	862.5 54	6.67E-05	C38H76N11 O9S-	858.52 25	2.14E-06	C38H72N11 O9S-
866.45 28	1.72E-05	C41H60N11 O10-	866.4 53	1.72E-05	C41H60N11 O10-	859.53 47	1.07E-05	C40H75N8O 10S-
868.47 72	6.53E-06	C39H66N9 O13-	868.4 77	6.53E-06	C39H66N9O 13-	860.53 78	5.70E-06	C38H74N11 O9S-
868.47 72	6.53E-06	C37H64N12 O12-	868.4 77	6.53E-06	C37H64N12 O12-	862.55 33	4.39E-05	C38H76N11 O9S-
870.47 5	3.78E-06	C42H64N9 O11-	870.4 75	3.78E-06	C42H64N9O 11-	866.45 26	6.51E-06	C41H60N11 O10-
870.47 5	3.78E-06	C39H68N9 O11-	870.4 75	3.78E-06	C39H68N9O 11S-	868.47 68	3.72E-06	C39H66N9O 13-
870.47 5	3.78E-06	C37H66N12 O10-	870.4 75	3.78E-06	C37H66N12 O10S-	868.47 68	3.72E-06	C37H64N12 O12-
872.50 05	4.61E-06	C41H66N11 O10-	872.5 01	4.61E-06	C41H66N11 O10-	870.47 31	3.96E-06	C42H64N9O 11-
873.54 9	5.52E-06	C39H75N11 O9-	873.5 49	5.52E-06	C39H75N11 O9S-	870.47 31	3.96E-06	C40H62N12 O10-
875.56 62	8.44E-06	C38H75N12 O11-	875.5 66	8.44E-06	C38H75N12 O11-	870.47 31	3.96E-06	C37H66N12 O10S-
883.53 39	2.72E-05	C40H73N11 O9-	883.5 34	2.72E-05	C40H73N11 O9S-	872.50 3	1.46E-06	C40H72N8O 11S-
884.53 78	1.04E-05	C40H74N11 O9-	884.5 38	1.04E-05	C40H74N11 O9S-	872.50 3	1.46E-06	C38H70N11 O10S-
886.55 34	5.72E-05	C40H76N11 O9-	886.5 53	5.72E-05	C40H76N11 O9S-	873.54 81	4.32E-06	C39H75N11 O9S-
888.56 93	6.03E-05	C43H74N11 O9-	888.5 69	6.03E-05	C43H74N11 O9-	875.56 81	1.15E-05	C40H77N9O 12-
888.56 93	6.03E-05	C40H78N11 O9-	888.5 69	6.03E-05	C40H78N11 O9S-	875.56 81	1.15E-05	C38H75N12 O11-
890.45 79	5.24E-06	C40H64N11 O10-	890.4 58	5.24E-06	C40H64N11 O10S-	884.53 8	9.11E-06	C40H74N11 O9S-
890.58 5	2.33E-05	C43H76N11 O9-	890.5 85	2.33E-05	C43H76N11 O9-	886.55 33	4.84E-05	C40H76N11 O9S-
906.43 23	3.57E-06	C43H60N11 O9-	906.4 32	3.57E-06	C43H60N11 O9S-	888.57 01	7.92E-05	C40H78N11 O9S-
911.56 63	1.35E-05	C41H75N12 O11-	911.5 66	1.35E-05	C41H75N12 O11-	890.45 53	1.10E-06	C40H64N11 O10S-
912.56 89	6.77E-06	C45H74N11 O9-	912.5 69	6.77E-06	C45H74N11 O9-	890.58 42	4.96E-05	C43H76N11 O9-
912.56 89	6.77E-06	C42H78N11 O9-	912.5 69	6.77E-06	C42H78N11 O9S-	901.58 21	5.61E-06	C40H77N12 O11-
913.58 09	1.38E-05	C42H79N11 O9-	913.5 81	1.38E-05	C42H79N11 O9S-	906.43 15	1.77E-06	C43H60N11 O9S-
914.58 43	6.01E-06	C45H76N11 O9-	914.5 84	6.01E-06	C45H76N11 O9-	911.56 67	1.85E-05	C41H75N12 O11-
916.45 88	1.65E-06	C43H66N9 O11-	916.4 59	1.65E-06	C43H66N9O 11S-	912.57 15	1.77E-05	C42H78N11 O9S-
916.45 88	1.65E-06	C41H64N12 O10-	916.4 59	1.65E-06	C41H64N12 O10S-	913.58 1	1.63E-05	C42H79N11 O9S-
930.43 37	8.97E-06	C44H58N12 O11-	930.4 34	8.97E-06	C44H58N12 O11-	914.45 94	2.30E-06	C41H62N12 O12-

932.4315	5.94E-06	C44H60N12O9-	932.432	5.94E-06	C44H60N12O9S-	914.5857	8.52E-06	C42H80N11O9S-
932.4315	5.94E-06	C41H62N11O12-	932.432	5.94E-06	C41H62N11O12S-	916.4589	2.06E-06	C43H66N9O11S-
946.4135	3.39E-06	C44H58N12O10-	946.414	3.39E-06	C44H58N12O10S-	916.4589	2.06E-06	C41H64N12O10S-
968.4004	6.15E-06	C45H54N13O12-	968.4	6.15E-06	C45H54N13O12-	930.4336	4.72E-06	C44H58N12O11-
970.3986	3.12E-06	C50H54N10O11-	970.399	3.12E-06	C50H54N10O11-	932.4325	3.40E-06	C44H60N12O9S-
970.3986	3.12E-06	C48H52N13O10-	970.399	3.12E-06	C48H52N13O10-	932.4325	3.40E-06	C41H62N11O12S-
970.3986	3.12E-06	C45H56N13O10-	970.399	3.12E-06	C45H56N13O10S-	946.4167	1.35E-06	C45H58N10O13-
970.3986	3.12E-06	C44H60N9O14-	970.399	3.12E-06	C44H60N9O14S-	946.4167	1.35E-06	C43H56N13O12-
970.3986	3.12E-06	C42H58N12O13-	970.399	3.12E-06	C42H58N12O13S-	968.4005	3.23E-06	C45H54N13O12-
984.3753	3.16E-06	C50H52N10O12-	984.375	3.16E-06	C50H52N10O12-	970.3985	1.11E-06	C50H54N10O11-
984.3753	3.16E-06	C48H50N13O11-	984.375	3.16E-06	C48H50N13O11-	970.3985	1.11E-06	C48H52N13O10-
984.3753	3.16E-06	C45H52N12O14-	984.375	3.16E-06	C45H52N12O14-	970.3985	1.11E-06	C45H56N13O10S-
984.3753	3.16E-06	C42H56N12O14-	984.375	3.16E-06	C42H56N12O14S-	970.3985	1.11E-06	C44H60N9O14S-
1063.689	7.41E-06	C49H89N15O11-	1063.69	7.41E-06	C49H89N15O11-	970.3985	1.11E-06	C42H58N12O13S-
1063.689	7.41E-06	C46H93N15O11-	1063.69	7.41E-06	C46H93N15O11S-	984.3744	1.99E-06	C48H50N13O11-
1064.693	4.45E-06	C49H90N15O11-	1064.69	4.45E-06	C49H90N15O11-	984.3744	1.99E-06	C45H52N12O14-
						984.3744	1.99E-06	C42H56N12O14S-
						1063.6891	9.49E-06	C49H89N15O11-
						1063.6891	9.49E-06	C46H93N15O11S-
						1064.6929	6.38E-06	C49H90N15O11-

Table S12. Peptide assignments from each of three ApoE4 KI H4 samples

**ApoE4 KI1**			**ApoE4 KI2**			**ApoE4 KI3**		
Mass	Normalized intensity	Chemical formula	Mass	Normalized intensity	Chemical formula	Mass	Normalized intensity	Chemical formula
98.0611	8.52E-05	C5H8NO-	98.061	6.07E-05	C5H8NO-	98.061	7.15E-05	C5H8NO-
141.067	1.91E-04	C6H9N2O2-	141.067	1.37E-04	C6H9N2O2-	141.067	1.47E-04	C6H9N2O2-
145.098	3.91E-06	C6H13N2O2-	145.098	2.43E-06	C6H13N2O2-	145.098	2.39E-06	C6H13N2O2-
153.067	4.78E-04	C7H9N2O2-	153.067	3.39E-04	C7H9N2O2-	153.067	3.69E-04	C7H9N2O2-
155.083	1.62E-04	C7H11N2O2-	155.083	1.21E-04	C7H11N2O2-	155.082	1.26E-04	C7H11N2O2-
166.075	2.12E-06	C8H10N2O2-	166.075	1.48E-06	C8H10N2O2-	166.075	1.32E-06	C8H10N2O2-

167.083	1.58E-04	C8H11N2O2-	167.082	1.22E-04	C8H11N2O2-	167.082	1.27E-04	C8H11N2O2-
169.098	7.72E-05	C8H13N2O2-	169.098	5.51E-05	C8H13N2O2-	169.098	5.75E-05	C8H13N2O2-
181.098	8.44E-05	C9H13N2O2-	181.098	6.35E-05	C9H13N2O2-	181.098	6.63E-05	C9H13N2O2-
183.114	1.82E-05	C9H15N2O2-	183.114	1.49E-05	C9H15N2O2-	183.114	1.28E-05	C9H15N2O2-
191.083	9.02E-06	C10H11N2O2-	191.083	6.69E-06	C10H11N2O2-	191.083	6.02E-06	C10H11N2O2-
193.098	6.94E-06	C10H13N2O2-	193.098	4.09E-06	C10H13N2O2-	193.098	4.38E-06	C10H13N2O2-
194.093	2.49E-05	C9H12N3O2-	194.093	1.64E-05	C9H12N3O2-	194.093	1.72E-05	C9H12N3O2-
195.077	1.11E-05	C9H11N2O3-	195.077	8.09E-06	C9H11N2O3-	195.077	7.25E-06	C9H11N2O3-
195.114	3.29E-05	C10H15N2O2-	195.114	2.31E-05	C10H15N2O2-	195.114	2.46E-05	C10H15N2O2-
196.109	2.29E-05	C9H14N3O2-	196.109	1.67E-05	C9H14N3O2-	196.109	1.73E-05	C9H14N3O2-
197.13	3.41E-06	C10H17N2O2-	197.13	2.87E-06	C10H17N2O2-	197.13	2.10E-06	C10H17N2O2-
198.125	1.42E-05	C9H16N3O2-	198.125	1.01E-05	C9H16N3O2-	198.125	9.10E-06	C9H16N3O2-
200.14	3.66E-06	C9H18N3O2-	200.14	1.79E-06	C9H18N3O2-	200.141	2.47E-06	C9H18N3O2-
207.078	2.64E-06	C10H11N2O3-	207.077	1.20E-06	C10H11N2O3-	207.078	1.09E-06	C10H11N2O3-
208.109	2.21E-05	C10H14N3O2-	208.109	1.81E-05	C10H14N3O2-	208.109	1.66E-05	C10H14N3O2-
209.093	4.37E-06	C10H13N2O3-	209.093	4.34E-06	C10H13N2O3-	209.093	3.66E-06	C10H13N2O3-
210.088	6.37E-05	C9H12N3O3-	210.088	5.04E-05	C9H12N3O3-	210.088	4.59E-05	C9H12N3O3-
210.125	1.10E-05	C10H16N3O2-	210.125	7.44E-06	C10H16N3O2-	210.125	7.11E-06	C10H16N3O2-
212.104	1.34E-05	C9H14N3O3-	212.104	9.88E-06	C9H14N3O3-	212.104	9.78E-06	C9H14N3O3-
212.14	1.08E-05	C10H18N3O2-	212.14	6.35E-06	C10H18N3O2-	212.14	6.83E-06	C10H18N3O2-
224.104	1.60E-05	C10H14N3O3-	224.104	1.18E-05	C10H14N3O3-	224.104	1.14E-05	C10H14N3O3-
226.12	1.03E-05	C10H16N3O3-	226.12	8.98E-06	C10H16N3O3-	226.12	6.97E-06	C10H16N3O3-
238.12	6.86E-06	C11H16N3O3-	238.12	4.11E-06	C11H16N3O3-	238.12	5.08E-06	C11H16N3O3-
240.135	1.05E-05	C11H18N3O3-	240.135	8.98E-06	C11H18N3O3-	240.135	7.52E-06	C11H18N3O3-
252.136	3.13E-06	C12H18N3O3-	252.135	1.98E-06	C12H18N3O3-	252.135	1.95E-06	C12H18N3O3-
266.151	1.81E-06	C13H20N3O3-	266.151	2.11E-06	C13H20N3O3-	266.151	1.50E-06	C13H20N3O3-
267.146	9.75E-07	C12H19N4O3-	267.146	4.35E-07	C12H19N4O3-	267.146	7.25E-07	C12H19N4O3-
297.157	1.55E-06	C13H21N4O4-	297.157	1.59E-06	C13H21N4O4-	297.157	1.14E-06	C13H21N4O4-
553.279	6.13E-06	C28H37N6O6-	553.279	8.60E-06	C28H37N6O6-	553.279	4.34E-06	C28H37N6O6-
562.2	1.76E-	C29H34N6O	562.2	9.41E-	C29H34N6O	562.2	1.25E-	C29H34N6O

55	06	6-	55	07	6-	55	06	6-
571.289	1.23E-06	C28H39N6O7-	571.289	1.71E-06	C28H39N6O7-	571.289	9.82E-07	C28H39N6O7-
579.294	2.27E-05	C30H39N6O6-	579.294	3.85E-05	C30H39N6O6-	579.294	1.44E-05	C30H39N6O6-
580.298	3.89E-06	C25H40N8O8-	580.298	6.45E-06	C25H40N8O8-	580.298	2.53E-06	C25H40N8O8-
581.31	4.61E-05	C30H41N6O6-	581.31	5.67E-05	C30H41N6O6-	581.31	3.35E-05	C30H41N6O6-
582.313	8.25E-06	C25H42N8O8-	582.313	1.09E-05	C25H42N8O8-	582.313	6.59E-06	C25H42N8O8-
597.305	4.68E-06	C30H41N6O7-	597.305	7.61E-06	C30H41N6O7-	597.305	3.09E-06	C30H41N6O7-
599.321	1.30E-05	C30H43N6O7-	599.32	1.72E-05	C30H43N6O7-	599.321	1.05E-05	C30H43N6O7-
834.522	8.78E-07	C39H68N11O9-	834.522	1.31E-06	C39H68N11O9-	834.522	5.53E-07	C39H68N11O9-
836.538	6.23E-06	C39H70N11O9-	836.538	9.25E-06	C39H70N11O9-	836.538	4.63E-06	C39H70N11O9-
860.538	1.05E-06	C41H70N11O9-	860.538	9.51E-07	C41H70N11O9-	860.537	1.14E-06	C41H70N11O9-
862.554	1.52E-05	C41H72N11O9-	862.554	2.97E-05	C41H72N11O9-	862.554	8.61E-06	C41H72N11O9-
864.57	1.47E-05	C41H74N11O9-	864.569	2.20E-05	C41H74N11O9-	864.57	9.45E-06	C41H74N11O9-
884.538	3.34E-06	C43H70N11O9-	884.538	3.80E-06	C43H70N11O9-	884.538	3.66E-06	C43H70N11O9-
886.554	1.74E-05	C43H72N11O9-	886.553	1.94E-05	C43H72N11O9-	886.554	1.57E-05	C43H72N11O9-
1063.69	3.03E-06	C51H91N12O12-	1063.69	2.49E-06	C51H91N12O12-	1063.69	2.72E-06	C51H91N12O12-
1064.69	1.57E-06	C46H92N14O14-	1064.69	1.25E-06	C46H92N14O14-	1064.69	9.02E-07	C46H92N14O14-
86.0609	3.33E-06	C4H8NO-	86.0609	1.82E-06	C4H8N1O1-	86.0609	1.70E-06	C4H8NO-
100.077	3.05E-06	C5H10NO-	100.077	2.06E-06	C5H10N1O1-	100.077	2.20E-06	C5H10NO-
143.083	1.73E-06	C6H11N2O2-	140.059	4.02E-07	C6H8N2O2-	143.083	1.06E-06	C6H11N2O2-
154.075	6.91E-07	C7H10N2O2-	143.083	1.30E-06	C6H11N2O2-	154.075	4.82E-07	C7H10N2O2-
157.098	1.95E-06	C7H13N2O2-	154.075	4.02E-07	C7H10N2O2-	157.098	1.21E-06	C7H13N2O2-
168.09	1.06E-06	C8H12N2O2-	157.098	1.24E-06	C7H13N2O2-	168.09	8.22E-07	C8H12N2O2-
171.114	1.63E-06	C8H15N2O2-	171.114	8.12E-07	C8H15N2O2-	171.114	9.05E-07	C8H15N2O2-
173.039	1.02E-06	C6H9N2O2S-	173.039	5.46E-07	C6H9N2O2S-	173.039	7.16E-07	C6H9N2O2S-
179.083	2.44E-05	C9H11N2O2-	179.082	1.86E-05	C9H11N2O2S-	179.083	1.84E-05	C9H11N2O2-
180.09	5.37E-07	C9H12N2O2-	185.039	4.50E-07	C7H9N2O2S-	180.09	4.53E-07	C9H12N2O2-
185.039	1.02E-06	C7H9N2O2S-	197.093	6.04E-07	C9H13N2O3-	185.039	1.02E-06	C7H9N2O2S-
197.093	9.39E-07	C9H13N2O3-	206.093	4.55E-06	C10H12N3O2-	197.093	7.50E-07	C9H13N2O3-
206.093	6.80E-06	C10H12N3O2-	211.109	6.20E-07	C10H15N2O3-	206.093	4.68E-06	C10H12N3O2-

211.1<br>09	5.58E-<br>07	C10H15N2O<br>3-	222.0<br>88	1.22E-<br>06	C10H12N3O<br>3S-	211.0<br>96	5.93E-<br>07	C9H13N3O3-
214.1<br>2	5.51E-<br>07	C9H16N3O3-	230.0<br>73	6.69E-<br>07	C9H14N2O3<br>S-	214.1<br>56	4.67E-<br>07	C10H20N3O<br>2-
214.1<br>56	5.27E-<br>07	C10H20N3O<br>2-	236.1<br>04	9.24E-<br>07	C11H14N3O<br>3S-	222.0<br>88	1.66E-<br>06	C10H12N3O<br>3-
222.0<br>88	1.76E-<br>06	C10H12N3O<br>3-	240.0<br>82	8.31E-<br>07	C10H14N3O<br>2S-	236.1<br>04	8.20E-<br>07	C11H14N3O3<br>-
236.1<br>04	1.38E-<br>06	C11H14N3O<br>3-	242.0<br>61	4.99E-<br>07	C9H12N3O3<br>S-	240.0<br>82	9.45E-<br>07	C10H14N3O<br>2S-
240.0<br>57	6.79E-<br>07	C10H12N2O<br>3S-	244.0<br>77	8.31E-<br>07	C9H14N3O3<br>S-	254.1<br>51	1.05E-<br>06	C12H20N3O<br>3-
240.0<br>82	1.12E-<br>06	C10H14N3O<br>2S-	250.1<br>2	6.37E-<br>07	C12H16N3O<br>3-	269.1<br>62	7.91E-<br>07	C12H21N4O<br>3-
244.0<br>77	6.09E-<br>07	C9H14N3O3<br>S-	254.1<br>51	1.15E-<br>06	C12H20N3O<br>3-	281.1<br>26	5.30E-<br>07	C12H17N4O<br>4-
250.1<br>2	7.38E-<br>07	C12H16N3O<br>3-	260.1<br>04	4.41E-<br>07	C13H14N3O<br>3-	281.1<br>62	6.37E-<br>07	C13H21N4O<br>3-
254.1<br>51	9.64E-<br>07	C12H20N3O<br>3-	265.1<br>31	4.01E-<br>07	C12H17N4O<br>3-	283.1<br>41	7.90E-<br>07	C12H19N4O<br>4-
258.0<br>92	6.22E-<br>07	C10H16N3O<br>3S-	269.1<br>62	6.16E-<br>07	C12H21N4O<br>3-	283.1<br>78	6.59E-<br>07	C13H23N4O<br>3-
269.1<br>62	8.58E-<br>07	C12H21N4O<br>3-	280.1<br>67	4.43E-<br>07	C14H22N3O<br>3-	295.1<br>41	5.41E-<br>07	C13H19N4O<br>4-
274.1<br>2	9.27E-<br>07	C14H16N3O<br>3-	281.1<br>26	4.21E-<br>07	C12H17N4O<br>4-	328.2<br>36	6.57E-<br>07	C15H30N5O<br>3-
279.1<br>46	5.53E-<br>07	C13H19N4O<br>3-	281.1<br>62	8.73E-<br>07	C13H21N4O<br>3-	389.2<br>1	1.17E-<br>06	C16H31N5O<br>4S-
280.1<br>67	6.12E-<br>07	C14H22N3O<br>3-	283.1<br>41	1.07E-<br>06	C12H19N4O<br>4-	391.2<br>26	1.49E-<br>05	C16H33N5O<br>4S-
281.1<br>26	8.59E-<br>07	C12H17N4O<br>4-	283.1<br>78	7.33E-<br>07	C13H23N4O<br>3-	405.2<br>41	1.26E-<br>06	C17H35N5O<br>4S-
281.1<br>62	8.02E-<br>07	C13H21N4O<br>3-	295.1<br>41	6.08E-<br>07	C13H19N4O<br>4-	415.2<br>26	9.31E-<br>07	C18H33N5O<br>4S-
283.1<br>41	1.30E-<br>06	C12H19N4O<br>4-	311.1<br>73	7.26E-<br>07	C14H23N4O<br>4-	417.2<br>41	3.11E-<br>05	C18H35N5O<br>4S-
283.1<br>78	1.32E-<br>06	C13H23N4O<br>3-	323.1<br>73	4.21E-<br>07	C15H23N4O<br>4-	436.2<br>56	4.59E-<br>07	C21H34N5O<br>5-
295.1<br>41	7.53E-<br>07	C13H19N4O<br>4-	328.2<br>36	8.62E-<br>07	C15H30N5O<br>3-	439.1<br>87	6.40E-<br>07	C22H25N5O<br>5-
309.1<br>57	5.79E-<br>07	C14H21N4O<br>4-	389.2<br>1	2.42E-<br>06	C16H31N5O<br>4S-	439.2<br>26	6.29E-<br>07	C20H33N5O<br>4S-
311.1<br>73	6.42E-<br>07	C14H23N4O<br>4-	391.2<br>26	2.42E-<br>05	C16H33N5O<br>4S-	445.2<br>73	5.21E-<br>07	C20H39N5O<br>4S-
323.1<br>73	5.56E-<br>07	C15H23N4O<br>4-	405.2<br>41	1.21E-<br>06	C17H35N5O<br>4S-	465.1<br>67	6.61E-<br>07	C23H23N5O<br>6-
389.2<br>1	1.57E-<br>06	C16H31N5O<br>4S-	410.2<br>4	4.01E-<br>07	C19H32N5O<br>5-	583.3<br>16	7.35E-<br>07	C26H45N7O<br>6S-
391.2<br>26	1.96E-<br>05	C16H33N5O<br>4S-	415.2<br>26	1.60E-<br>06	C18H33N5O<br>4S-	655.2<br>21	5.74E-<br>07	C30H35N6O<br>9S-
405.2<br>41	1.39E-<br>06	C17H35N5O<br>4S-	417.2<br>41	6.56E-<br>05	C18H35N5O<br>4S-	673.2<br>32	1.95E-<br>06	C33H35N7O<br>7S-
415.2<br>26	8.67E-<br>07	C18H33N5O<br>4S-	431.2<br>57	4.03E-<br>07	C19H37N5O<br>4S-	812.5<br>14	7.39E-<br>07	C37H68N10<br>O10-
417.2<br>41	4.68E-<br>05	C18H35N5O<br>4S-	437.1<br>71	5.49E-<br>07	C22H23N5O<br>5-	833.5<br>19	1.41E-<br>06	C38H73N8O<br>10S-
436.2<br>56	7.95E-<br>07	C21H34N5O<br>5-	439.2<br>25	6.09E-<br>07	C20H33N5O<br>4S-	834.5<br>22	5.53E-<br>07	C36H72N11O<br>9S-
438.2	2.99E-	C21H36N5O	441.2	4.56E-	C20H35N5O	835.2	8.77E-	C37H43N10

71	06	5-	41	07	4S-	84	07	O11S-
441.2 41	8.12E-07	C20H35N5O 4S-	445.2 72	1.07E-06	C20H39N5O 4S-	847.4 3	7.01E-07	C38H59N10 O12-
445.2 73	5.66E-07	C20H39N5O 4S-	575.2 3	4.40E-07	C26H35N6O 7S-	857.5 19	1.12E-06	C40H73N8O 10S-
575.2 14	5.54E-07	C28H29N7O 7-	583.3 16	1.09E-06	C26H45N7O 6S-	858.5 21	6.17E-07	C41H68N11O 9-
583.3 16	1.17E-06	C26H45N7O 6S-	583.3 25	4.67E-07	C30H43N6O 6-	859.5 35	2.14E-06	C40H75N8O 10S-
601.2 78	1.19E-06	C24H41N8O 8S-	601.2 78	1.22E-06	C24H41N8O 8S-	859.5 35	2.14E-06	C37H71N12 O11-
603.2 94	8.00E-07	C24H43N8O 8S-	603.2 94	7.74E-07	C24H43N8O 8S-	860.3 39	4.21E-07	C44H46N9O 10-
671.2 16	6.68E-07	C33H33N7O 7S-	605.3 1	4.34E-07	C24H45N8O 8S-	860.3 39	4.21E-07	C41H50N9O 10S-
671.2 16	6.68E-07	C30H35N6O 10S-	607.3 26	4.43E-07	C24H47N8O 8S-	860.3 39	4.21E-07	C39H48N12 O9S-
673.2 32	1.93E-06	C33H35N7O 7S-	673.2 31	1.79E-06	C33H35N7O 7S-	860.3 57	1.51E-06	C41H50N9O 12-
729.2 57	4.93E-07	C34H37N10 O7S-	673.2 31	1.79E-06	C30H37N6O 10S-	860.3 57	1.51E-06	C39H48N12 O11-
826.5 02	8.79E-07	C36H66N12 O10-	812.5 11	4.63E-07	C37H68N10 O10-	860.3 8	1.29E-06	C39H50N13 O10-
833.5 19	2.28E-06	C38H73N8O 10S-	812.5 25	4.86E-07	C38H70N9O 10-	860.3 97	7.00E-07	C42H54N9O 11-
834.5 22	8.78E-07	C36H72N11 O9S-	812.5 25	4.86E-07	C36H68N12 O9-	860.3 97	7.00E-07	C39H58N9O 11S-
835.2 84	8.81E-07	C37H43N10 O11S-	833.5 19	3.79E-06	C38H73N8O 10S-	860.3 97	7.00E-07	C37H56N12 O10S-
840.4 94	7.21E-07	C37H66N11 O11-	834.5 22	1.31E-06	C36H72N11 O9S-	860.4 31	8.01E-07	C43H58N9O 10-
840.5 27	9.01E-07	C39H72N10 O8S-	835.2 84	8.20E-07	C37H43N10 O11S-	860.4 31	8.01E-07	C41H56N12 O9-
847.4 31	7.98E-07	C38H59N10 O12-	845.4 15	4.29E-07	C38H57N10 O12-	861.4 3	4.88E-07	C39H61N10 O10S-
849.5 51	1.10E-06	C36H73N12 O11-	847.4 3	9.34E-07	C38H59N10 O12-	862.5 54	8.61E-06	C38H76N11O 9S-
857.5 2	1.91E-06	C40H73N8O 10S-	850.5 52	4.86E-07	C40H72N11 O9-	873.5 49	5.72E-07	C39H75N11O 9S-
857.5 2	1.91E-06	C37H69N12 O11-	857.5 19	1.48E-06	C40H73N8O 10S-	876.5 69	4.48E-07	C42H74N11O 9-
858.5 24	8.02E-07	C40H74N8O 10S-	858.5 23	5.09E-07	C41H68N11 O9-	884.5 38	3.66E-06	C40H74N11O 9S-
858.5 24	8.02E-07	C38H72N11 O9S-	858.5 23	5.09E-07	C38H72N11 O9S-	886.5 54	1.57E-05	C40H76N11O 9S-
859.5 35	2.30E-06	C40H75N8O 10S-	859.5 34	3.93E-06	C40H75N8O 10S-	887.5 66	1.88E-05	C39H75N12 O11-
859.5 35	2.30E-06	C37H71N12 O11-	860.5 38	9.51E-07	C38H74N11 O9S-	888.5 69	8.29E-06	C43H74N11O 9-
860.5 38	1.05E-06	C38H74N11 O9S-	862.5 54	2.97E-05	C38H76N11 O9S-	888.5 69	8.29E-06	C40H78N11O 9S-
862.5 54	1.52E-05	C38H76N11 O9S-	871.4 3	4.68E-07	C40H59N10 O12-	889.5 71	3.99E-07	C40H77N10 O12-
873.5 51	6.13E-07	C38H73N12 O11-	871.5 7	7.06E-07	C40H77N11 O8S-	889.5 82	3.57E-06	C39H77N12 O11-
876.5 7	6.60E-07	C42H74N11 O9-	875.5 65	2.38E-06	C39H77N11 O9S-	892.6	4.96E-07	C43H78N11O 9-
884.5 38	3.34E-06	C40H74N11 O9S-	876.5 7	1.14E-06	C42H74N11 O9-	901.5 82	8.88E-07	C40H77N12 O11-

886.554	1.74E-05	C40H76N11O9S-	884.538	3.80E-06	C40H74N11O9S-	902.585	6.09E-07	C44H76N11O9-
887.566	2.36E-05	C39H75N12O11-	886.553	1.94E-05	C40H76N11O9S-	902.585	6.09E-07	C41H80N11O9S-
888.57	1.01E-05	C43H74N11O9-	888.569	1.22E-05	C43H74N11O9-	909.551	3.23E-06	C41H73N12O11-
888.57	1.01E-05	C40H78N11O9S-	888.569	1.22E-05	C40H78N11O9S-	910.554	1.40E-06	C45H72N11O9-
889.582	6.45E-06	C39H77N12O11-	890.585	6.10E-06	C43H76N11O9-	910.554	1.40E-06	C42H76N11O9S-
890.585	3.36E-06	C43H76N11O9-	892.6	8.78E-07	C43H78N11O9-	911.566	4.74E-06	C41H75N12O11-
892.6	4.88E-07	C43H78N11O9-	902.584	6.42E-07	C44H76N11O9-	912.57	2.44E-06	C45H74N11O9-
901.582	7.63E-07	C40H77N12O11-	907.535	4.25E-07	C41H71N12O11-	912.57	2.44E-06	C42H78N11O9S-
902.585	5.49E-07	C44H76N11O9-	910.553	2.36E-06	C45H72N11O9-	914.584	1.24E-06	C45H76N11O9-
902.585	5.49E-07	C41H80N11O9S-	912.569	2.99E-06	C45H74N11O9-	915.596	5.93E-07	C42H81N11O9S-
910.554	1.99E-06	C42H76N11O9S-	912.569	2.99E-06	C42H78N11O9S-	1063.69	2.72E-06	C46H93N15O11S-
911.566	5.91E-06	C41H75N12O11-	914.585	1.84E-06	C45H76N11O9-	1064.69	9.02E-07	C49H90N15O11-
912.57	2.52E-06	C45H74N11O9-	914.585	1.84E-06	C42H80N11O9S-	1123.72	1.32E-06	C53H97N13O11S-
912.57	2.52E-06	C42H78N11O9S-	915.596	8.05E-07	C42H81N11O9S-	1124.34	6.12E-07	C54H50N11O17-
914.585	1.61E-06	C45H76N11O9-	1063.69	2.49E-06	C49H89N15O11-	1124.34	6.12E-07	C49H52N14O16S-
914.585	1.61E-06	C42H80N11O9S-	1063.69	2.49E-06	C46H93N15O11S-	1124.4	4.70E-07	C58H54N13O12-
915.595	7.83E-07	C42H81N11O9S-				1124.4	4.70E-07	C55H56N12O15-
1063.69	3.03E-06	C49H89N15O11-				1124.4	4.70E-07	C53H54N15O14-
1063.69	3.03E-06	C46H93N15O11S-				1124.4	4.70E-07	C52H60N12O15S-
1064.69	1.57E-06	C49H90N15O11-				1124.4	4.70E-07	C50H58N15O14S-

S6. Proteomics data analysis and GO analysis

Proteomics analysis

-Sample Preparation

Cells were washed twice by PBS. Cell pellets were collected in microcentrifuge tubes by removing and discarding the supernatant through centrifugation at 13,000 rpm for 5 minutes at 4°C. Then cell pellets were incubated in lysis buffer (8M Urea, 4% CHAPs in 30mM Tris buffer, pH = 8.5) for 30 minutes on ice, with vortexing at 10min intervals. Transfer the extract to microcentrifuge tubes and centrifuge at 13,000 rpm for 10 minutes at 4°C. Aliquot the clear lysate to clean microcentrifuge tubes. These samples are ready for assay. Lysates can be stored at -80°C. Avoid multiple freezes/thaws.

A filter-aided sample preparation (FASP) method was used to generate enzymatic peptides, which means the protein denature, reduction, alkylation and trypsin digestion were performed on the membrane of a cartridge with a molecular weight cut-off (MWCO) of 10KDa (Vivacon 500, Sartorius).

25 µg of lysates were denatured and disulphide bonds were reduced with 200µL of 20 mM Tris(2-carboxyethyl)phosphine hydrochloride (TCEP)/8 M urea in 200 mM Triethylammonium bicarbonate (TEAB) buffer, pH 8.5. Alkylation was performed by adding 200µL of 50 mM Iodoacetamide (IAA) in TEAB buffer. Trypsin digestion was carried out at 370C for 16 hours with an enzyme:protein ratio of 1:25. Between steps, the samples were cleaned with TEAB buffer. Protein digests were eluted with 0.1% TFA in H2O, 0.1% TFA in 50% acetonitrile in H2O and 0.1% TFA in acetonitrile by centrifugation, the 3 fractions obtained were pooled and solvent was removed using Eppendrof Concentrator Plus (Eppendrof).

-Peptides Separation and Mass Spectrometric Data Acquisition

Tryptic peptides separation and mass spectrometric analysis was performed on an Vanquish™ Neo UHPLC System coupled with an Orbitrap Eclipse mass spectrometer (Thermo Fisher Scientific). The data were recorded using Xcalibur 4.4 software (Thermo Fisher Scientific).

After removing solvent, tryptic peptides were resuspended in 100 µL of 0.1% trifluoroacetic acid in H2O. For the chromatographic separation of peptides, 4 µL (1 µg) of resuspended solution was loaded on an Acclaim™ PepMap™ 100 C18 HPLC Column (500 mm, 75 µm i.d., 3 µm and 100 Å, Thermo Fisher Scientific). The column oven was set as 40°C. Peptides were separated with a 120-minute linear gradient from 5% to 28% buffer B (80% acetonitrile in H2O, 0.1% formic acid), i.e., 95% to 72% buffer A (2% acetonitrile in H2O, 0.1% formic acid) over 105 minutes, then to 40% B over 15 minutes, finally to 95% B over 10 minutes and kept at 95% for 10 minutes, at a flow rate of 300nL/min. The column was connected to an Thermo Easy-Spray capillary Emitter.

MS1 spectra were acquired in the Orbitrap (Resolution = 120,000, Scan range = 175 - 1800, RF Lens = 40%, AGC target = Standard and Maximum injection time mode = Auto). Charge states between 2 and 7 were required for MS2 analysis, and a 20 s dynamic exclusion window was used. Cycle time was set at 2 second.

MS2 fragmentation was performed in the ion trap with normalized HCD collision energy of 30 (Isolation mode = Quadrupole, isolation window = 1.6 Da, Auto scan range mode). MS2 spectra were acquired in the orbitrap (Resolution = 15,000, Maximum injection time = 35 ms, AGC target = 50,000, Normalised AGC target = 100% and Auto scan range).

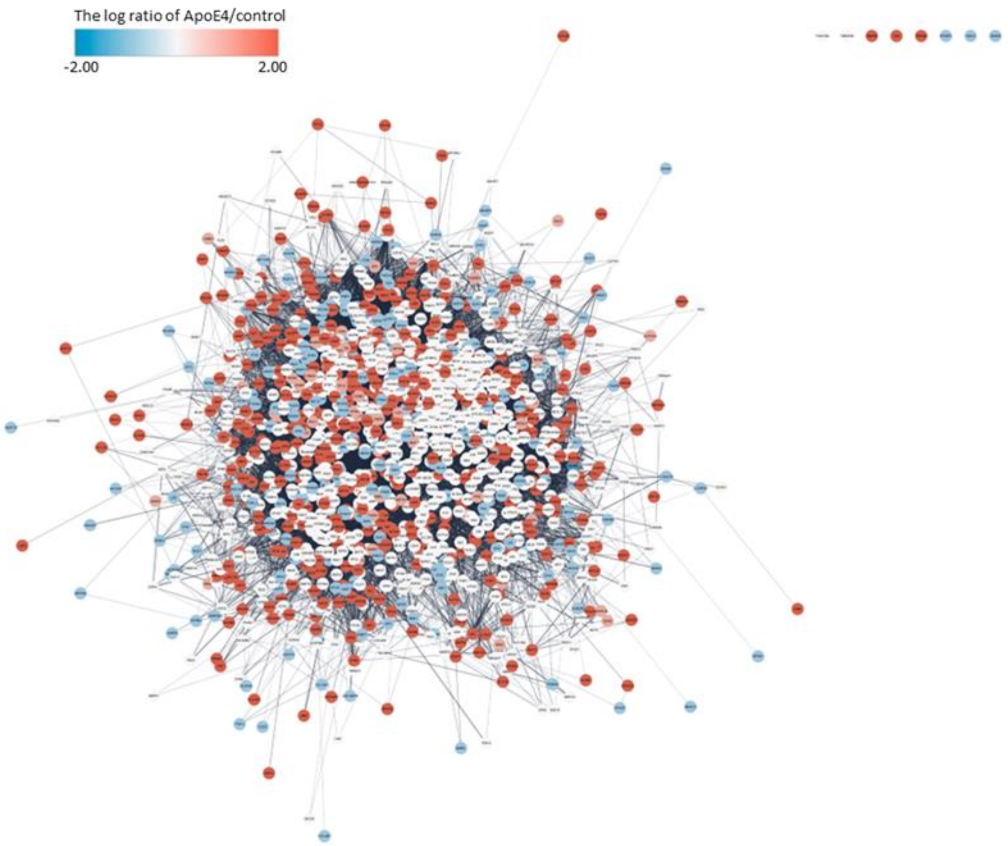

Figure S8. The protein network built by StringApp, the nodes (1458) present each protein, and edges (26953) present the interaction between proteins. Red nodes represent up-regulated proteins in the ApoE4-KI group compared with Control H4 cells. Blue nodes represent down-regulated proteins.

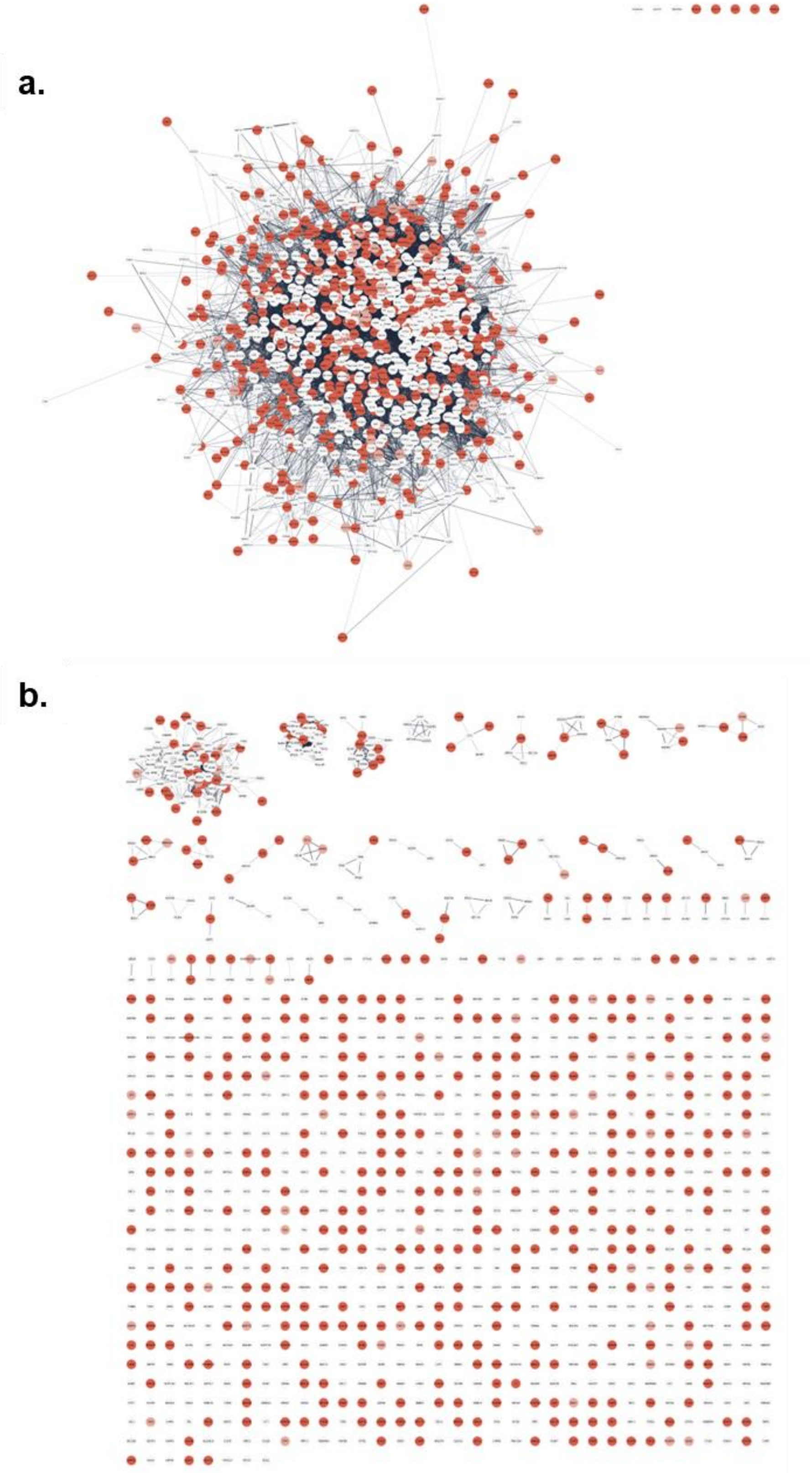

Figure S9. a) STRING network of proteins with up-regulated level in ApoE4-KI cells. b) Clustered protein association network of up-regulated proteins. Log-ratios between ApoE4-carried group and control group for each protein were mapped to the nodes using a blue-white-red gradient.

Table S13. The list of clustered up-regulated proteins (101)

Description	Display name	stringdb canonical name	Sample/control	Average abundance	Log ratio
Queuine tRNA-ribosyltransferase accessory subunit 2 OS=Homo sapiens OX=9606 GN=QTRT2 PE=1 SV=1	QTRTD1	Q9H974	100	5.15E+07	2
Translation initiation factor eIF-2B subunit delta OS=Homo sapiens OX=9606 GN=EIF2B4 PE=1 SV=2	EIF2B4	Q9UI10	100	1.20E+07	2
High affinity cationic amino acid transporter 1 OS=Homo sapiens OX=9606 GN=SLC7A1 PE=1 SV=1	SLC7A1	P30825	100	1.03E+07	2
Exosome complex exonuclease RRP44 OS=Homo sapiens OX=9606 GN=DIS3 PE=1 SV=2	DIS3	Q9Y2L1	100	1.73E+07	2
Lipid droplet-regulating VLDL assembly factor AUP1 OS=Homo sapiens OX=9606 GN=AUP1 PE=1 SV=2	AUP1	Q9Y679	100	6725753.583	2
D-aminoacyl-tRNA deacylase 1 OS=Homo sapiens OX=9606 GN=DTD1 PE=1 SV=2	DTD1	Q8TEA8	100	3.37E+07	2
Exosome component 10 OS=Homo sapiens OX=9606 GN=EXOSC10 PE=1 SV=2	EXOSC10	Q01780	100	8.78E+07	2
Pseudouridylate synthase 1 homolog OS=Homo sapiens OX=9606 GN=PUS1 PE=1 SV=3	PUS1	Q9Y606	100	5477207.417	2
28S ribosomal protein S16, mitochondrial OS=Homo sapiens OX=9606 GN=MRPS16 PE=1 SV=1	MRPS16	Q9Y3D3	100	6497335	2
28S ribosomal protein S2, mitochondrial OS=Homo sapiens OX=9606 GN=MRPS2 PE=1 SV=1	MRPS2	Q9Y399	100	7955604.833	2
39S ribosomal protein L9, mitochondrial OS=Homo sapiens OX=9606 GN=MRPL9 PE=1 SV=2	MRPL9	Q9BYD2	100	5610027.333	2
Deaminated glutathione amidase OS=Homo sapiens OX=9606 GN=NIT1 PE=1 SV=2	NIT1	Q86X76	100	8856956.5	2
Ribosome-recycling factor, mitochondrial OS=Homo sapiens OX=9606 GN=MRRF	MRRF	Q96E11	100	6166503	2

PE=1 SV=1					
Dihydropyrimidinase-related protein 4 OS=Homo sapiens OX=9606 GN=DPYSL4 PE=1 SV=2	DPYSL4	O14531	100	5944561.5	2
Peptide deformylase, mitochondrial OS=Homo sapiens OX=9606 GN=PDF PE=1 SV=1	PDF	Q9HBH1	100	2971142.667	2
Deoxyribonuclease TATDN1 OS=Homo sapiens OX=9606 GN=TATDN1 PE=1 SV=2	TATDN1	Q6P1N9	100	3127302.75	2
Phosphoribosyl pyrophosphate synthase-associated protein 2 OS=Homo sapiens OX=9606 GN=PRPSAP2 PE=1 SV=1	PRPSAP2	O60256	100	2.89E+07	2
2-hydroxyacyl-CoA lyase 2 OS=Homo sapiens OX=9606 GN=ILVBL PE=1 SV=2	ILVBL	A1L0T0	100	3.18E+07	2
Probable cysteine--tRNA ligase, mitochondrial OS=Homo sapiens OX=9606 GN=CARS2 PE=1 SV=1	CARS2	Q9HA77	100	3329514.625	2
Elongator complex protein 3 OS=Homo sapiens OX=9606 GN=ELP3 PE=1 SV=2	ELP3	Q9H9T3	100	4304961.833	2
GTPase Era, mitochondrial OS=Homo sapiens OX=9606 GN=ERAL1 PE=1 SV=2	ERAL1	O75616	100	2810566.333	2
Guanine deaminase OS=Homo sapiens OX=9606 GN=GDA PE=1 SV=1	GDA	Q9Y2T3	100	5096648.458	2
Mitofusin-2 OS=Homo sapiens OX=9606 GN=MFN2 PE=1 SV=3	MFN2	O95140	100	9723892	2
Vesicle transport protein GOT1B OS=Homo sapiens OX=9606 GN=GOLT1B PE=1 SV=1	GOLT1B	Q9Y3E0	100	1.01E+07	2
39S ribosomal protein L32, mitochondrial OS=Homo sapiens OX=9606 GN=MRPL32 PE=1 SV=1	MRPL32	Q9BYC8	100	3182432.813	2
ATP-dependent RNA helicase DHX30 OS=Homo sapiens OX=9606 GN=DHX30 PE=1 SV=1	DHX30	Q7L2E3	50	7.75E+07	1
ATP-binding cassette sub-family F member 1 OS=Homo sapiens OX=9606 GN=ABCF1 PE=1 SV=2	ABCF1	Q8NE71	29	2.54E+08	1
ATP-dependent 6-phosphofructokinase, liver type OS=Homo sapiens OX=9606	PFKL	P17858	26	1.14E+08	1

GN=PFKL PE=1 SV=6					
Protein RRP5 homolog OS=Homo sapiens OX=9606 GN=PDCD11 PE=1 SV=3	PDCD11	Q14690	12	2.86E+07	1
Heat shock 70 kDa protein 4L OS=Homo sapiens OX=9606 GN=HSPA4L PE=1 SV=3	HSPA4L	O95757	11	2.48E+07	1
Tetratricopeptide repeat protein 4 OS=Homo sapiens OX=9606 GN=TTC4 PE=1 SV=3	TTC4	O95801	9	6.81E+07	0
CAD protein OS=Homo sapiens OX=9606 GN=CAD PE=1 SV=3	CAD	P27708	8	2.49E+07	0
Nucleolar GTP-binding protein 2 OS=Homo sapiens OX=9606 GN=GNL2 PE=1 SV=1	GNL2	Q13823	7	1.76E+07	0
RNA cytidine acetyltransferase OS=Homo sapiens OX=9606 GN=NAT10 PE=1 SV=2	NAT10	Q9H0A0	7	2.46E+08	0
Isoleucine--tRNA ligase, cytoplasmic OS=Homo sapiens OX=9606 GN=IARS1 PE=1 SV=2	IARS	P41252	6	2.50E+08	0
Translation initiation factor eIF-2B subunit epsilon OS=Homo sapiens OX=9606 GN=EIF2B5 PE=1 SV=3	EIF2B5	Q13144	6	5.73E+07	0
Bifunctional glutamate/proline--tRNA ligase OS=Homo sapiens OX=9606 GN=EPRS1 PE=1 SV=5	EPRS	P07814	5	5.48E+08	0
Zinc phosphodiesterase ELAC protein 2 OS=Homo sapiens OX=9606 GN=ELAC2 PE=1 SV=2	ELAC2	Q9BQ52	5	3.42E+07	0
DnaJ homolog subfamily C member 10 OS=Homo sapiens OX=9606 GN=DNAJC10 PE=1 SV=2	DNAJC10	Q8IXB1	5	1.57E+07	0
ATP-binding cassette sub-family F member 3 OS=Homo sapiens OX=9606 GN=ABCF3 PE=1 SV=2	ABCF3	Q9NUQ8	4	2.79E+07	0
Leucine--tRNA ligase, cytoplasmic OS=Homo sapiens OX=9606 GN=LARS1 PE=1 SV=2	LARS	Q9P2J5	4	3.04E+08	0
Protein arginine N-methyltransferase 3 OS=Homo sapiens OX=9606 GN=PRMT3 PE=1 SV=4	PRMT3	O60678	4	3.92E+07	0
Branched-chain-amino-acid aminotransferase, mitochondrial OS=Homo	BCAT2	O15382	4	2.21E+07	0

sapiens OX=9606 GN=BCAT2 PE=1 SV=2					
Tyrosine--tRNA ligase, mitochondrial OS=Homo sapiens OX=9606 GN=YARS2 PE=1 SV=2	YARS2	Q9Y2Z4	4	3.76E+07	0
C-1-tetrahydrofolate synthase, cytoplasmic OS=Homo sapiens OX=9606 GN=MTHFD1 PE=1 SV=4	MTHFD1	P11586	4	1.07E+09	0
40S ribosomal protein S13 OS=Homo sapiens OX=9606 GN=RPS13 PE=1 SV=2	RPS13	P62277	3	5.61E+08	0
Fumarylacetoacetase OS=Homo sapiens OX=9606 GN=FAH PE=1 SV=2	FAH	P16930	3	3.24E+07	0
Pyrroline-5-carboxylate reductase 1, mitochondrial OS=Homo sapiens OX=9606 GN=PYCR1 PE=1 SV=2	PYCR1	P32322	3	9.67E+07	0
Adenylate kinase 4, mitochondrial OS=Homo sapiens OX=9606 GN=AK4 PE=1 SV=1	AK4	P27144	3	2.47E+07	0
Adenosine 3'-phospho 5'-phosphosulfate transporter 1 OS=Homo sapiens OX=9606 GN=SLC35B2 PE=1 SV=1	SLC35B2	Q8TB61	3	4.16E+07	0
Valine--tRNA ligase OS=Homo sapiens OX=9606 GN=VARS1 PE=1 SV=4	VARS	P26640	3	6.36E+08	0
DnaJ homolog subfamily C member 9 OS=Homo sapiens OX=9606 GN=DNAJC9 PE=1 SV=1	DNAJC9	Q8WXX5	3	3.61E+07	0
Trifunctional enzyme subunit beta, mitochondrial OS=Homo sapiens OX=9606 GN=HADHB PE=1 SV=3	HADHB	P55084	3	3.91E+07	0
Cytoplasmic aconitate hydratase OS=Homo sapiens OX=9606 GN=ACO1 PE=1 SV=3	ACO1	P21399	3	2.59E+07	0
Cleavage and polyadenylation specificity factor subunit 2 OS=Homo sapiens OX=9606 GN=CPSF2 PE=1 SV=2	CPSF2	Q9P2I0	3	1.10E+07	0
Cytosolic Fe-S cluster assembly factor NUBP2 OS=Homo sapiens OX=9606 GN=NUBP2 PE=1 SV=1	NUBP2	Q9Y5Y2	3	2.42E+07	0
39S ribosomal protein L44, mitochondrial OS=Homo sapiens OX=9606	MRPL44	Q9H9J2	3	1.16E+07	0

GN=MRPL44 PE=1 SV=1					
Alanine--tRNA ligase, mitochondrial OS=Homo sapiens OX=9606 GN=AARS2 PE=1 SV=1	AARS2	Q5JTZ9	3	6.59E+07	0
Fumarylacetoacetate hydrolase domain-containing protein 2A OS=Homo sapiens OX=9606 GN=FAHD2A PE=1 SV=1	FAHD2A	Q96GK7	3	8231655.375	0
Pyruvate dehydrogenase protein X component, mitochondrial OS=Homo sapiens OX=9606 GN=PDHX PE=1 SV=3	PDHX	O00330	3	1.41E+07	0
Nuclear factor NF-kappa-B p105 subunit OS=Homo sapiens OX=9606 GN=NFKB1 PE=1 SV=2	NFKB1	P19838	3	4013145.958	0
DnaJ homolog subfamily C member 3 OS=Homo sapiens OX=9606 GN=DNAJC3 PE=1 SV=1	DNAJC3	Q13217	2	1.39E+07	0
Phosphoribosyl pyrophosphate synthase-associated protein 1 OS=Homo sapiens OX=9606 GN=PRPSAP1 PE=1 SV=2	PRPSAP1	Q14558	2	9120914.333	0
Vitamin K epoxide reductase complex subunit 1-like protein 1 OS=Homo sapiens OX=9606 GN=VKORC1L1 PE=1 SV=2	VKORC1L1	Q8N0U8	2	2.01E+07	0
Alpha-aminoadipic semialdehyde dehydrogenase OS=Homo sapiens OX=9606 GN=ALDH7A1 PE=1 SV=5	ALDH7A1	P49419	2	3.23E+07	0
Omega-amidase NIT2 OS=Homo sapiens OX=9606 GN=NIT2 PE=1 SV=1	NIT2	Q9NQR4	2	2.02E+07	0
Succinate--CoA ligase [ADP/GDP-forming] subunit alpha, mitochondrial OS=Homo sapiens OX=9606 GN=SUCLG1 PE=1 SV=4	SUCLG1	P53597	2	2.24E+07	0
Acylpyruvase FAHD1, mitochondrial OS=Homo sapiens OX=9606 GN=FAHD1 PE=1 SV=2	FAHD1	Q6P587	2	9451819.167	0
Trifunctional purine biosynthetic protein adenosine-3 OS=Homo sapiens OX=9606 GN=GART PE=1 SV=1	GART	P22102	2	5.73E+08	0
Succinate--CoA ligase [ADP-forming] subunit beta, mitochondrial OS=Homo sapiens OX=9606 GN=SUCLA2 PE=1 SV=3	SUCLA2	Q9P2R7	2	1.03E+07	0

Leucine-rich repeat-containing protein 47 OS=Homo sapiens OX=9606 GN=LRRC47 PE=1 SV=1	LRRC47	Q8N1G4	2	1.07E+08	0
Heat shock 70 kDa protein 1B OS=Homo sapiens OX=9606 GN=HSPA1B PE=1 SV=1	HSPA1B	P0DMV9	2	1.19E+09	0
CTP synthase 1 OS=Homo sapiens OX=9606 GN=CTPS1 PE=1 SV=2	CTPS1	P17812	2	2.11E+08	0
Ornithine aminotransferase, mitochondrial OS=Homo sapiens OX=9606 GN=OAT PE=1 SV=1	OAT	P04181	2	2.76E+08	0
NADH-cytochrome b5 reductase 1 OS=Homo sapiens OX=9606 GN=CYB5R1 PE=1 SV=1	CYB5R1	Q9UHQ9	2	4464292.5	0
Isoleucine--tRNA ligase, mitochondrial OS=Homo sapiens OX=9606 GN=IARS2 PE=1 SV=2	IARS2	Q9NSE4	2	1.38E+08	0
NADH-cytochrome b5 reductase 3 OS=Homo sapiens OX=9606 GN=CYB5R3 PE=1 SV=3	CYB5R3	P00387	2	2.26E+08	0
Lon protease homolog, mitochondrial OS=Homo sapiens OX=9606 GN=LONP1 PE=1 SV=2	LONP1	P36776	2	3.06E+08	0
Serine hydroxymethyltransferase, mitochondrial OS=Homo sapiens OX=9606 GN=SHMT2 PE=1 SV=3	SHMT2	P34897	2	5.33E+08	0
Eukaryotic initiation factor 4A-II OS=Homo sapiens OX=9606 GN=EIF4A2 PE=1 SV=2	EIF4A2	Q14240	2	4.42E+07	0
Lysine--tRNA ligase OS=Homo sapiens OX=9606 GN=KARS1 PE=1 SV=3	KARS	Q15046	2	3.35E+08	0
Elongation factor Ts, mitochondrial OS=Homo sapiens OX=9606 GN=TSFM PE=1 SV=2	TSFM	P43897	2	1.17E+07	0
Glutamine--tRNA ligase OS=Homo sapiens OX=9606 GN=QARS1 PE=1 SV=1	QARS	P47897	2	1.87E+08	0
Argininosuccinate lyase OS=Homo sapiens OX=9606 GN=ASL PE=1 SV=4	ASL	P04424	2	7343384.583	0
39S ribosomal protein L39, mitochondrial OS=Homo sapiens OX=9606	MRPL39	Q9NYK5	2	2.04E+07	0

GN=MRPL39 PE=1 SV=3					
Peptidyl-tRNA hydrolase ICT1, mitochondrial OS=Homo sapiens OX=9606 GN=MRPL58 PE=1 SV=1	ICT1	Q14197	2	1.09E+07	0
Ribonucleoside-diphosphate reductase large subunit OS=Homo sapiens OX=9606 GN=RRM1 PE=1 SV=1	RRM1	P23921	2	8.06E+07	0
Pyridoxal kinase OS=Homo sapiens OX=9606 GN=PDXK PE=1 SV=1	PDXK	O00764	2	3.44E+07	0
Eukaryotic translation initiation factor 5B OS=Homo sapiens OX=9606 GN=EIF5B PE=1 SV=4	EIF5B	O60841	2	1.03E+08	0
Succinate dehydrogenase [ubiquinone] flavoprotein subunit, mitochondrial OS=Homo sapiens OX=9606 GN=SDHA PE=1 SV=2	SDHA	P31040	2	1.51E+08	0
Oxygen-dependent coproporphyrinogen-III oxidase, mitochondrial OS=Homo sapiens OX=9606 GN=CPOX PE=1 SV=3	CPOX	P36551	2	1.78E+08	0
Methionine--tRNA ligase, cytoplasmic OS=Homo sapiens OX=9606 GN=MARS1 PE=1 SV=2	MARS	P56192	2	2.32E+08	0
T-complex protein 1 subunit eta OS=Homo sapiens OX=9606 GN=CCT7 PE=1 SV=2	CCT7	Q99832	2	6.29E+08	0
ATP-citrate synthase OS=Homo sapiens OX=9606 GN=ACLY PE=1 SV=3	ACLY	P53396	2	7.19E+08	0
CCA tRNA nucleotidyltransferase 1, mitochondrial OS=Homo sapiens OX=9606 GN=TRNT1 PE=1 SV=2	TRNT1	Q96Q11	2	5224249.75	0
Serine--tRNA ligase, cytoplasmic OS=Homo sapiens OX=9606 GN=SARS1 PE=1 SV=3	SARS	P49591	2	2.87E+08	0
Arginine--tRNA ligase, cytoplasmic OS=Homo sapiens OX=9606 GN=RARS1 PE=1 SV=2	RARS	P54136	2	4.25E+08	0
ATP-binding cassette sub-family F member 2 OS=Homo sapiens OX=9606 GN=ABCF2 PE=1 SV=2	ABCF2	Q9UG63	2	1.39E+08	0
Isocitrate dehydrogenase	IDH3G	P51553	2	1.45E+07	0

[NAD] subunit gamma, mitochondrial OS=Homo sapiens OX=9606 GN=IDH3G PE=1 SV=1					
Phosphoenolpyruvate carboxykinase [GTP], mitochondrial OS=Homo sapiens OX=9606 GN=PCK2 PE=1 SV=4	PCK2	Q16822	2	1.68E+08	0
tRNA N6-adenosine threonylcarbamoyltransferase OS=Homo sapiens OX=9606 GN=OSGEP PE=1 SV=1	OSGEP	Q9NPF4	2	1.02E+07	0

Table S14. The enrichment analysis of up-regulated proteins

category	description	FDR value
GO Biological Process	Organonitrogen compound biosynthetic process	1.47E-25
GO Biological Process	Amide biosynthetic process	2.37E-25
GO Biological Process	Cellular nitrogen compound metabolic process	1.11E-23
GO Biological Process	Cellular nitrogen compound biosynthetic process	1.11E-23
GO Biological Process	Translation	1.28E-23
GO Biological Process	Cellular amide metabolic process	1.71E-23
GO Biological Process	Cellular amino acid metabolic process	3.30E-20
GO Biological Process	tRNA metabolic process	4.61E-20
GO Biological Process	Carboxylic acid metabolic process	4.61E-20
GO Biological Process	Organic substance biosynthetic process	4.61E-20
GO Biological Process	Oxoacid metabolic process	8.57E-20
GO Biological Process	Cellular biosynthetic process	1.96E-19
GO Biological Process	Cellular metabolic process	3.99E-19
GO Biological Process	Small molecule metabolic process	8.60E-18
GO Biological Process	Metabolic process	9.69E-18
GO Biological Process	Primary metabolic process	3.05E-17
GO Biological Process	tRNA aminoacylation for protein translation	1.58E-15
GO Biological Process	ncRNA metabolic process	1.58E-15
GO Biological Process	Organic substance metabolic process	1.58E-15
GO Biological Process	Organic cyclic compound metabolic process	1.76E-14
GO Biological Process	Heterocycle metabolic process	6.16E-14

GO Biological Process	Mitochondrial gene expression	6.28E-14
GO Biological Process	Nitrogen compound metabolic process	1.01E-13
GO Biological Process	Gene expression	3.72E-13
GO Biological Process	Cellular aromatic compound metabolic process	5.43E-13
GO Biological Process	Nucleobase-containing compound metabolic process	1.89E-11
GO Biological Process	Mitochondrial translation	3.57E-11
GO Biological Process	Cellular macromolecule biosynthetic process	1.54E-10
GO Biological Process	Organonitrogen compound metabolic process	6.16E-10
GO Biological Process	Alpha-amino acid metabolic process	1.40E-08
GO Biological Process	RNA metabolic process	4.24E-07
GO Biological Process	Mitochondrial translational elongation	6.56E-07
GO Biological Process	Mitochondrial translational termination	7.00E-07
GO Biological Process	tRNA processing	7.11E-07
GO Biological Process	Nucleotide biosynthetic process	1.56E-06
GO Biological Process	Nucleotide metabolic process	1.78E-06
GO Biological Process	Organic cyclic compound biosynthetic process	2.90E-06
GO Biological Process	ncRNA processing	3.73E-06
GO Biological Process	Heterocycle biosynthetic process	5.77E-06
GO Biological Process	Alpha-amino acid biosynthetic process	1.01E-05
GO Biological Process	Tricarboxylic acid cycle	1.25E-05
GO Biological Process	Nucleobase metabolic process	1.86E-05
GO Biological Process	Nucleic acid metabolic process	2.32E-05
GO Biological Process	Glutamine family amino acid metabolic process	2.72E-05
GO Biological Process	Ribose phosphate biosynthetic process	6.83E-05
GO Biological Process	Aromatic compound biosynthetic process	1.20E-04
GO Biological Process	Dicarboxylic acid metabolic process	1.70E-04
GO Biological Process	Regulation of translation	2.60E-04
GO Biological Process	Purine nucleotide metabolic process	2.80E-04
GO Biological Process	Ribose phosphate metabolic process	2.80E-04
GO Biological Process	Oxidation-reduction process	3.10E-04

GO Biological Process	Small molecule biosynthetic process	3.30E-04
GO Biological Process	Posttranscriptional regulation of gene expression	3.40E-04
GO Biological Process	Purine nucleotide biosynthetic process	4.10E-04
GO Biological Process	Organophosphate metabolic process	4.60E-04
GO Biological Process	Cellular protein metabolic process	5.80E-04
GO Biological Process	Carboxylic acid biosynthetic process	5.80E-04
GO Biological Process	Macromolecule metabolic process	6.10E-04
GO Biological Process	Mitochondrial RNA metabolic process	6.20E-04
GO Biological Process	Organophosphate biosynthetic process	8.00E-04
GO Biological Process	Nucleobase biosynthetic process	0.001
GO Biological Process	tRNA modification	0.0014
GO Biological Process	Cellular macromolecule metabolic process	0.0015
GO Biological Process	Cellular process	0.0021
GO Biological Process	Nucleobase-containing compound biosynthetic process	0.0022
GO Biological Process	Ribonucleotide biosynthetic process	0.0036
GO Biological Process	RNA processing	0.0052
GO Biological Process	Ribonucleotide metabolic process	0.0068
GO Biological Process	Serine family amino acid metabolic process	0.0081
GO Biological Process	Mitochondrial tRNA processing	0.0098
GO Biological Process	Carboxylic acid catabolic process	0.01
GO Biological Process	Tricarboxylic acid metabolic process	0.0115
GO Biological Process	Translational initiation	0.0147
GO Biological Process	Pyrimidine nucleobase metabolic process	0.0157
GO Biological Process	Arginine metabolic process	0.0157
GO Biological Process	Glutamine family amino acid biosynthetic process	0.0157
GO Biological Process	glutaminyl-tRNA aminoacylation	0.0196
GO Biological Process	isoleucyl-tRNA aminoacylation	0.0196
GO Biological Process	Ribosome biogenesis	0.0196
GO Biological Process	Tetrahydrofolate metabolic process	0.02
GO Biological Process	Glutamine metabolic process	0.0292

GO Biological Process	Alpha-amino acid catabolic process	0.0292
GO Biological Process	Purine nucleobase metabolic process	0.0322
GO Biological Process	Small molecule catabolic process	0.0443
GO Biological Process	CUT catabolic process	0.0443
KEGG Pathways	Aminoacyl-tRNA biosynthesis	5.31E-16
KEGG Pathways	Metabolic pathways	1.70E-10
KEGG Pathways	Citrate cycle (TCA cycle)	6.31E-08
KEGG Pathways	Biosynthesis of amino acids	1.50E-05
KEGG Pathways	Carbon metabolism	2.40E-04
KEGG Pathways	2-Oxocarboxylic acid metabolism	0.0092
KEGG Pathways	One carbon pool by folate	0.0104
KEGG Pathways	RNA transport	0.0104
KEGG Pathways	Antifolate resistance	0.0247
KEGG Pathways	Ribosome	0.0247
KEGG Pathways	Alanine, aspartate and glutamate metabolism	0.0321

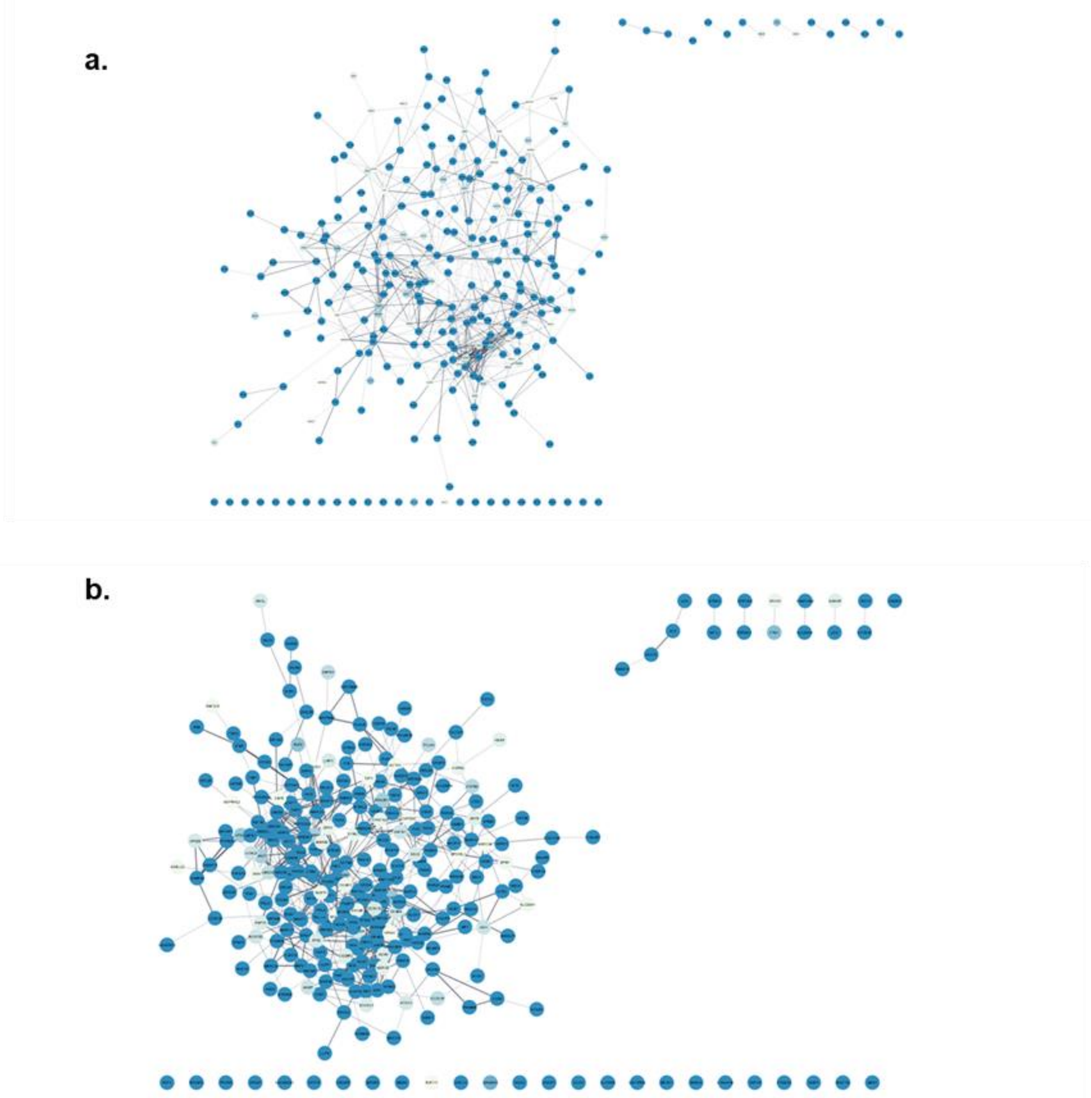

Figure S10. a) STRING network of proteins with down-regulated level in ApoE4-KI cells. b) Clustered protein association network of down-regulated proteins. Log-ratios between ApoE4-carried group and

control group for each protein were mapped to the nodes using a blue-white-red gradient.

Table S15. The list of clustered down-regulated proteins (255)

**stringdb database identifier**	**Description**	**Display name**	**stringdb canonical name**	**Sample/control**	**Average abundance**	**Log ratio**
9606.ENSP00000312618	Complex I assembly factor ACAD9, mitochondrial OS=Homo sapiens OX=9606 GN=ACAD9 PE=1 SV=1	ACAD9	Q9H845	0.001883	3.68E+07	-2.72522
9606.ENSP00000181839	Cyclin-dependent kinase 13 OS=Homo sapiens OX=9606 GN=CDK13 PE=1 SV=2	CDK13	Q14004	0.01	5060617	-2
9606.ENSP00000185150	Endoplasmic reticulum lectin 1 OS=Homo sapiens OX=9606 GN=ERLEC1 PE=1 SV=1	ERLEC1	Q96DZ1	0.01	4385135	-2
9606.ENSP00000203001	tRNA (adenine(58)-N(1))-methyltransferase non-catalytic subunit TRM6 OS=Homo sapiens OX=9606 GN=TRMT6 PE=1 SV=1	TRMT6	Q9UJA5	0.01	3004500	-2
9606.ENSP00000203630	Myeloid leukemia factor 2 OS=Homo sapiens OX=9606 GN=MLF2 PE=1 SV=1	MLF2	Q15773	0.01	1964298	-2
9606.ENSP00000210060	Deoxyhypusine synthase OS=Homo sapiens OX=9606 GN=DHPS PE=1 SV=1	DHPS	P49366	0.01	767330.7	-2
9606.ENSP00000216160	TGF-beta-activated kinase 1 and MAP3K7-binding protein 1 OS=Homo sapiens OX=9606 GN=TAB1 PE=1 SV=1	TAB1	Q15750	0.01	6599838	-2
9606.ENSP00000218224	Polyglutamine-binding protein 1 OS=Homo sapiens OX=9606 GN=PQBP1 PE=1 SV=1	PQBP1	O60828	0.01	1.29E+07	-2
9606.ENSP00000220509	Vacuolar protein sorting-associated protein 18 homolog OS=Homo sapiens OX=9606 GN=VPS18 PE=1 SV=2	VPS18	Q9P253	0.01	6258704	-2
9606.ENSP00000221265	RNA polymerase II-associated factor 1 homolog OS=Homo sapiens OX=9606 GN=PAF1 PE=1 SV=2	PAF1	Q8N7H5	0.01	4557326	-2
9606.ENSP00000221494	Splicing factor 3A subunit 2 OS=Homo sapiens OX=9606 GN=SF3A2 PE=1 SV=2	SF3A2	Q15428	0.01	8940989	-2
9606.ENSP00000221859	DNA-directed RNA polymerase II subunit RPB9 OS=Homo sapiens OX=9606 GN=POLR2I PE=1 SV=1	POLR2I	P36954	0.01	7272635	-2
9606.ENSP00000222803	Peptidyl-prolyl cis-trans isomerase FKBP14 OS=Homo sapiens OX=9606 GN=FKBP14 PE=1 SV=1	FKBP14	Q9NWM8	0.01	2735988	-2
9606.ENSP00000223136	Mitochondrial fission 1 protein OS=Homo sapiens OX=9606 GN=FIS1 PE=1 SV=2	FIS1	Q9Y3D6	0.01	1.02E+07	-2
9606.ENSP00000	Peptidyl-prolyl cis-trans	PPIF	P30405	0.01	545789	-2

225174	isomerase F, mitochondrial OS=Homo sapiens OX=9606 GN=PPIF PE=1 SV=1				7	
9606.ENSP00000 227266	Dipeptidyl peptidase 1 OS=Homo sapiens OX=9606 GN=CTSC PE=1 SV=2	CTSC	P53634	0.01	665414 2	-2
9606.ENSP00000 229195	CCR4-NOT transcription complex subunit 2 OS=Homo sapiens OX=9606 GN=CNOT2 PE=1 SV=1	CNOT2	Q9NZ N8	0.01	189707 7	-2
9606.ENSP00000 230771	Histidine--tRNA ligase, mitochondrial OS=Homo sapiens OX=9606 GN=HARS2 PE=1 SV=1	HARS2	P49590	0.01	379254 0	-2
9606.ENSP00000 232165	Ubiquitin-protein ligase E3A OS=Homo sapiens OX=9606 GN=UBE3A PE=1 SV=4	UBE3A	Q0508 6	0.01	338944 7	-2
9606.ENSP00000 234310	Calcineurin subunit B type 1 OS=Homo sapiens OX=9606 GN=PPP3R1 PE=1 SV=2	PPP3R1	P63098	0.01	888208 1	-2
9606.ENSP00000 243346	N-myc-interactor OS=Homo sapiens OX=9606 GN=NMI PE=1 SV=2	NMI	Q1328 7	0.01	420103 3	-2
9606.ENSP00000 244230	U3 small nucleolar ribonucleoprotein protein MPP10 OS=Homo sapiens OX=9606 GN=MPHOSPH10 PE=1 SV=2	MPHOSP H10	O0056 6	0.01	189106 0	-2
9606.ENSP00000 244534	Histone H1.3 OS=Homo sapiens OX=9606 GN=H1-3 PE=1 SV=2	HIST1H1 D	P16402	0.01	4.25E+ 08	-2
9606.ENSP00000 245552	5'(3')-deoxyribonucleotidase, cytosolic type OS=Homo sapiens OX=9606 GN=NT5C PE=1 SV=2	NT5C	Q8TC D5	0.01	392912 3	-2
9606.ENSP00000 245816	ATP-dependent Clp protease proteolytic subunit, mitochondrial OS=Homo sapiens OX=9606 GN=CLPP PE=1 SV=1	CLPP	Q1674 0	0.01	290010 4	-2
9606.ENSP00000 245932	Vasodilator-stimulated phosphoprotein OS=Homo sapiens OX=9606 GN=VASP PE=1 SV=3	VASP	P50552	0.01	187164 2	-2
9606.ENSP00000 249071	Ras-related C3 botulinum toxin substrate 2 OS=Homo sapiens OX=9606 GN=RAC2 PE=1 SV=1	RAC2	P15153	0.01	323342 4	-2
9606.ENSP00000 250495	NEDD8 OS=Homo sapiens OX=9606 GN=NEDD8 PE=1 SV=1	NEDD8	Q1584 3	0.01	1.20E+ 07	-2
9606.ENSP00000 251038	Zinc finger CCCH domain-containing protein 14 OS=Homo sapiens OX=9606 GN=ZC3H14 PE=1 SV=1	ZC3H14	Q6PJT 7	0.01	302709 3	-2
9606.ENSP00000 251775	Sorting nexin-4 OS=Homo sapiens OX=9606 GN=SNX4 PE=1 SV=1	SNX4	O9521 9	0.01	192526 5	-2
9606.ENSP00000	Keratin, type II cytoskeletal 6B	KRT6B	P04259	0.01	4.69E+	-2

252252	OS=Homo sapiens OX=9606 GN=KRT6B PE=1 SV=5				07	
9606.ENSP00000254480	SWI/SNF complex subunit SMARCC1 OS=Homo sapiens OX=9606 GN=SMARCC1 PE=1 SV=3	SMARCC1	Q92922	0.01	2925490	-2
9606.ENSP00000255784	Coiled-coil domain-containing protein 134 OS=Homo sapiens OX=9606 GN=CCDC134 PE=1 SV=1	CCDC134	Q9H6E4	0.01	1923160	-2
9606.ENSP00000256441	Alpha-ketoglutarate dehydrogenase component 4 OS=Homo sapiens OX=9606 GN=MRPS36 PE=1 SV=2	MRPS36	P82909	0.01	6915166	-2
9606.ENSP00000259339	Torsin-1B OS=Homo sapiens OX=9606 GN=TOR1B PE=1 SV=2	TOR1B	O14657	0.01	4994160	-2
9606.ENSP00000260102	39S ribosomal protein L15, mitochondrial OS=Homo sapiens OX=9606 GN=MRPL15 PE=1 SV=1	MRPL15	Q9P015	0.01	5064907	-2
9606.ENSP00000260970	Peptidyl-prolyl cis-trans isomerase G OS=Homo sapiens OX=9606 GN=PPIG PE=1 SV=2	PPIG	Q13427	0.01	8766809	-2
9606.ENSP00000261245	CDK-activating kinase assembly factor MAT1 OS=Homo sapiens OX=9606 GN=MNAT1 PE=1 SV=1	MNAT1	P51948	0.01	2734037	-2
9606.ENSP00000261326	Molybdenum cofactor sulfurase OS=Homo sapiens OX=9606 GN=MOCOS PE=1 SV=2	MOCOS	Q96EN8	0.01	2533219	-2
9606.ENSP00000262160	Mothers against decapentaplegic homolog 2 OS=Homo sapiens OX=9606 GN=SMAD2 PE=1 SV=1	SMAD2	Q15796	0.01	9396173	-2
9606.ENSP00000262265	PIH1 domain-containing protein 1 OS=Homo sapiens OX=9606 GN=PIH1D1 PE=1 SV=1	PIH1D1	Q9NWS0	0.01	3810983	-2
9606.ENSP00000263212	Protein phosphatase 1F OS=Homo sapiens OX=9606 GN=PPM1F PE=1 SV=3	PPM1F	P49593	0.01	1690656	-2
9606.ENSP00000263382	Histone chaperone ASF1B OS=Homo sapiens OX=9606 GN=ASF1B PE=1 SV=1	ASF1B	Q9NVP2	0.01	1318087	-2
9606.ENSP00000263697	DnaJ homolog subfamily C member 8 OS=Homo sapiens OX=9606 GN=DNAJC8 PE=1 SV=2	DNAJC8	O75937	0.01	8754047	-2
9606.ENSP00000264036	Cell surface glycoprotein MUC18 OS=Homo sapiens OX=9606 GN=MCAM PE=1 SV=2	MCAM	P43121	0.01	5429542	-2
9606.ENSP00000264255	Thioredoxin domain-containing protein 9 OS=Homo sapiens OX=9606 GN=TXNDC9 PE=1 SV=2	TXNDC9	O14530	0.01	2830627	-2
9606.ENSP00000264883	Nucleoporin p54 OS=Homo sapiens OX=9606 GN=NUP54	NUP54	Q7Z3B4	0.01	6293924	-2

	PE=1 SV=2					
9606.ENSP00000 265097	THO complex subunit 3 OS=Homo sapiens OX=9606 GN=THOC3 PE=1 SV=1	THOC3	Q96J0 1	0.01	441472 6	-2
9606.ENSP00000 265562	Tyrosine-protein phosphatase non-receptor type 23 OS=Homo sapiens OX=9606 GN=PTPN23 PE=1 SV=1	PTPN23	Q9H3S 7	0.01	2.14E+ 07	-2
9606.ENSP00000 266069	Glucose-induced degradation protein 8 homolog OS=Homo sapiens OX=9606 GN=GID8 PE=1 SV=1	GID8	Q9NW U2	0.01	238825 1	-2
9606.ENSP00000 266604	Protein LLP homolog OS=Homo sapiens OX=9606 GN=LLPH PE=1 SV=1	LLPH	Q9BR T6	0.01	497982 3	-2
9606.ENSP00000 267812	Microfibrillar-associated protein 1 OS=Homo sapiens OX=9606 GN=MFAP1 PE=1 SV=2	MFAP1	P55081	0.01	256795 1	-2
9606.ENSP00000 268097	Beta-hexosaminidase subunit alpha OS=Homo sapiens OX=9606 GN=HEXA PE=1 SV=2	HEXA	P06865	0.01	399401 9	-2
9606.ENSP00000 268261	Phosphomannomutase 2 OS=Homo sapiens OX=9606 GN=PMM2 PE=1 SV=1	PMM2	O1530 5	0.01	1.34E+ 07	-2
9606.ENSP00000 268802	RNA-binding protein NOB1 OS=Homo sapiens OX=9606 GN=NOB1 PE=1 SV=1	NOB1	Q9UL X3	0.01	271742 1	-2
9606.ENSP00000 273541	Pre-mRNA-splicing factor ISY1 homolog OS=Homo sapiens OX=9606 GN=ISY1 PE=1 SV=3	ISY1	Q9UL R0	0.01	1.76E+ 07	-2
9606.ENSP00000 274311	Protein pelota homolog OS=Homo sapiens OX=9606 GN=PELO PE=1 SV=2	PELO	Q9BR X2	0.01	581159 5	-2
9606.ENSP00000 278483	Protein Hikeshi OS=Homo sapiens OX=9606 GN=HIKESHI PE=1 SV=2	C11orf73	Q53FT 3	0.01	240942 4	-2
9606.ENSP00000 282074	Kinetochore protein Spc25 OS=Homo sapiens OX=9606 GN=SPC25 PE=1 SV=1	SPC25	Q9HB M1	0.01	169119 1	-2
9606.ENSP00000 283875	General transcription factor IIE subunit 1 OS=Homo sapiens OX=9606 GN=GTF2E1 PE=1 SV=2	GTF2E1	P29083	0.01	285691 2	-2
9606.ENSP00000 286175	Peptidyl-prolyl cis-trans isomerase-like 3 OS=Homo sapiens OX=9606 GN=PPIL3 PE=1 SV=1	PPIL3	Q9H2 H8	0.01	1.31E+ 07	-2
9606.ENSP00000 288071	ATP-dependent RNA helicase DDX19B OS=Homo sapiens OX=9606 GN=DDX19B PE=1 SV=1	DDX19B	Q9UM R2	0.01	2.78E+ 07	-2
9606.ENSP00000 288937	39S ribosomal protein L17, mitochondrial OS=Homo sapiens OX=9606 GN=MRPL17 PE=1 SV=1	MRPL17	Q9NR X2	0.01	324881 1	-2
9606.ENSP00000	Nuclear receptor coactivator 5	NCOA5	Q9HC	0.01	369137	-2

290231	OS=Homo sapiens OX=9606 GN=NCOA5 PE=1 SV=2		D5		6	
9606.ENSP00000 292476	Cleavage and polyadenylation specificity factor subunit 4 OS=Homo sapiens OX=9606 GN=CPSF4 PE=1 SV=1	CPSF4	O9563 9	0.01	200062 7	-2
9606.ENSP00000 295119	Nucleoporin NUP35 OS=Homo sapiens OX=9606 GN=NUP35 PE=1 SV=1	NUP35	Q8NF H5	0.01	746460 0	-2
9606.ENSP00000 295633	Follistatin-related protein 1 OS=Homo sapiens OX=9606 GN=FSTL1 PE=1 SV=1	FSTL1	Q1284 1	0.01	558844 1	-2
9606.ENSP00000 295767	Mitochondrial intermembrane space import and assembly protein 40 OS=Homo sapiens OX=9606 GN=CHCHD4 PE=1 SV=1	CHCHD4	Q8N4 Q1	0.01	476287 4	-2
9606.ENSP00000 299163	Hypoxia-inducible factor 1-alpha inhibitor OS=Homo sapiens OX=9606 GN=HIF1AN PE=1 SV=2	HIF1AN	Q9NW T6	0.01	342460 0	-2
9606.ENSP00000 300249	Microtubule-associated protein RP/EB family member 2 OS=Homo sapiens OX=9606 GN=MAPRE2 PE=1 SV=1	MAPRE2	Q1555 5	0.01	530181 5	-2
9606.ENSP00000 300935	Ras-related protein Rab-8A OS=Homo sapiens OX=9606 GN=RAB8A PE=1 SV=1	RAB8A	P61006	0.01	836761 3	-2
9606.ENSP00000 301458	CD320 antigen OS=Homo sapiens OX=9606 GN=CD320 PE=1 SV=1	CD320	Q9NPF 0	0.01	712255 3	-2
9606.ENSP00000 302037	Mitochondrial fission factor OS=Homo sapiens OX=9606 GN=MFF PE=1 SV=1	MFF	Q9GZ Y8	0.01	248139 5	-2
9606.ENSP00000 303423	Protein farnesyltransferase/geranylgeranyltransferase type-1 subunit alpha OS=Homo sapiens OX=9606 GN=FNTA PE=1 SV=1	FNTA	P49354	0.01	510064 4	-2
9606.ENSP00000 306920	Beta-galactosidase OS=Homo sapiens OX=9606 GN=GLB1 PE=1 SV=2	GLB1	P16278	0.01	1.17E+ 07	-2
9606.ENSP00000 309430	GPI transamidase component PIG-S OS=Homo sapiens OX=9606 GN=PIGS PE=1 SV=3	PIGS	Q96S5 2	0.01	300885 4	-2
9606.ENSP00000 310042	Cytoplasmic 60S subunit biogenesis factor ZNF622 OS=Homo sapiens OX=9606 GN=ZNF622 PE=1 SV=1	ZNF622	Q969S 3	0.01	183409 0	-2
9606.ENSP00000 310596	U6 snRNA-associated Sm-like protein LSm1 OS=Homo sapiens OX=9606 GN=LSM1 PE=1 SV=1	LSM1	O1511 6	0.01	463663 9	-2
9606.ENSP00000 310901	Cysteine and glycine-rich protein 2 OS=Homo sapiens OX=9606 GN=CSRP2 PE=1 SV=3	CSRP2	Q1652 7	0.01	1.40E+ 07	-2

9606.ENSP00000313422	ADP-ribosylation factor-like protein 6-interacting protein 4 OS=Homo sapiens OX=9606 GN=ARL6IP4 PE=1 SV=3	ARL6IP4	Q66PJ3	0.01	5468884	-2
9606.ENSP00000315568	Late secretory pathway protein AVL9 homolog OS=Homo sapiens OX=9606 GN=AVL9 PE=1 SV=1	AVL9	Q8NBF6	0.01	7565934	-2
9606.ENSP00000316598	Putative GTP-binding protein 6 OS=Homo sapiens OX=9606 GN=GTPBP6 PE=1 SV=4	GTPBP6	O43824	0.01	1182028	-2
9606.ENSP00000318352	MORF4 family-associated protein 1 OS=Homo sapiens OX=9606 GN=MRFAP1 PE=1 SV=1	MRFAP1	Q9Y605	0.01	7798498	-2
9606.ENSP00000320917	Protein KRI1 homolog OS=Homo sapiens OX=9606 GN=KRI1 PE=1 SV=3	KRI1	Q8N9T8	0.01	2719869	-2
9606.ENSP00000321464	Uncharacterized protein C10orf67, mitochondrial OS=Homo sapiens OX=9606 GN=C10orf67 PE=1 SV=3	C10orf67	Q8IYJ2	0.01	6908512	-2
9606.ENSP00000321606	Dihydropyrimidinase-related protein 1 OS=Homo sapiens OX=9606 GN=CRMP1 PE=1 SV=1	CRMP1	Q14194	0.01	5200224	-2
9606.ENSP00000324205	Charged multivesicular body protein 4a OS=Homo sapiens OX=9606 GN=CHMP4A PE=1 SV=3	CHMP4A	Q9BY43	0.01	5601793	-2
9606.ENSP00000325146	Collagen alpha-1(XII) chain OS=Homo sapiens OX=9606 GN=COL12A1 PE=1 SV=2	COL12A1	Q99715	0.01	2430492	-2
9606.ENSP00000326531	THO complex subunit 6 homolog OS=Homo sapiens OX=9606 GN=THOC6 PE=1 SV=1	THOC6	Q86W42	0.01	6878463	-2
9606.ENSP00000326806	Nuclear cap-binding protein subunit 2 OS=Homo sapiens OX=9606 GN=NCBP2 PE=1 SV=1	NCBP2	P52298	0.01	1.33E+07	-2
9606.ENSP00000328570	Glutaredoxin-related protein 5, mitochondrial OS=Homo sapiens OX=9606 GN=GLRX5 PE=1 SV=2	GLRX5	Q86SX6	0.01	1430772	-2
9606.ENSP00000328773	Protein HEXIM1 OS=Homo sapiens OX=9606 GN=HEXIM1 PE=1 SV=1	HEXIM1	O94992	0.01	3325312	-2
9606.ENSP00000332194	Histone H2A type 2-C OS=Homo sapiens OX=9606 GN=H2AC20 PE=1 SV=4	HIST2H2AC	Q16777	0.01	1.27E+09	-2
9606.ENSP00000332790	Histone H2A type 2-B OS=Homo sapiens OX=9606 GN=H2AC21 PE=1 SV=3	HIST2H2AB	Q8IUE6	0.01	1.44E+08	-2
9606.ENSP00000333551	Pyridoxal phosphate homeostasis protein OS=Homo sapiens OX=9606 GN=PLPBP PE=1 SV=1	PROSC	O94903	0.01	5716022	-2
9606.ENSP00000	Nexilin OS=Homo sapiens	NEXN	Q0ZG	0.01	148519	-2

333938	OX=9606 GN=NEXN PE=1 SV=1		T2		3	
9606.ENSP00000 335636	N-alpha-acetyltransferase 20 OS=Homo sapiens OX=9606 GN=NAA20 PE=1 SV=1	NAA20	P61599	0.01	632466 3	-2
9606.ENSP00000 338258	Cdc42 effector protein 4 OS=Homo sapiens OX=9606 GN=CDC42EP4 PE=1 SV=1	CDC42E P4	Q9H3 Q1	0.01	545473 9	-2
9606.ENSP00000 339245	YTH domain-containing protein 1 OS=Homo sapiens OX=9606 GN=YTHDC1 PE=1 SV=3	YTHDC1	Q96M U7	0.01	421996 9	-2
9606.ENSP00000 339328	Urokinase plasminogen activator surface receptor OS=Homo sapiens OX=9606 GN=PLAUR PE=1 SV=1	PLAUR	Q0340 5	0.01	697291 4	-2
9606.ENSP00000 340305	Ubiquitin-conjugating enzyme E2 variant 1 OS=Homo sapiens OX=9606 GN=UBE2V1 PE=1 SV=2	UBE2V1	Q1340 4	0.01	1.36E+ 08	-2
9606.ENSP00000 340736	Glycogenin-1 OS=Homo sapiens OX=9606 GN=GYG1 PE=1 SV=4	GYG1	P46976	0.01	1.33E+ 07	-2
9606.ENSP00000 340796	DBIRD complex subunit ZNF326 OS=Homo sapiens OX=9606 GN=ZNF326 PE=1 SV=2	ZNF326	Q5BK Z1	0.01	847362 2	-2
9606.ENSP00000 343027	Guanine nucleotide-binding protein G(i) subunit alpha-1 OS=Homo sapiens OX=9606 GN=GNAI1 PE=1 SV=2	GNAI1	P63096	0.01	698556 6	-2
9606.ENSP00000 346012	60S ribosomal protein L36a-like OS=Homo sapiens OX=9606 GN=RPL36AL PE=1 SV=3	RPL36A L	Q969Q 0	0.01	1.02E+ 08	-2
9606.ENSP00000 347464	ARF GTPase-activating protein GIT2 OS=Homo sapiens OX=9606 GN=GIT2 PE=1 SV=2	GIT2	Q1416 1	0.01	3.83E+ 07	-2
9606.ENSP00000 347836	Ubiquitin-conjugating enzyme E2 H OS=Homo sapiens OX=9606 GN=UBE2H PE=1 SV=1	UBE2H	P62256	0.01	1.39E+ 07	-2
9606.ENSP00000 348722	Pseudouridylate synthase 7 homolog OS=Homo sapiens OX=9606 GN=PUS7 PE=1 SV=2	PUS7	Q96PZ 0	0.01	164212 3	-2
9606.ENSP00000 349351	Protein bicaudal D homolog 2 OS=Homo sapiens OX=9606 GN=BICD2 PE=1 SV=1	BICD2	Q8TD1 6	0.01	993732 8	-2
9606.ENSP00000 351832	Rab3 GTPase-activating protein non-catalytic subunit OS=Homo sapiens OX=9606 GN=RAB3GAP2 PE=1 SV=1	RAB3GA P2	Q9H2 M9	0.01	803125 3	-2
9606.ENSP00000 352516	DNA (cytosine-5)-methyltransferase 1 OS=Homo sapiens OX=9606 GN=DNMT1 PE=1 SV=2	DNMT1	P26358	0.01	1.06E+ 07	-2
9606.ENSP00000 353463	Transcriptional repressor p66-alpha OS=Homo sapiens OX=9606 GN=GATAD2A	GATAD2 A	Q86YP 4	0.01	1.17E+ 07	-2

	PE=1 SV=1					
9606.ENSP00000355013	RNA polymerase-associated protein CTR9 homolog OS=Homo sapiens OX=9606 GN=CTR9 PE=1 SV=1	CTR9	Q6PD62	0.01	1902293	-2
9606.ENSP00000355572	Cytochrome c oxidase assembly factor 6 homolog OS=Homo sapiens OX=9606 GN=COA6 PE=1 SV=1	COA6	Q5JTJ3	0.01	5902537	-2
9606.ENSP00000355605	Exocyst complex component 8 OS=Homo sapiens OX=9606 GN=EXOC8 PE=1 SV=2	EXOC8	Q8IYI6	0.01	7.41E+07	-2
9606.ENSP00000355809	Protein enabled homolog OS=Homo sapiens OX=9606 GN=ENAH PE=1 SV=2	ENAH	Q8N8S7	0.01	1.01E+07	-2
9606.ENSP00000355955	Neudesin OS=Homo sapiens OX=9606 GN=NENF PE=1 SV=1	NENF	Q9UMX5	0.01	9889796	-2
9606.ENSP00000356480	E3 ubiquitin-protein ligase RING2 OS=Homo sapiens OX=9606 GN=RNF2 PE=1 SV=1	RNF2	Q99496	0.01	7328653	-2
9606.ENSP00000356982	Ubiquitin-fold modifier-conjugating enzyme 1 OS=Homo sapiens OX=9606 GN=UFC1 PE=1 SV=3	UFC1	Q9Y3C8	0.01	4257600	-2
9606.ENSP00000357384	Dolichol-phosphate mannosyltransferase subunit 3 OS=Homo sapiens OX=9606 GN=DPM3 PE=1 SV=2	DPM3	Q9P2X0	0.01	3.04E+07	-2
9606.ENSP00000358548	GTPase NRas OS=Homo sapiens OX=9606 GN=NRAS PE=1 SV=1	NRAS	P01111	0.01	6810219	-2
9606.ENSP00000358635	Heterogeneous nuclear ribonucleoprotein Q OS=Homo sapiens OX=9606 GN=SYNCRIP PE=1 SV=2	SYNCRIP	O60506	0.01	8.50E+07	-2
9606.ENSP00000358674	Ubiquitin-like protein 4A OS=Homo sapiens OX=9606 GN=UBL4A PE=1 SV=1	UBL4A	P11441	0.01	1.09E+07	-2
9606.ENSP00000358799	RNA-binding protein 15 OS=Homo sapiens OX=9606 GN=RBM15 PE=1 SV=2	RBM15	Q96T37	0.01	6111022	-2
9606.ENSP00000360497	E3 ubiquitin-protein ligase RNF113A OS=Homo sapiens OX=9606 GN=RNF113A PE=1 SV=1	RNF113A	O15541	0.01	2275200	-2
9606.ENSP00000360613	Ubiquitin-conjugating enzyme E2 A OS=Homo sapiens OX=9606 GN=UBE2A PE=1 SV=2	UBE2A	P49459	0.01	9067725	-2
9606.ENSP00000360642	Coiled-coil and C2 domain-containing protein 1B OS=Homo sapiens OX=9606 GN=CC2D1B PE=1 SV=1	CC2D1B	Q5T0F9	0.01	1399815	-2
9606.ENSP00000360727	Rab-like protein 6 OS=Homo sapiens OX=9606 GN=RABL6 PE=1 SV=2	RABL6	Q3YEC7	0.01	2362301	-2
9606.ENSP00000	Surfeit locus protein 6	SURF6	O7568	0.01	561704	-2

361092	OS=Homo sapiens OX=9606 GN=SURF6 PE=1 SV=3		3		9	
9606.ENSP00000 361162	Target of EGR1 protein 1 OS=Homo sapiens OX=9606 GN=TOE1 PE=1 SV=1	TOE1	Q96G M8	0.01	711389 4	-2
9606.ENSP00000 361524	Splicing factor C9orf78 OS=Homo sapiens OX=9606 GN=C9orf78 PE=1 SV=1	C9orf78	Q9NZ6 3	0.01	357997 8	-2
9606.ENSP00000 362308	Adapter SH3BGRL OS=Homo sapiens OX=9606 GN=SH3BGRL PE=1 SV=1	SH3BGR L	O7536 8	0.01	4.86E+ 07	-2
9606.ENSP00000 363019	Ubiquitin-conjugating enzyme E2 D1 OS=Homo sapiens OX=9606 GN=UBE2D1 PE=1 SV=1	UBE2D1	P51668	0.01	608006 5	-2
9606.ENSP00000 363286	DNA polymerase epsilon subunit 3 OS=Homo sapiens OX=9606 GN=POLE3 PE=1 SV=1	POLE3	Q9NR F9	0.01	1.48E+ 07	-2
9606.ENSP00000 366179	DnaJ homolog subfamily C member 1 OS=Homo sapiens OX=9606 GN=DNAJC1 PE=1 SV=1	DNAJC1	Q96K C8	0.01	1.22E+ 07	-2
9606.ENSP00000 367028	Treacle protein OS=Homo sapiens OX=9606 GN=TCOF1 PE=1 SV=3	TCOF1	Q1342 8	0.01	9.06E+ 07	-2
9606.ENSP00000 367440	Tyrosyl-DNA phosphodiesterase 2 OS=Homo sapiens OX=9606 GN=TDP2 PE=1 SV=1	TDP2	O9555 1	0.01	185837 4	-2
9606.ENSP00000 369055	Beta-1,4-galactosyltransferase 1 OS=Homo sapiens OX=9606 GN=B4GALT1 PE=1 SV=5	B4GALT 1	P15291	0.01	345984 8	-2
9606.ENSP00000 369897	Cysteine--tRNA ligase, cytoplasmic OS=Homo sapiens OX=9606 GN=CARS1 PE=1 SV=3	CARS	P49589	0.01	6.69E+ 07	-2
9606.ENSP00000 370222	Proteasome maturation protein OS=Homo sapiens OX=9606 GN=POMP PE=1 SV=1	POMP	Q9Y24 4	0.01	881463 0	-2
9606.ENSP00000 371155	Nucleoporin p58/p45 OS=Homo sapiens OX=9606 GN=NUP58 PE=1 SV=1	NUPL1	Q9BV L2	0.01	572958 7	-2
9606.ENSP00000 373772	E3 ubiquitin-protein ligase BRE1A OS=Homo sapiens OX=9606 GN=RNF20 PE=1 SV=2	RNF20	Q5VT R2	0.01	2.12E+ 07	-2
9606.ENSP00000 374280	RNA polymerase-associated protein RTF1 homolog OS=Homo sapiens OX=9606 GN=RTF1 PE=1 SV=4	RTF1	Q9254 1	0.01	393699 5	-2
9606.ENSP00000 375777	Striatin-4 OS=Homo sapiens OX=9606 GN=STRN4 PE=1 SV=2	STRN4	Q9NR L3	0.01	249460 5	-2
9606.ENSP00000 377040	Alpha-aminoadipic semialdehyde synthase, mitochondrial OS=Homo sapiens OX=9606 GN=AASS PE=1 SV=1	AASS	Q9UD R5	0.01	202323 7	-2

9606.ENSP00000377262	SRSF protein kinase 2 OS=Homo sapiens OX=9606 GN=SRPK2 PE=1 SV=3	SRPK2	P78362	0.01	3437387	-2
9606.ENSP00000377298	Nuclear mitotic apparatus protein 1 OS=Homo sapiens OX=9606 GN=NUMA1 PE=1 SV=2	NUMA1	Q14980	0.01	2.80E+07	-2
9606.ENSP00000377486	39S ribosomal protein L19, mitochondrial OS=Homo sapiens OX=9606 GN=MRPL19 PE=1 SV=2	MRPL19	P49406	0.01	4652532	-2
9606.ENSP00000379217	Ubiquitin domain-containing protein UBFD1 OS=Homo sapiens OX=9606 GN=UBFD1 PE=1 SV=2	UBFD1	O14562	0.01	5287985	-2
9606.ENSP00000379601	Ras-related protein Rab-27A OS=Homo sapiens OX=9606 GN=RAB27A PE=1 SV=3	RAB27A	P51159	0.01	6271154	-2
9606.ENSP00000379612	Tax1-binding protein 1 OS=Homo sapiens OX=9606 GN=TAX1BP1 PE=1 SV=2	TAX1BP1	Q86VP1	0.01	6026819	-2
9606.ENSP00000380178	Ubiquitin-conjugating enzyme E2 G1 OS=Homo sapiens OX=9606 GN=UBE2G1 PE=1 SV=3	UBE2G1	P62253	0.01	5.99E+07	-2
9606.ENSP00000380557	A-kinase anchor protein 8-like OS=Homo sapiens OX=9606 GN=AKAP8L PE=1 SV=4	AKAP8L	Q9ULX6	0.01	5231941	-2
9606.ENSP00000380998	Charged multivesicular body protein 1a OS=Homo sapiens OX=9606 GN=CHMP1A PE=1 SV=1	CHMP1A	Q9HD42	0.01	1.61E+07	-2
9606.ENSP00000381072	Serpin B8 OS=Homo sapiens OX=9606 GN=SERPINB8 PE=1 SV=2	SERPINB8	P50452	0.01	5556556	-2
9606.ENSP00000382707	Interferon-induced transmembrane protein 3 OS=Homo sapiens OX=9606 GN=IFITM3 PE=1 SV=2	IFITM3	Q01628	0.01	6152576	-2
9606.ENSP00000383866	SUZ domain-containing protein 1 OS=Homo sapiens OX=9606 GN=SZRD1 PE=1 SV=1	SZRD1	Q7Z422	0.01	4337575	-2
9606.ENSP00000383938	Protein disulfide isomerase CRELD2 OS=Homo sapiens OX=9606 GN=CRELD2 PE=1 SV=1	CRELD2	Q6UXH1	0.01	7342143	-2
9606.ENSP00000384474	Huntingtin-interacting protein K OS=Homo sapiens OX=9606 GN=HYPK PE=1 SV=3	HYPK	Q9NX55	0.01	1.84E+07	-2
9606.ENSP00000385749	cAMP-dependent protein kinase type I-beta regulatory subunit OS=Homo sapiens OX=9606 GN=PRKAR1B PE=1 SV=4	PRKAR1B	P31321	0.01	2001797	-2
9606.ENSP00000386458	Pre-mRNA-processing factor 40 homolog A OS=Homo sapiens OX=9606 GN=PRPF40A PE=1 SV=2	PRPF40A	O75400	0.01	3.08E+07	-2
9606.ENSP00000387006	Pre-mRNA-splicing factor CWC22 homolog OS=Homo	CWC22	Q9HCG8	0.01	3463055	-2

	sapiens OX=9606 GN=CWC22 PE=1 SV=3					
9606.ENSP00000387286	Ras-related protein Rab-1A OS=Homo sapiens OX=9606 GN=RAB1A PE=1 SV=3	RAB1A	P62820	0.01	9224928	-2
9606.ENSP00000387365	Periodic tryptophan protein 1 homolog OS=Homo sapiens OX=9606 GN=PWP1 PE=1 SV=1	PWP1	Q13610	0.01	1.02E+07	-2
9606.ENSP00000392043	Plasma membrane calcium-transporting ATPase 1 OS=Homo sapiens OX=9606 GN=ATP2B1 PE=1 SV=4	ATP2B1	P20020	0.01	8036718	-2
9606.ENSP00000395083	Arfaptin-1 OS=Homo sapiens OX=9606 GN=ARFIP1 PE=1 SV=2	ARFIP1	P53367	0.01	3.68E+07	-2
9606.ENSP00000395590	Interferon-induced 35 kDa protein OS=Homo sapiens OX=9606 GN=IFI35 PE=1 SV=5	IFI35	P80217	0.01	4905846	-2
9606.ENSP00000401645	Plasminogen activator inhibitor 2 OS=Homo sapiens OX=9606 GN=SERPINB2 PE=1 SV=2	SERPINB2	P05120	0.01	2336596	-2
9606.ENSP00000402060	Poliovirus receptor OS=Homo sapiens OX=9606 GN=PVR PE=1 SV=2	PVR	P15151	0.01	2771032	-2
9606.ENSP00000408695	Protein kinase C alpha type OS=Homo sapiens OX=9606 GN=PRKCA PE=1 SV=4	PRKCA	P17252	0.01	1895501	-2
9606.ENSP00000413156	Ras-related protein Rab-34 OS=Homo sapiens OX=9606 GN=RAB34 PE=1 SV=1	RAB34	Q9BZG1	0.01	4088582	-2
9606.ENSP00000413625	Formin-binding protein 1 OS=Homo sapiens OX=9606 GN=FNBP1 PE=1 SV=2	FNBP1	Q96RU3	0.01	3040814	-2
9606.ENSP00000415786	Glia-derived nexin OS=Homo sapiens OX=9606 GN=SERPINE2 PE=1 SV=1	SERPINE2	P07093	0.01	3014458	-2
9606.ENSP00000416255	Eukaryotic translation initiation factor 4 gamma 1 OS=Homo sapiens OX=9606 GN=EIF4G1 PE=1 SV=4	EIF4G1	Q04637	0.01	2.40E+08	-2
9606.ENSP00000419782	Cyclin-dependent kinase 5 OS=Homo sapiens OX=9606 GN=CDK5 PE=1 SV=3	CDK5	Q00535	0.01	1.19E+07	-2
9606.ENSP00000420176	DNA polymerase epsilon subunit 4 OS=Homo sapiens OX=9606 GN=POLE4 PE=1 SV=2	POLE4	Q9NR33	0.01	3522953	-2
9606.ENSP00000423563	Core histone macro-H2A.1 OS=Homo sapiens OX=9606 GN=MACROH2A1 PE=1 SV=5	H2AFY	O75367	0.01	9.45E+07	-2
9606.ENSP00000424417	Pleiotropic regulator 1 OS=Homo sapiens OX=9606 GN=PLRG1 PE=1 SV=1	PLRG1	O43660	0.01	3680617	-2
9606.ENSP00000425809	Phosphoacetylglucosamine mutase OS=Homo sapiens OX=9606 GN=PGM3 PE=1	PGM3	O95394	0.01	2137910	-2

	SV=1					
9606.ENSP00000 429986	Transcription and mRNA export factor ENY2 OS=Homo sapiens OX=9606 GN=ENY2 PE=1 SV=1	ENY2	Q9NP A8	0.01	1.06E+ 07	-2
9606.ENSP00000 432472	Protein BRICK1 OS=Homo sapiens OX=9606 GN=BRK1 PE=1 SV=1	BRK1	Q8WU W1	0.01	819294 8	-2
9606.ENSP00000 433919	Methylosome subunit pICln OS=Homo sapiens OX=9606 GN=CLNS1A PE=1 SV=1	CLNS1A	P54105	0.01	252915 8	-2
9606.ENSP00000 435460	Galectin-8 OS=Homo sapiens OX=9606 GN=LGALS8 PE=1 SV=4	LGALS8	O0021 4	0.01	222436 7	-2
9606.ENSP00000 438455	Mitochondrial import inner membrane translocase subunit Tim8 B OS=Homo sapiens OX=9606 GN=TIMM8B PE=1 SV=1	TIMM8B	Q9Y5J 9	0.01	492496 4	-2
9606.ENSP00000 459943	Rabankyrin-5 OS=Homo sapiens OX=9606 GN=ANKFY1 PE=1 SV=2	ANKFY1	Q9P2R 3	0.01	359404 8	-2
9606.ENSP00000 467141	Titin OS=Homo sapiens OX=9606 GN=TTN PE=1 SV=4	TTN	Q8WZ 42	0.01	3.01E+ 07	-2
9606.ENSP00000 467466	40S ribosomal protein S15 OS=Homo sapiens OX=9606 GN=RPS15 PE=1 SV=2	RPS15	P62841	0.01	3.45E+ 07	-2
9606.ENSP00000 471896	Interferon regulatory factor 3 OS=Homo sapiens OX=9606 GN=IRF3 PE=1 SV=1	IRF3	Q1465 3	0.01	322292 8	-2
9606.ENSP00000 475615	Spliceosome-associated protein CWC15 homolog OS=Homo sapiens OX=9606 GN=CWC15 PE=1 SV=2	CWC15	Q9P01 3	0.01	1.50E+ 07	-2
9606.ENSP00000 476117	WD repeat-containing protein 18 OS=Homo sapiens OX=9606 GN=WDR18 PE=1 SV=2	WDR18	Q9BV 38	0.01	246316 5	-2
9606.ENSP00000 477310	Dynein light chain 2, cytoplasmic OS=Homo sapiens OX=9606 GN=DYNLL2 PE=1 SV=1	DYNLL2	Q96FJ 2	0.01	4.92E+ 07	-2
9606.ENSP00000 477572	Heme oxygenase 2 OS=Homo sapiens OX=9606 GN=HMOX2 PE=1 SV=2	HMOX2	P30519	0.01	811533 0	-2
9606.ENSP00000 478927	Transcriptional coactivator YAP1 OS=Homo sapiens OX=9606 GN=YAP1 PE=1 SV=2	YAP1	P46937	0.01	473212 2	-2
9606.ENSP00000 479919	Endophilin-B1 OS=Homo sapiens OX=9606 GN=SH3GLB1 PE=1 SV=1	SH3GLB 1	Q9Y37 1	0.01	425564 3	-2
9606.ENSP00000 480332	Trafficking protein particle complex subunit 3 OS=Homo sapiens OX=9606 GN=TRAPPC3 PE=1 SV=1	TRAPPC 3	O4361 7	0.01	1.74E+ 07	-2
9606.ENSP00000 480832	Nucleolar protein 16 OS=Homo sapiens OX=9606 GN=NOP16 PE=1 SV=2	NOP16	Q9Y3 C1	0.01	1.31E+ 07	-2

9606.ENSP00000483345	Hepatoma-derived growth factor-related protein 2 OS=Homo sapiens OX=9606 GN=HDGFL2 PE=1 SV=1	HDGFRP2	Q7Z4V5	0.01	7442967	-2
9606.ENSP00000259632	Dynactin subunit 3 OS=Homo sapiens OX=9606 GN=DCTN3 PE=1 SV=1	DCTN3	O75935	0.014015	5768664	-1.85341
9606.ENSP00000477792	Leucine-rich repeat-containing protein 41 OS=Homo sapiens OX=9606 GN=LRRC41 PE=1 SV=3	LRRC41	Q15345	0.021316	1.38E+07	-1.6713
9606.ENSP00000340691	Eukaryotic translation initiation factor 4E-binding protein 1 OS=Homo sapiens OX=9606 GN=EIF4EBP1 PE=1 SV=3	EIF4EBP1	Q13541	0.120238	3384446	-0.91996
9606.ENSP00000365505	Proteolipid protein 2 OS=Homo sapiens OX=9606 GN=PLP2 PE=1 SV=1	PLP2	Q04941	0.120302	1.34E+07	-0.91973
9606.ENSP00000358554	Pre-mRNA-splicing factor SPF27 OS=Homo sapiens OX=9606 GN=BCAS2 PE=1 SV=1	BCAS2	O75934	0.141318	1792359	-0.8498
9606.ENSP00000238497	Vacuolar protein sorting-associated protein 4B OS=Homo sapiens OX=9606 GN=VPS4B PE=1 SV=2	VPS4B	O75351	0.142835	4109307	-0.84517
9606.ENSP00000414398	DnaJ homolog subfamily B member 11 OS=Homo sapiens OX=9606 GN=DNAJB11 PE=1 SV=1	DNAJB11	Q9UBS4	0.161953	4516957	-0.79061
9606.ENSP00000362592	Histone-binding protein RBBP4 OS=Homo sapiens OX=9606 GN=RBBP4 PE=1 SV=3	RBBP4	Q09028	0.164907	5.07E+07	-0.78276
9606.ENSP00000356243	Ubiquitin-conjugating enzyme E2 T OS=Homo sapiens OX=9606 GN=UBE2T PE=1 SV=1	UBE2T	Q9NPD8	0.167243	1.33E+07	-0.77665
9606.ENSP00000329419	Coatomer subunit beta' OS=Homo sapiens OX=9606 GN=COPB2 PE=1 SV=2	COPB2	P35606	0.203182	4.14E+08	-0.69212
9606.ENSP00000362711	Alpha-taxilin OS=Homo sapiens OX=9606 GN=TXLNA PE=1 SV=3	TXLNA	P40222	0.222732	2.68E+07	-0.65222
9606.ENSP00000230431	2'-deoxynucleoside 5'-phosphate N-hydrolase 1 OS=Homo sapiens OX=9606 GN=DNPH1 PE=1 SV=1	DNPH1	O43598	0.224143	4742473	-0.64947
9606.ENSP00000225726	PAT complex subunit CCDC47 OS=Homo sapiens OX=9606 GN=CCDC47 PE=1 SV=1	CCDC47	Q96A33	0.232682	5.15E+07	-0.63324
9606.ENSP00000292211	Ubiquitin-conjugating enzyme E2 Q1 OS=Homo sapiens OX=9606 GN=UBE2Q1 PE=1 SV=1	UBE2Q1	Q7Z7E8	0.235481	6013445	-0.62804
9606.ENSP00000366819	Ubiquitin carboxyl-terminal hydrolase isozyme L3 OS=Homo sapiens OX=9606 GN=UCHL3 PE=1 SV=1	UCHL3	P15374	0.266333	1.86E+07	-0.57458
9606.ENSP00000	Adenosine kinase OS=Homo	ADK	P55263	0.268762	492855	-

286621	sapiens OX=9606 GN=ADK PE=1 SV=2				2	0.57063
9606.ENSP00000248450	Angio-associated migratory cell protein OS=Homo sapiens OX=9606 GN=AAMP PE=1 SV=2	AAMP	Q13685	0.271195	2609640	-0.56672
9606.ENSP00000387282	Arf-GAP domain and FG repeat-containing protein 1 OS=Homo sapiens OX=9606 GN=AGFG1 PE=1 SV=2	AGFG1	P52594	0.278691	3301000	-0.55488
9606.ENSP00000304854	NudC domain-containing protein 2 OS=Homo sapiens OX=9606 GN=NUDCD2 PE=1 SV=1	NUDCD2	Q8WVJ2	0.29315	3.50E+07	-0.53291
9606.ENSP00000271628	Splicing factor 3B subunit 4 OS=Homo sapiens OX=9606 GN=SF3B4 PE=1 SV=1	SF3B4	Q15427	0.295913	6553051	-0.52884
9606.ENSP00000297071	Transformer-2 protein homolog alpha OS=Homo sapiens OX=9606 GN=TRA2A PE=1 SV=1	TRA2A	Q13595	0.298579	6558616	-0.52494
9606.ENSP00000248098	Jupiter microtubule associated homolog 2 OS=Homo sapiens OX=9606 GN=JPT2 PE=1 SV=1	HN1L	Q9H910	0.308483	1.56E+07	-0.51077
9606.ENSP00000430598	Copper transport protein ATOX1 OS=Homo sapiens OX=9606 GN=ATOX1 PE=1 SV=1	ATOX1	O00244	0.308877	9864927	-0.51021
9606.ENSP00000366565	Vacuolar protein sorting-associated protein 28 homolog OS=Homo sapiens OX=9606 GN=VPS28 PE=1 SV=1	VPS28	Q9UK41	0.31382	3428790	-0.50332
9606.ENSP00000361918	Peptidyl-prolyl cis-trans isomerase E OS=Homo sapiens OX=9606 GN=PPIE PE=1 SV=1	PPIE	Q9UNP9	0.318572	1.33E+07	-0.49679
9606.ENSP00000278193	Protein lin-7 homolog C OS=Homo sapiens OX=9606 GN=LIN7C PE=1 SV=1	LIN7C	Q9NUP9	0.320191	2.96E+07	-0.49459
9606.ENSP00000416951	Zinc finger CCCH domain-containing protein 18 OS=Homo sapiens OX=9606 GN=ZC3H18 PE=1 SV=2	ZC3H18	Q86VM9	0.322255	3562841	-0.4918
9606.ENSP00000368831	Sorting nexin-2 OS=Homo sapiens OX=9606 GN=SNX2 PE=1 SV=2	SNX2	O60749	0.325308	1.05E+07	-0.48771
9606.ENSP00000359939	Exosome complex component CSL4 OS=Homo sapiens OX=9606 GN=EXOSC1 PE=1 SV=1	EXOSC1	Q9Y3B2	0.336493	2642401	-0.47302
9606.ENSP00000247665	14 kDa phosphohistidine phosphatase OS=Homo sapiens OX=9606 GN=PHPT1 PE=1 SV=1	PHPT1	Q9NRX4	0.336728	4467288	-0.47272
9606.ENSP00000304903	CD2 antigen cytoplasmic tail-binding protein 2 OS=Homo sapiens OX=9606 GN=CD2BP2 PE=1 SV=1	CD2BP2	O95400	0.344887	1.10E+07	-0.46232

9606.ENSP00000262710	Apoptotic chromatin condensation inducer in the nucleus OS=Homo sapiens OX=9606 GN=ACIN1 PE=1 SV=2	ACIN1	Q9UKV3	0.351385	3.68E+07	-0.45422
9606.ENSP00000000233	ADP-ribosylation factor 5 OS=Homo sapiens OX=9606 GN=ARF5 PE=1 SV=2	ARF5	P84085	0.366012	2.07E+07	-0.4365
9606.ENSP00000357189	Hepatoma-derived growth factor OS=Homo sapiens OX=9606 GN=HDGF PE=1 SV=1	HDGF	P51858	0.370921	1.08E+08	-0.43072
9606.ENSP00000308546	7SK snRNA methylphosphate capping enzyme OS=Homo sapiens OX=9606 GN=MEPCE PE=1 SV=1	MEPCE	Q7L2J0	0.375793	3483017	-0.42505
9606.ENSP00000296490	WD repeat-containing protein 82 OS=Homo sapiens OX=9606 GN=WDR82 PE=1 SV=1	WDR82	Q6UXN9	0.4073	5725498	-0.39009
9606.ENSP00000258198	Cytoplasmic dynein 1 light intermediate chain 2 OS=Homo sapiens OX=9606 GN=DYNC1LI2 PE=1 SV=1	DYNC1LI2	O43237	0.408834	4.59E+07	-0.38845
9606.ENSP00000356056	Dynein light chain Tctex-type 1 OS=Homo sapiens OX=9606 GN=DYNLT1 PE=1 SV=1	DYNLT1	P63172	0.409779	3389683	-0.38745
9606.ENSP00000227322	Zinc finger protein ZPR1 OS=Homo sapiens OX=9606 GN=ZPR1 PE=1 SV=1	ZPR1	O75312	0.414618	1.39E+07	-0.38235
9606.ENSP00000363827	Basement membrane-specific heparan sulfate proteoglycan core protein OS=Homo sapiens OX=9606 GN=HSPG2 PE=1 SV=4	HSPG2	P98160	0.416281	1.45E+08	-0.38061
9606.ENSP00000265872	Cell division cycle and apoptosis regulator protein 1 OS=Homo sapiens OX=9606 GN=CCAR1 PE=1 SV=2	CCAR1	Q8IX12	0.437973	2.00E+07	-0.35855
9606.ENSP00000416534	Probable ATP-dependent RNA helicase DDX46 OS=Homo sapiens OX=9606 GN=DDX46 PE=1 SV=2	DDX46	Q7L014	0.439688	3.31E+07	-0.35686
9606.ENSP00000260118	Gamma-glutamyl hydrolase OS=Homo sapiens OX=9606 GN=GGH PE=1 SV=2	GGH	Q92820	0.441747	6748743	-0.35483
9606.ENSP00000406209	Epsin-1 OS=Homo sapiens OX=9606 GN=EPN1 PE=1 SV=2	EPN1	Q9Y6I3	0.445728	9897741	-0.35093
9606.ENSP00000367208	Protein SGT1 homolog OS=Homo sapiens OX=9606 GN=SUGT1 PE=1 SV=3	SUGT1	Q9Y2Z0	0.448839	9998526	-0.34791
9606.ENSP00000377424	Equilibrative nucleoside transporter 1 OS=Homo sapiens OX=9606 GN=SLC29A1 PE=1 SV=3	SLC29A1	Q99808	0.450372	7281903	-0.34643
9606.ENSP00000354947	F-actin-capping protein subunit alpha-2 OS=Homo sapiens OX=9606 GN=CAPZA2 PE=1 SV=3	CAPZA2	P47755	0.455034	5.26E+07	-0.34196

9606.ENSP00000315386	Protein O-glucosyltransferase 3 OS=Homo sapiens OX=9606 GN=POGLUT3 PE=1 SV=2	KDELC2	Q7Z4H8	0.460557	7627803	-0.33672
9606.ENSP00000377941	Alpha-actinin-1 OS=Homo sapiens OX=9606 GN=ACTN1 PE=1 SV=2	ACTN1	P12814	0.467939	9.04E+08	-0.32981
9606.ENSP00000464087	E3 ubiquitin-protein ligase RNF213 OS=Homo sapiens OX=9606 GN=RNF213 PE=1 SV=3	RNF213	Q63HN8	0.470209	1416454	-0.32771
9606.ENSP00000323967	SWI/SNF-related matrix-associated actin-dependent regulator of chromatin subfamily E member 1 OS=Homo sapiens OX=9606 GN=SMARCE1 PE=1 SV=2	SMARCE1	Q969G3	0.474408	3972464	-0.32385
9606.ENSP00000281537	Tight junction protein ZO-1 OS=Homo sapiens OX=9606 GN=TJP1 PE=1 SV=3	TJP1	Q07157	0.475476	1.29E+07	-0.32287
9606.ENSP00000005386	RNA polymerase II-associated protein 3 OS=Homo sapiens OX=9606 GN=RPAP3 PE=1 SV=2	RPAP3	Q9H6T3	0.475929	3307632	-0.32246
9606.ENSP00000209875	Chromobox protein homolog 5 OS=Homo sapiens OX=9606 GN=CBX5 PE=1 SV=1	CBX5	P45973	0.486098	2.05E+07	-0.31328
9606.ENSP00000393313	Epidermal growth factor receptor substrate 15-like 1 OS=Homo sapiens OX=9606 GN=EPS15L1 PE=1 SV=1	EPS15L1	Q9UBC2	0.491217	3.26E+07	-0.30873
9606.ENSP00000362273	ADP-ribosylhydrolase ARH3 OS=Homo sapiens OX=9606 GN=ADPRS PE=1 SV=1	ADPRHL2	Q9NX46	0.493164	5480098	-0.30701
9606.ENSP00000389631	Actin-related protein 2/3 complex subunit 1B OS=Homo sapiens OX=9606 GN=ARPC1B PE=1 SV=3	ARPC1B	O15143	0.497563	1.31E+08	-0.30315
9606.ENSP00000343701	Importin subunit alpha-5 OS=Homo sapiens OX=9606 GN=KPNA1 PE=1 SV=3	KPNA1	P52294	0.503533	1.68E+07	-0.29797

Table S16. The enrichment analysis of down-regulated proteins

category	description	FDR value
GO Biological Process	RNA splicing	7.47E-13
GO Biological Process	RNA processing	5.59E-12
GO Biological Process	Gene expression	1.59E-11
GO Biological Process	mRNA processing	2.33E-11
GO Biological Process	mRNA metabolic process	2.33E-11
GO Biological Process	RNA splicing, via transesterification reactions	1.91E-10
GO Biological Process	RNA metabolic process	5.37E-10
GO Biological Process	Heterocycle metabolic process	3.11E-09
GO Biological Process	Nucleobase-containing compound metabolic process	3.49E-09
GO Biological Process	mRNA splicing, via spliceosome	3.52E-09
GO Biological Process	Macromolecule metabolic process	9.00E-09

GO Biological Process	Cellular aromatic compound metabolic process	1.09E-08
GO Biological Process	Cellular nitrogen compound metabolic process	1.09E-08
GO Biological Process	Nucleic acid metabolic process	1.14E-08
GO Biological Process	Cellular metabolic process	2.07E-08
GO Biological Process	Organic cyclic compound metabolic process	9.36E-08
GO Biological Process	Nitrogen compound metabolic process	1.14E-07
GO Biological Process	Metabolic process	4.16E-07
GO Biological Process	Organic substance metabolic process	1.20E-06
GO Biological Process	Primary metabolic process	2.84E-06
GO Biological Process	Regulation of mRNA processing	9.87E-05
GO Biological Process	Cellular component organization or biogenesis	2.60E-04
GO Biological Process	Establishment of localization in cell	2.60E-04
GO Biological Process	Ribonucleoprotein complex export from nucleus	5.40E-04
GO Biological Process	Cellular process	5.40E-04
GO Biological Process	Cellular localization	6.30E-04
GO Biological Process	Cellular macromolecule metabolic process	6.30E-04
GO Biological Process	Establishment of protein localization	6.30E-04
GO Biological Process	Intracellular transport	6.30E-04
GO Biological Process	Nucleus organization	7.00E-04
GO Biological Process	mRNA export from nucleus	7.20E-04
GO Biological Process	Protein transport	0.0016
GO Biological Process	Organelle organization	0.0017
GO Biological Process	mRNA transport	0.0018
GO Biological Process	Intracellular protein transport	0.0019
GO Biological Process	RNA localization	0.0023
GO Biological Process	Regulation of mRNA metabolic process	0.0023
GO Biological Process	Cellular component organization	0.0023
GO Biological Process	Nucleobase-containing compound transport	0.0032
GO Biological Process	RNA transport	0.0033
GO Biological Process	Vesicle-mediated transport	0.0037
GO Biological Process	Cellular protein localization	0.0037
GO Biological Process	Positive regulation of organelle organization	0.0049
GO Biological Process	Nucleocytoplasmic transport	0.0049
GO Biological Process	Protein localization	0.0052
GO Biological Process	Regulation of organelle organization	0.0056
GO Biological Process	Transcription elongation from RNA polymerase II promoter	0.0068
GO Biological Process	Vesicle organization	0.007
GO Biological Process	Regulation of RNA splicing	0.0075
GO Biological Process	Cellular protein metabolic process	0.0075
GO Biological Process	Nitrogen compound transport	0.0079
GO Biological Process	Protein-containing complex localization	0.0125
GO Biological Process	Viral life cycle	0.019
GO Biological Process	Regulation of mRNA 3-end processing	0.0237
GO Biological Process	ncRNA metabolic process	0.0266
GO Biological Process	Regulation of histone H3-K4 methylation	0.0266
GO Biological Process	Regulation of histone methylation	0.0281
GO Biological Process	Golgi vesicle transport	0.0325
GO Biological Process	Regulation of intracellular transport	0.033
GO Biological Process	Symbiotic process	0.033

GO Biological Process	Protein-containing complex subunit organization	0.0337
GO Biological Process	Multivesicular body organization	0.0365
GO Biological Process	Endosome organization	0.0384
GO Biological Process	Protein K48-linked ubiquitination	0.0411
GO Biological Process	Regulation of protein transport	0.0434
GO Biological Process	Intracellular transport of virus	0.0439
KEGG Pathways	Spliceosome	6.00E-04
KEGG Pathways	Endocytosis	0.0054
KEGG Pathways	RNA transport	0.0062

Table S17. The GO analysis for OrbiSIMS signatures

GO_ID	GO_DESCRIPTION	GO_LINKED_COMPOUNDS	GO_LINKED_COMPOUNDS_TOTAL	INPUT_COMPOUNDS	OVERLAP	PVALUE_HYPER_RAW	PVALUE_ADJ_FDR
GO:0046439	L-cysteine metabolic process	23	2212	15	4	1.126E-05	0.00059592
GO:0019448	L-cysteine catabolic process	23	2212	15	4	1.126E-05	0.00059592
GO:0009093	cysteine catabolic process	23	2212	15	4	1.126E-05	0.00059592
GO:0000098	sulfur amino acid catabolic process	35	2212	15	4	6.3452E-05	0.00218276
GO:0009071	serine family amino acid catabolic process	55	2212	15	4	0.00038133	0.0093697
GO:0006534	cysteine metabolic process	59	2212	15	4	0.00050071	0.01023947
GO:0070127	tRNA aminoacylation for mitochondrial protein translation	26	2212	15	3	0.00059779	0.01028206
GO:0043038	amino acid activation	69	2212	15	4	0.00091342	0.01047384
GO:0006538	glutamate catabolic process	29	2212	15	3	0.00082988	0.01047384
GO:0016485	protein processing	67	2212	15	4	0.00081643	0.01047384

GO:0043039	tRNA aminoacylation	69	2212	15	4	0.00091342	0.01047384
GO:0006418	tRNA aminoacylation for protein translation	64	2212	15	4	0.00068504	0.01047384
GO:0006497	protein lipidation	37	2212	15	3	0.00170785	0.01546057
GO:0019363	pyridine nucleotide biosynthetic process	50	2212	15	3	0.00408472	0.02509188
GO:0009435	NAD biosynthetic process	50	2212	15	3	0.00408472	0.02509188
GO:0019359	nicotinamide nucleotide biosynthetic process	50	2212	15	3	0.00408472	0.02509188
GO:0019370	leukotriene biosynthetic process	49	2212	15	3	0.00385541	0.02509188

Table S18. The GO analysis for LC-MS/MS signatures

**GO_ID**	**GO_DESCRIPTION**	**GO_LINKED_COMPOUNDS**	**GO_LINKED_COMPOUNDS_TOTAL**	**INPUT_COMPOUNDS**	**OVERLAP**	**PVALUE_HYPER_RAW**	**PVALUE_ADJ_FDR**
GO:0072526	pyridine-containing compound catabolic process	34	2212	28	7	9.9703E-08	7.6373E-06
GO:0072524	pyridine-containing compound metabolic process	181	2212	28	12	6.001E-07	3.8307E-05
GO:0046439	L-cysteine metabolic process	23	2212	28	5	6.4327E-06	0.00020531
GO:0019448	L-cysteine catabolic process	23	2212	28	5	6.4327E-06	0.00020531
GO:0009093	cysteine catabolic process	23	2212	28	5	6.4327E-06	0.00020531

GO:0008652	cellular amino acid biosynthetic process	234	2212	28	12	9.697E-06	0.00025306
GO:0006166	purine ribonucleoside salvage	43	2212	28	6	1.0333E-05	0.00025306
GO:0009064	glutamine family amino acid metabolic process	161	2212	28	10	1.3214E-05	0.00028116
GO:0000096	sulfur amino acid metabolic process	99	2212	28	8	1.7958E-05	0.0003439
GO:0034656	nucleobase-containing small molecule catabolic process	80	2212	28	7	3.9862E-05	0.00056545
GO:0043101	purine-containing compound salvage	55	2212	28	6	4.4283E-05	0.00060573
GO:0000098	sulfur amino acid catabolic process	35	2212	28	5	5.5875E-05	0.00069032
GO:0006534	cysteine metabolic process	59	2212	28	6	6.648E-05	0.000724
GO:0046090	deoxyadenosine metabolic process	37	2212	28	5	7.3723E-05	0.000724
GO:0046094	deoxyinosine metabolic process	37	2212	28	5	7.3723E-05	0.000724
GO:0046102	inosine metabolic process	37	2212	28	5	7.3723E-05	0.000724
GO:0046124	purine deoxyribonucleoside catabolic process	37	2212	28	5	7.3723E-05	0.000724
GO:0006148	inosine catabolic process	37	2212	28	5	7.3723E-05	0.000724
GO:0006149	deoxyinosine catabolic process	37	2212	28	5	7.3723E-05	0.000724
GO:0	deoxyaden	37	2212	28	5	7.3723E-05	0.000724

0061 57	osine catabolic process						
GO:0 0431 74	nucleoside salvage	61	2212	28	6	8.0515E-05	0.000752 13
GO:0 0430 96	purine nucleobas e salvage	20	2212	28	4	8.6711E-05	0.000790 73
GO:0 0065 36	glutamate metabolic process	93	2212	28	7	0.00010638	0.000831 52
GO:0 0424 51	purine nucleoside biosynthet ic process	64	2212	28	6	0.00010592	0.000831 52
GO:0 0424 55	ribonucleo side biosynthet ic process	64	2212	28	6	0.00010592	0.000831 52
GO:0 0461 29	purine ribonucleo side biosynthet ic process	64	2212	28	6	0.00010592	0.000831 52
GO:0 0461 30	purine ribonucleo side catabolic process	39	2212	28	5	9.5687E-05	0.000831 52
GO:0 0430 94	cellular metabolic compound salvage	95	2212	28	7	0.00012198	0.000901 38
GO:0 0061 52	purine nucleoside catabolic process	41	2212	28	5	0.00012238	0.000901 38
GO:0 0461 22	purine deoxyribo nucleoside metabolic process	41	2212	28	5	0.00012238	0.000901 38
GO:0 0322 63	GMP salvage	22	2212	28	4	0.00012865	0.000920 87
GO:0 0091 64	nucleoside catabolic process	67	2212	28	6	0.00013736	0.000956 52
GO:0 0091 13	purine nucleobas e biosynthet ic process	43	2212	28	5	0.00015447	0.001037 91
GO:0 0061 77	GMP biosynthet ic process	45	2212	28	5	0.00019265	0.001250 58
GO:0 0461	purine ribonucleo	105	2212	28	7	0.00023061	0.001472 08

28	side metabolic process						
GO:0046110	xanthine metabolic process	26	2212	28	4	0.0002539	0.00154356
GO:0006154	adenosine catabolic process	26	2212	28	4	0.0002539	0.00154356
GO:0042454	ribonucleoside catabolic process	48	2212	28	5	0.00026299	0.00154961
GO:0046121	deoxyribonucleoside catabolic process	48	2212	28	5	0.00026299	0.00154961
GO:0046083	adenine metabolic process	27	2212	28	4	0.00029547	0.00164116
GO:0106380	purine ribonucleotide salvage	27	2212	28	4	0.00029547	0.00164116
GO:0044209	AMP salvage	27	2212	28	4	0.00029547	0.00164116
GO:0009119	ribonucleoside metabolic process	110	2212	28	7	0.00030887	0.00168997
GO:0009155	purine deoxyribonucleotide catabolic process	78	2212	28	6	0.00032127	0.00170895
GO:0006167	AMP biosynthetic process	50	2212	28	5	0.00031976	0.00170895
GO:0042278	purine nucleoside metabolic process	111	2212	28	7	0.00032684	0.0017148
GO:0034404	nucleobase-containing small molecule biosynthetic process	81	2212	28	6	0.00039546	0.0019929
GO:0009163	nucleoside biosynthetic process	81	2212	28	6	0.00039546	0.0019929
GO:0009168	purine ribonucleoside monophosphate biosynthet	53	2212	28	5	0.00042184	0.00207133

	ic process						
GO:0009071	serine family amino acid catabolic process	55	2212	28	5	0.00050248	0.00241037
GO:0016226	iron-sulfur cluster assembly	32	2212	28	4	0.00057955	0.00269839
GO:0009151	purine deoxyribonucleotide metabolic process	88	2212	28	6	0.00062125	0.0028326
GO:0046112	nucleobase biosynthetic process	59	2212	28	5	0.00069818	0.00314592
GO:1901659	glycosyl compound biosynthetic process	91	2212	28	6	0.00074437	0.00321292
GO:0009127	purine nucleoside monophosphate biosynthetic process	60	2212	28	5	0.00075499	0.00321292
GO:0046059	dAMP catabolic process	60	2212	28	5	0.00075499	0.00321292
GO:0009172	purine deoxyribonucleoside monophosphate catabolic process	60	2212	28	5	0.00075499	0.00321292
GO:0009162	deoxyribonucleoside monophosphate metabolic process	93	2212	28	6	0.00083649	0.00344489
GO:0046040	IMP metabolic process	93	2212	28	6	0.00083649	0.00344489
GO:0006204	IMP catabolic process	62	2212	28	5	0.00087897	0.00358135
GO:0009394	2'-deoxyribonucleotide metabolic process	131	2212	28	7	0.000904	0.0035828
GO:0032261	purine nucleotide salvage	36	2212	28	4	0.00091675	0.0035828
GO:0	deoxyribo	134	2212	28	7	0.00103601	0.003852

0092 62	nucleotide metabolic process						34
GO:0 0196 92	deoxyribo se phosphate metabolic process	134	2212	28	7	0.00103601	0.003852 34
GO:0 0460 53	dAMP metabolic process	64	2212	28	5	0.00101763	0.003852 34
GO:0 0002 55	allantoin metabolic process	65	2212	28	5	0.00109279	0.003986 08
GO:0 0061 88	IMP biosynthet ic process	38	2212	28	4	0.00112891	0.004078 98
GO:0 0091 70	purine deoxyribo nucleoside monophos phate metabolic process	66	2212	28	5	0.00117202	0.004167 96
GO:0 0091 20	deoxyribo nucleoside metabolic process	67	2212	28	5	0.00125546	0.004371 29
GO:0 0461 00	hypoxanth ine metabolic process	39	2212	28	4	0.00124699	0.004371 29
GO:0 0092 64	deoxyribo nucleotide catabolic process	103	2212	28	6	0.00143778	0.004830 42
GO:0 0463 86	deoxyribo se phosphate catabolic process	103	2212	28	6	0.00143778	0.004830 42
GO:0 0091 28	purine nucleoside monophos phate catabolic process	69	2212	28	5	0.00143552	0.004830 42
GO:0 0091 69	purine ribonucleo side monophos phate catabolic process	69	2212	28	5	0.00143552	0.004830 42
GO:0 0094 49	gamma- aminobuty ric acid biosynthet ic process	72	2212	28	5	0.00174067	0.005602 34

GO:0042136	neurotransmitter biosynthetic process	73	2212	28	5	0.00185231	0.00581505
GO:0009167	purine ribonucleoside monophosphate metabolic process	108	2212	28	6	0.00184179	0.00581505
GO:0009126	purine nucleoside monophosphate metabolic process	110	2212	28	6	0.00202573	0.00630775
GO:0009159	deoxyribonucleoside monophosphate catabolic process	77	2212	28	5	0.00235235	0.00701951
GO:0043605	cellular amide catabolic process	114	2212	28	6	0.00243546	0.00712048
GO:0006147	guanine catabolic process	23	2212	28	3	0.00271683	0.00776528
GO:0042453	deoxyguanosine metabolic process	23	2212	28	3	0.00271683	0.00776528
GO:0006161	deoxyguanosine catabolic process	23	2212	28	3	0.00271683	0.00776528
GO:0042219	cellular modified amino acid catabolic process	80	2212	28	5	0.00278766	0.00790869
GO:0009448	gamma-aminobutyric acid metabolic process	81	2212	28	5	0.00294504	0.00823322
GO:0043173	nucleotide salvage	51	2212	28	4	0.00341188	0.00938791
GO:0043649	dicarboxylic acid catabolic process	84	2212	28	5	0.00345612	0.00938791
GO:0009156	ribonucleoside monophosphate biosynthet	84	2212	28	5	0.00345612	0.00938791

	ic process						
GO:0009158	ribonucleoside monophosphate catabolic process	84	2212	28	5	0.00345612	0.00938791
GO:0009125	nucleoside monophosphate catabolic process	86	2212	28	5	0.0038308	0.01011859
GO:0046055	dGMP catabolic process	55	2212	28	4	0.00449668	0.0112564
GO:0046037	GMP metabolic process	90	2212	28	5	0.00466708	0.0116071
GO:0006196	AMP catabolic process	56	2212	28	4	0.00480051	0.01186191
GO:0046054	dGMP metabolic process	57	2212	28	4	0.00511796	0.01225113
GO:0006750	glutathione biosynthetic process	31	2212	28	3	0.00644346	0.01451674
GO:0046487	glyoxylate metabolic process	31	2212	28	3	0.00644346	0.01451674
GO:0006145	purine nucleobase catabolic process	31	2212	28	3	0.00644346	0.01451674
GO:0046038	GMP catabolic process	31	2212	28	3	0.00644346	0.01451674
GO:0046033	AMP metabolic process	98	2212	28	5	0.00672116	0.01505383
GO:0072525	pyridine-containing compound biosynthetic process	62	2212	28	4	0.00691926	0.01531835
GO:0046098	guanine metabolic process	34	2212	28	3	0.00836279	0.01789357
GO:0000097	sulfur amino acid biosynthetic process	66	2212	28	4	0.008633	0.0182677
GO:0016485	protein processing	67	2212	28	4	0.00910141	0.01884239
GO:0019184	nonribosomal peptide	35	2212	28	3	0.00906974	0.01884239

	biosynthetic process						
GO:0009065	glutamine family amino acid catabolic process	70	2212	28	4	0.0106064	0.02115755
GO:0009226	nucleotide-sugar biosynthetic process	38	2212	28	3	0.01139735	0.02238556
GO:0046085	adenosine metabolic process	72	2212	28	4	0.01169508	0.02273714
GO:0046113	nucleobase catabolic process	39	2212	28	3	0.01224321	0.02356357
GO:0046184	aldehyde biosynthetic process	41	2212	28	3	0.01404157	0.02649223
GO:0097052	L-kynurenine metabolic process	42	2212	28	3	0.01499454	0.02815151
GO:0019674	NAD metabolic process	79	2212	28	4	0.01606937	0.03002228
GO:0006555	methionine metabolic process	45	2212	28	3	0.0180708	0.03343535
GO:0046073	dTMP metabolic process	48	2212	28	3	0.02147605	0.03843611
GO:0009225	nucleotide-sugar metabolic process	50	2212	28	3	0.02393018	0.04092556
GO:0019363	pyridine nucleotide biosynthetic process	50	2212	28	3	0.02393018	0.04092556
GO:0009435	NAD biosynthetic process	50	2212	28	3	0.02393018	0.04092556
GO:0019359	nicotinamide nucleotide biosynthetic process	50	2212	28	3	0.02393018	0.04092556
GO:0070189	kynurenine metabolic process	53	2212	28	3	0.02788806	0.04603934
GO:0006586	indolalkylamine metabolic process	55	2212	28	3	0.03071098	0.04861123

GO:0006103	2-oxoglutarate metabolic process	55	2212	28	3	0.03071098	0.04861123

References

1. García-Morales, V., et al., *Membrane-derived phospholipids control synaptic neurotransmission and plasticity.* PLoS biology, 2015. **13**(5): p. e1002153-e1002153.
2. Vance, J.E., *Phospholipid Synthesis and Transport in Mammalian Cells.* Traffic, 2015. **16**(1): p. 1-18.
3. Bhattacharya, A., et al., *A minimal biochemical route towards de novo formation of synthetic phospholipid membranes.* Nature Communications, 2019. **10**(1): p. 300.
4. Taniguchi, M. and T. Okazaki, *The role of sphingomyelin and sphingomyelin synthases in cell death, proliferation and migration-from cell and animal models to human disorders.* Biochim Biophys Acta, 2014. **1841**(5): p. 692-703.
5. Huxtable, R.J., *Taurine in the central nervous system and the mammalian actions of taurine.* Progress in Neurobiology, 1989. **32**(6): p. 471-533.
6. Muthuraman, A., et al., *Physiological and Pathophysiological Role of Cysteine Metabolism in Human Metabolic Syndrome.* Drug Metab Lett, 2021. **14**(3): p. 177-192.
7. Edney, M.K., et al., *Molecular Formula Prediction for Chemical Filtering of 3D OrbiSIMS Datasets.* Analytical Chemistry, 2022. **94**(11): p. 4703-4711.
8. Kotowska, A.M., et al., *Protein identification by 3D OrbiSIMS to facilitate in situ imaging and depth profiling.* Nature Communications, 2020. **11**(1): p. 5832.
9. Ramakrishna, S., et al., *APOE4 Affects Basal and NMDAR-Mediated Protein Synthesis in Neurons by Perturbing Calcium Homeostasis.* The Journal of Neuroscience, 2021. **41**(42): p. 8686-8709.
